# Near Linearity of the Macroscopic Hall Current Response in Infinitely Extended Gapped Fermion Systems

**DOI:** 10.1007/s00220-025-05361-y

**Published:** 2025-07-02

**Authors:** Marius Wesle, Giovann Marcelli, Tadahiro Miyao, Domenico Monaco, Stefan Teufel

**Affiliations:** 1 Fachbereich Mathematik, Eberhard Karls Universität Tübingen, Auf der Morgenstelle 10, 72076 Tübingen, Germany; 2 Dipartimento di Matematica e Fisica, Università di Roma Tre, L.go S. L. Murialdo 1, 00146 Roma, Italy; 3 Department of Mathematics, Hokkaido University, Sapporo, 060-0810 Japan; 4 Dipartimento di Matematica “Guido Castelnuovo”, Sapienza Università di Roma, Piazzale Aldo Moro 5, 00185 Roma, Italy

## Abstract

We consider an infinitely extended system of fermions on a *d*-dimensional lattice with (magnetic) translation-invariant short-range interactions. We further assume that the system has a gapped ground state. Physically, this is a model for the bulk of a generic topological insulator at zero temperature, and we are interested in the current response of such a system to a constant external electric field. Using the *non-equilibrium almost-stationary states* approach, we prove that the longitudinal current density induced by a constant electric field of strength $\varepsilon$ is of order $\mathcal {O}(\varepsilon ^\infty )$, i.e. the system is an insulator in the usual sense. For the Hall current density we show instead that it is linear in $\varepsilon$ up to terms of order $\mathcal {O}(\varepsilon ^\infty )$. The proportionality factor $\sigma _\textrm{H}$ is by definition the Hall conductivity, and we show that it is given by a generalization of the well known double commutator formula to interacting systems. As a by-product of our results, we find that the Hall conductivity is constant within gapped phases, and that for $d=2$ the relevant observable that “measures” the Hall conductivity in experiments, the Hall conductance, not only agrees with $\sigma _{\textrm{H}}$ in expectation up to $\mathcal {O}(\varepsilon ^\infty )$, but also has vanishing variance. A notable difference to several existing results on the current response in interacting fermion systems is that we consider a macroscopic system exposed to a small constant electric field, rather than to a small voltage drop.

## Introduction

Understanding the experimentally observed quantization of Hall conductance at low temperatures has inspired a great deal of work in mathematics and theoretical physics. As a theoretical problem, the quantum Hall effect has many different interesting aspects, and our work deals with one of them. In a nutshell, the theorems we prove here show that the conductivity model usually assumed inside the incompressible stripes of a Hall bar can be derived from a semi-realistic microscopic model with very high accuracy. More fundamentally, we also prove that insulators do indeed insulate, even when the applied voltage exceeds the spectral gap by many orders of magnitude. We work directly on models in infinite volume, avoiding the complications caused by boundaries, but without the additional restrictions inherent in torus geometries. As a consequence, our results concern only the bulk behaviour of topological insulators, but not the equally important edge physics. Conceptually, but not technically, our results are generalizations of recent results [37] on fermion systems of non-interacting particles to the case with interactions. We postpone a more detailed discussion of their physical significance and their relation to the vast existing literature on this subject to the end of the introduction.

Instead, let us first sketch the mathematical setup and state our main theorems in a somewhat informal way. In our work we consider an infinitely extended electron lattice gas from the outset, instead of the more common approach in related work (e.g. [7, 8, 16] and many others) of analyzing finite systems uniformly in the system size. To mathematically describe such infinite systems, many pioneers have developed a precise framework based on operator algebras (see, e.g., [2, 13, 14] and references therein), and we adopt this framework in our study. The advantages of this approach in our context will be discussed in detail later in this section. To model an infinitely extended gas of electrons in a periodic structure with constant density, we take the one-body position space to be the lattice $\mathbb {Z}^d$, where $d\in \mathbb {N}$ is the spatial dimension, and the one-body Hilbert space to be $\ell ^2(\mathbb {Z}^d,\mathbb {C}^n)$, where $\mathbb {C}^n$ models the spin of the electrons and possible other degrees of freedom arising from a non-trivial structure of the unit cell. The observable algebra for the many-body system is the corresponding CAR algebra denoted by $\mathcal {A}$, which is a subalgebra of the algebra of bounded operators on the fermionic Fock space $\mathcal {F}(\mathbb {Z}^d,\mathbb {C}^n)$ (see Sect. 2.1 for details). The Heisenberg dynamics of the unperturbed system is generated by a densely defined derivation $\mathcal {L}_{H}$ of $\mathcal {A}$ coming from a translation invariant Hamiltonian *H* with short-range interactions that conserves the number of particles. An important novelty in this context is that we require translation invariance only with respect to a very general form of translations (see Definition 2.7), including in particular magnetic translations. For example, our assumptions also include the many-body version of the Hofstadter model with arbitrary magnetic field strength and weak interactions, see Sect. 3 for details.

The essential assumption for our analysis is the so-called incompressibility of the ground state, that is, the assumption that $\mathcal {L}_{H}$ has a gapped ground state $\omega _0$ such that the associated GNS Hamiltonian has a spectral gap $g>0$ above its lowest eigenvalue zero (compare Definition 3.1). This assumption can be proved so far only for non-interacting or weakly interacting fermion systems, see [16, 22, 23, 34] and Proposition 3.4, but is considered to be the defining property of (topological) insulators.

The perturbation by a constant external electric field of strength $\varepsilon \in \mathbb {R}$ is modeled by adding to $\mathcal {L}_{H}$ the densely defined derivation $\mathcal {L}_{\varepsilon X_1}$, where $X_1$ denotes the first component of the second quantization of the position operator. Note that neither *H* nor $X_1$ are bounded operators on Fock space, but *H* is a bounded sum-of-local-terms (SLT) interaction, while $X_1$ is an unbounded SLT interaction. It is shown in [10, 28, 46], that if the system starts in the gapped ground state $\omega _0$ and then the perturbation $\mathcal {L}_{\varepsilon X_1}$ is adiabatically switched on, the system dynamically evolves up to errors of order $\mathcal {O}(\varepsilon ^{\infty })$ into a non-equilibrium almost-stationary state (NEASS) $\omega _\varepsilon$.^1^

See for example [27, 36, 39] for a discussion of the NEASS approach to linear response theory. Note, however, that our results rely on only two properties of $\omega _\varepsilon$, namely that it is an almost stationary state for the perturbed dynamics generated by $\mathcal {L}_{H+\varepsilon X_1}$, and that it is connected to $\omega _0$ by a quasi-local automorphism. For our purposes, it is not necessary to explicitly compute $\omega _\varepsilon$ or its asymptotic expansion in powers of $\varepsilon$.

Our main result, Theorem 5.1, concerns the current density in the state $\omega _\varepsilon$. Let $\textrm{i}[H,X_k] = \sum _{x\in \mathbb {Z}^d} j_{x,k}$ be a decomposition of the translation invariant current operator^2^$\textrm{i}[H,X_k]$, so $j_{x,k}\in \mathcal {A}$ denotes the *k*-th component of the current operator at location $x\in \mathbb {Z}^d$. Then we show that for all $x\in \mathbb {Z}^d$

1 $$\begin{aligned} \omega _\varepsilon (j_{x,1}) = \mathcal {O}(\varepsilon ^\infty )\,, \end{aligned}$$

and that for $k\not =1$

2 $$\begin{aligned} \omega _\varepsilon (j_{x,k}) = \varepsilon \,\overline{\omega _0}(\textrm{i}[X_k^{\textrm{OD}},X_1^{\textrm{OD}}]) + \mathcal {O}(\varepsilon ^\infty )\,. \end{aligned}$$

In the above,

- $\overline{\omega _0}(\cdot )$ denotes an expectation in the equilibrium ground state taken per unit volume (compare (5) for a precise definition), and
- the off-diagonal part $X^{\textrm{OD}}$ of the position operator (and of a more general class of interactions) with respect to the state $\omega _0$ is discussed in Sect. 4, see Definition 10.

Note that while the definition of local current operators for lattice systems in general involves some arbitrariness, in our periodic setting the left-hand sides of (1) and (2) are unambiguously defined.

The Hall conductivity, defined as the ratio between the induced current density and the applied electric field in the limit of vanishing field strength, is thus given by

3 $$\begin{aligned} \sigma ^\textrm{H}_{k1}= \overline{\omega _0}(\textrm{i}[X_k^{\textrm{OD}},X_1^{\textrm{OD}}])\,. \end{aligned}$$

Equation (1) shows that the current density in the direction of the applied field vanishes faster than any power of $\varepsilon$, while (2) shows that the Hall current density is a nearly linear function of the applied field near zero. Note that the expression on the right-hand side of (3) is the generalization to the interacting case of the so-called double commutator formula for the Hall conductivity:

$$\begin{aligned} \overline{\textrm{tr}}\left( \textrm{i}P_0[ [{\hat{x}}_k, P_0],[{\hat{x}}_1,P_0]]\right) \end{aligned}$$

which holds for non-interacting systems (see [37] and references therein). Here $\overline{\omega _0}(\cdot )$ replaces the trace per unit volume $\overline{\textrm{tr}}(P_0\,\cdot )$, where $P_0$ is the infinite rank Fermi projection, and $X_k^{\textrm{OD}}$ replaces off-diagonal part of the *k*-th component of the one-body position operator ${\hat{x}}_k$ with respect to $P_0$.

Formula (3) already suggests, as we indeed prove (Corollary 5.3), that the Hall conductivity depends only on the state $\omega _0$ but not on the Hamiltonian *H*, although the off-diagonal part $X_k^{\textrm{OD}}$ is defined using *H*, see Definition 10. Even more, as a simple corollary to a many-body Chern–Simons formula, Lemma 5.2, which is involved in our proof, we find that the Hall conductivity $\sigma ^\textrm{H}_{k1}$ is constant within gapped phases of ground states (Corollary 5.3). Our results also allow a generalization to irrational magnetic fields of the results of Giuliani, Mastropietro and Porta [21, 22], i.e. the stability of the Hall conductivity with respect to the introduction of small interactions, by a completely different approach. Since the proof of the continuity of the Hall conductivity with respect to the magnetic field requires additional technical effort, we provide the details in another paper [35].

Furthermore, using the results of Sopenko and Kapustin [31], we show that the Hall conductivity $\sigma ^\textrm{H}_{k1}$ takes quantized values not only in the non-interacting phase, but in any invertible phase (Corollary 5.4).

From (2) we also conclude that for $d=2$ the expectation of the Hall conductance observable

$$\begin{aligned} G^\textrm{H}_{21,L}:= \frac{J_L}{\varepsilon L}, \end{aligned}$$

defined as the ratio of the current operator $J_L$ through a line segment of length *L* aligned with the applied field and the voltage drop $\varepsilon L$ across this line segment, not only agrees with the Hall conductivity $\sigma ^\textrm{H}_{21}$, but also has a vanishing variance in the limit $L\rightarrow \infty$:

$$\begin{aligned}  &   \omega _\varepsilon \hspace{-2pt}\left( G^\textrm{H}_{21,L}\right) = \sigma ^\textrm{H}_{21} + \mathcal {O}(\varepsilon ^\infty ), \quad \quad \text{ and }\quad \quad \\  &   \quad \textrm{var}\left( G^\textrm{H}_{21,L}\right) := \omega _\varepsilon \hspace{-2pt}\left( \left( G^\textrm{H}_{21,L}\right) ^2\right) -\left( \omega _\varepsilon \hspace{-2pt}\left( G^\textrm{H}_{21,L}\right) \right) ^2=\mathcal {O}\left( \frac{1}{\varepsilon ^2 L}\right) . \end{aligned}$$

Note that such a statement is relevant in explaining the experimental findings: It is not only the average value $\sigma ^\textrm{H}_{21}$ of $G^\textrm{H}_{21,L}$ that is found to be quantized with an accuracy of approximately nine decimal digits, but there are also no measurable fluctuations around this value for *L* sufficiently large. As far as we know, this has not been demonstrated before in any model of interacting electrons. Note that the factor $1/\varepsilon ^2$ in the bound on the variance of $G^\textrm{H}_{21,L}$ is unavoidable, since even for $\varepsilon =0$ the fluctuations of $J_L$ in the ground state $\omega _0$ are non-vanishing.

The precise formulation of our results requires some technical preparation and is therefore postponed to Sect. 5. While we explicitly consider the case of lattice fermions, we note that the method presented in this paper can also be applied to quantum spin systems with a conserved charge.

In the remainder of this introduction, we will comment on the existing literature related to our results, the main new mathematical challenges encountered in our proofs, and their physical significance.

The mathematical and theoretical physics literature on the quantum Hall effect has grown steadily over the last 40 years and is now vast. We have therefore chosen to focus on a small selection of results that we consider most relevant to put our results into context. Our results are concerned with deriving a formula for the Hall conductivity and the Hall conductance at zero temperature from a microscopic model, an enterprise often referred to as proving linear response or proving Kubo’s formula for gapped systems at zero temperature (see e.g. [6, 27] for recent reviews). A problem we do not address here is that of proving that the resulting formula gives only quantized values (see [5, 25, 31] and references therein, or [18, 19] for a general approach based on effective actions). Much of the earlier mathematical work in this area has been concerned with the free electron gas, i.e. with non-interacting models, often with a focus on the role of random impurities (see e.g. [1, 11, 12, 37]); some authors have considered weakly interacting electrons [22], but we consider interacting fermions without any smallness assumption on the interaction (let us stress once again that, however, the incompressibility assumption on the existence of a gapped ground state has been proven only in the weakly-interacting regime). Most other results on interacting models consider finite systems with a torus geometry to avoid the problem that, in topological insulators, edge states close the spectral gap. Due to the lack of a position operator on such a geometry, the external electric field in this setting is introduced by so-called adiabatic flux threading [4, 6, 8, 32, 33]. Instead, we work directly in the infinite volume and require only a gap in the bulk, a concept introduced in [41]. Thus, the main novelty of our setup is that we start from an infinite system of interacting particles, require only a gap in the bulk, and model the driving field by a linear potential $\varepsilon X_1$. It turns out that our approach allows transparent and simple arguments and covers relevant aspects of the real experimental situation. In fact, after establishing the appropriate technical framework and proving the existence and properties of the NEASS $\omega _\varepsilon$ for infinite volume systems without uniqueness assumption on the ground state in Proposition 3.3, the proof of our main theorem, Theorem 5.1, consists only of a short calculation and an application of a new many-body variant of the Chern–Simons lemma, Lemma 5.2.

Note that also for interacting lattice fermions it was shown before in [8], based on the approach of [32], that power law corrections to Kubo’s formula vanish, i.e. that the Hall current response is nearly linear in the driving strength $\varepsilon$ up to terms that are asymptotically smaller than any power of $\varepsilon$. As mentioned above, the authors of [8] consider a system of interacting lattice fermions on a finite two-dimensional flat torus, but give error bounds that are uniform in the linear system size *L*. The main difference from our approach is that, instead of applying a constant electric field of strength $\varepsilon$ throughout the system, the electromotive force is applied in [8] by a time-dependent gauge potential that varies at rate $\varepsilon$. The way this is done in [8] translates to applying a potential step of height $\varepsilon$ only over a single line in the system, say the line $\{x_1=0\}$, i.e. to applying an electric field of strength $\varepsilon$ that is supported only on that line. Alternatively, one could choose the time-dependent gauge potential so that an electric field of strength $\varepsilon /L$ is added everywhere.

Mathematically, this is an important difference, since a potential step of height $\varepsilon$ or a constant field of strength $\varepsilon /L$ that decreases with increasing system size are both regular perturbations that do not close the spectral gap of *H* for sufficiently small $\varepsilon$. As a consequence, the result in [8, 32] follows from an appropriate application of the adiabatic theorem of quantum mechanics. Our situation is different: a constant electric field of strength $\varepsilon$ applied to an infinitely extended system is a singular perturbation that closes the spectral gap, no matter how small $\varepsilon \in \mathbb {R}$ is. Thus the standard adiabatic theorem cannot be applied and a local version in space, the so-called space adiabatic theorem, is required, see [28, 44, 46].

Physically, a linear potential drop over a macroscopic region is also closer to real experimental situations, where in quantum Hall bars the Hall voltage is expected and observed to drop essentially linearly over macro- or at least mesoscopic locally gapped regions, so-called incompressible stripes, see [20] for a recent review. An even more basic fact is the following: both in normal and in topological insulators the longitudinal current is observed to vanish even if one applies across the sample a voltage that exceeds the spectral gap by many orders of magnitude. Last but not least, to our understanding it is also necessary to consider the extensive situation of a current flowing in a macroscopic region in order to conclude that the measured Hall conductance has vanishing fluctuations.

The rest of the paper is structured as follows. In Sect. 2 we first introduce the basic technical notions and then collect several technical propositions concerning periodic interactions and, in particular, their commutator with the unbounded position operators. In Sect. 3 we then formulate the precise assumptions underlying our main theorem and establish in Proposition 3.3 the existence and relevant properties of the NEASS. Moreover, we show in Proposition 3.4 that the weakly interacting Hofstadter model, which is basically the discrete Landau operator, satisfies all the assumptions of our main theorem. In Sect. 4 we need to introduce another object, the off-diagonal map, which selects the off-diagonal part of an interaction with respect to a gapped ground state. In particular, it turns out that the off-diagonal part of the position operator is a bounded SLT-interaction. All proofs up to this point are rather technical and should not surprise experts. They are therefore postponed to the appendices. In Sect. 5 we finally state our main theorem, Theorem 5.1, and several corollaries mentioned above. The proof of Theorem 5.1 is given immediately after the statement because, after setting up the technical tools, it is rather short and instructive. Indeed, we consider the simplicity and transparency of the proof to be one of the virtues of our approach. However, as already mentioned, it uses a new Chern–Simons type lemma, the proof of which is also deferred to an appendix.

## Mathematical Setup

We basically use the standard formalism for describing infinitely extended systems of interacting lattice fermions in the thermodynamic limit, as explained for example in [13, 14] and, in addition, more specific results for fermionic systems from [2]. However, it turns out that our analysis requires a number of technical results, mostly concerning periodic interactions and position operators, which were not available in the previous literature. The proofs of the corresponding lemmas and propositions are collected in Appendix B.

### Basic notions

The anti-symmetric (or fermionic) Fock space over the lattice $\mathbb {Z}^d$ is given by

$$\begin{aligned} \mathcal {F}(\mathbb {Z}^d,\mathbb {C}^n) := \bigoplus _{N=0}^{\infty }\ell ^2(\mathbb {Z}^d,\mathbb {C}^n)^{\wedge N} ~, \end{aligned}$$

where $\ell ^2(\mathbb {Z}^d, \mathbb {C}^n)^{\wedge N}$ denotes the *N*-fold anti-symmetric tensor product of $\ell ^2(\mathbb {Z}^d, \mathbb {C}^n)$, with the convention $\ell ^2(\mathbb {Z}^d, \mathbb {C}^n)^{\wedge 0} = \mathbb {C}$. We use $a^*_{x,i}$ and $a_{x,i}$, for $x\in \mathbb {Z}^d$ and $i\in \{1,\dots ,n\}$, to denote the fermionic creation and annihilation operators associated to the standard basis of $\ell ^2(\mathbb {Z}^d,\mathbb {C}^n)$; recall that they satisfy the canonical anti-commutation relations (CAR). The number operator at site $x\in \mathbb {Z}^d$ is defined by

$$\begin{aligned} n_x:=\sum _{i=1}^n a^*_{x,i}a_{x,i}\,. \end{aligned}$$

The algebra of all bounded operators on $\mathcal {F}(\mathbb {Z}^d,\mathbb {C}^n)$ is denoted by $\mathcal {B}(\mathcal {F}(\mathbb {Z}^d,\mathbb {C}^n))$. For each $M\subseteq \mathbb {Z}^d$ let $\mathcal {A}_M$ be the C*-subalgebra of $\mathcal {B}(\mathcal {F}(\mathbb {Z}^d,\mathbb {C}^n))$ generated by

$$\begin{aligned} \{a^*_{x,i}~|~x\in M,~ i\in \{1,\dots ,n\}\}~. \end{aligned}$$

The C*-algebra $\mathcal {A}:= \mathcal {A}_{\mathbb {Z}^d}$ is the CAR-algebra, which we also call the quasi-local algebra. We define $P_0(\mathbb {Z}^d) := \{M\subseteq \mathbb {Z}^d~|~|M|<\infty \}$ and call

$$\begin{aligned} \mathcal {A}_0 := \bigcup _{M\in P_0(\mathbb {Z}^d)} \mathcal {A}_M \subseteq \mathcal {A}\end{aligned}$$

the local algebra, which is dense in $\mathcal {A}$ with respect to the norm topology. Consequently, an operator in $\mathcal {B}(\mathcal {F}(\mathbb {Z}^d,\mathbb {C}^n))$ is called quasi-local if it lies in $\mathcal {A}$ and local if it lies in $\mathcal {A}_0$. For each $\varphi \in \mathbb {R}$ there is a unique automorphism^3^$g_\varphi$ of $\mathcal {A}$, such that

$$\begin{aligned} g_\varphi (a^*_{x,i}) = \textrm{e}^{\textrm{i}\varphi } \, a^*_{x,i}, \quad \text {for all}~~ x\in \mathbb {Z}^d,~ i\in \{ 1,\dots ,n \} ~. \end{aligned}$$

One defines the set

$$\begin{aligned} \mathcal {A}^N := \{A\in \mathcal {A}~|~ \forall \varphi \in \mathbb {R}:\, g_\varphi (A) = A \} \end{aligned}$$

and calls $\mathcal {A}^N$ the gauge-invariant sub-algebra of $\mathcal {A}$. It is the closure of the set of all local observables that commute with the number operator, i.e. all local observables $A\in \mathcal {A}_0$ that satisfy $A\in \mathcal {A}_M \Rightarrow [A,N_M]:=[A,\sum _{x\in M}n_x]=0$. Its part in $M\subseteq \mathbb {Z}^d$ is denoted by $\mathcal {A}^N_M:= \mathcal {A}^N\cap \mathcal {A}_M$. For disjoint regions $M_1,M_2 \subseteq \mathbb {Z}^d$, $M_1\cap M_2 = \emptyset$, operators $A\in \mathcal {A}_{M_1}^N$ and $B\in \mathcal {A}_{M_2}$ commute: $[A,B] = 0$.

In order to define quantitative notions of localization for quasi-local operators, one makes use of the fact that one can localize operators to given regions by means of the fermionic conditional expectation. To this end first note that $\mathcal {A}$ has a unique state $\omega ^{\textrm{tr}}$ (commonly referred to as a tracial state) that satisfies

$$\begin{aligned} \omega ^{\textrm{tr}}(AB) = \omega ^{\textrm{tr}}(BA) \end{aligned}$$

for all $A,B \in \mathcal {A}$ (e.g. [2, Definition 4.1, Remark 2]).

#### Proposition 2.1

([2, Theorem 4.7]) For each $M\subseteq \mathbb {Z}^d$ there exists a unique linear map

$$\begin{aligned} \mathbb {E}_M:\mathcal {A}\rightarrow \mathcal {A}_M\,, \end{aligned}$$

called the conditional expectation with respect to $\omega ^\textrm{tr}$, such that

4 $$\begin{aligned} \forall A\in \mathcal {A}\; \;\forall B\in \mathcal {A}_M \,:\quad \omega ^{\textrm{tr}}(AB)=\omega ^{\textrm{tr}}(\mathbb {E}_M(A)B) \,. \end{aligned}$$

It is unital, positive and has the properties

$$\begin{aligned} \begin{aligned} \forall M\subseteq \mathbb {Z}^d\;\; \forall A,C\in \mathcal {A}_M\;\; \forall B\in \mathcal {A}\,&: \mathbb {E}_M ({A\,B\,C}) = A\, \mathbb {E}_M(B)\,C\\ \forall M_1,M_2 \subseteq \mathbb {Z}^d&: \mathbb {E}_{M_1} \circ \mathbb {E}_{M_2} = \mathbb {E}_{M_1\cap M_2}\,\\ \forall M \subseteq \mathbb {Z}^d&: \mathbb {E}_M \mathcal {A}^N \subseteq \mathcal {A}^N\\ \forall M \subseteq \mathbb {Z}^d \; \; \forall A \subseteq \mathcal {A}&: \Vert {\mathbb {E}_M(A)}\Vert \le \Vert {A}\Vert . \end{aligned} \end{aligned}$$

#### Proof

We note that there are two differences between our proposition and [2, Theorem 4.7]: As can be seen in the proof of the theorem, there is a unique linear map satisfying (4), and the property of being a conditional expectation is not required for the uniqueness. The statement $\forall M \subseteq \mathbb {Z}^d:$ $\mathbb {E}_M \mathcal {A}^N \subseteq \mathcal {A}^N$ is not part of [2, Theorem 4.7]; it however follows by the exact same reasoning as in [2, Corollary 4.8]. One just needs to replace the parity automorphism by $g_\varphi$ for $\varphi \in \mathbb {R}$. $\square$

With the help of $\mathbb {E}$ we can define subspaces of $\mathcal {A}^N$ that contain operators with well-defined decay properties (cf. [41] for similar definitions for quantum spin systems).

#### Definition 2.2

For $\nu \in \mathbb {N}_0$ and $A \in \mathcal {A}$ let

$$\begin{aligned} \Vert {A}\Vert _\nu := \Vert {A}\Vert + \sup _{k\in \mathbb {N}_0}( \Vert {A-\mathbb {E}_{\Lambda _k}A}\Vert (1+k)^\nu ) \end{aligned}$$

where $\Lambda _k = \{x\in \mathbb {Z}^d \, | \, \Vert {x}\Vert _\infty \le k \}$ is the box with side-length 2*k* around 0 ($\Vert {\cdot }\Vert _\infty$ is the maximum norm on $\mathbb {Z}^d$). We denote the set of all $A\in \mathcal {A}^N$ with finite $\Vert {\cdot }\Vert _\nu$ by $D_\nu$ and also define $D_{\infty } := \bigcap _{\nu \in \mathbb {N}_0} D_\nu$. We will sometimes refer to these norms as *decay norms*.

#### Lemma 2.3

Let $\nu \in \mathbb {N}_{0}$ and $A,B\in D_{\infty }$. Then

$$\begin{aligned} \Vert {AB}\Vert _\nu \le 2 \, \Vert {A}\Vert _\nu \, \Vert {B}\Vert _\nu \quad \text {and therefore} \quad \Vert {[A,B]}\Vert _{\nu } \le 4 \, \Vert {A}\Vert _\nu \, \Vert {B}\Vert _\nu ,\end{aligned}$$

and

$$\begin{aligned} \lim _{k\rightarrow \infty }\Vert {A - \mathbb {E}_{\Lambda _k}A}\Vert _{\nu } =0 \,. \end{aligned}$$

The relevant physical dynamics on $\mathcal {A}$ is generated by densely defined derivations, which in turn are constructed from so-called interactions. An *interaction* is a map $\varPhi : P_0(\mathbb {Z}^d) \rightarrow \mathcal {A}^N$, such that $\varPhi (\emptyset ) = 0$ and for all $M\in P_0(\mathbb {Z}^d)$ it holds that $\varPhi (M) \in \mathcal {A}_M$, $\varPhi (M)^* = \varPhi (M)$, and the sum

$$\begin{aligned} \sum _{\begin{array}{c} K\in P_0(\mathbb {Z}^d)\\ M\cap K \ne \emptyset \end{array}} \varPhi (K)\end{aligned}$$

converges unconditionally, meaning that the partial sums converge to the same value for all sequences that enumerate the index set. For two interactions $\varPhi$ and $\varPsi$ their commutator is given by

$$\begin{aligned} {[}\varPhi ,\varPsi ]: P_0(\mathbb {Z}^d) \rightarrow \mathcal {A}^N\,,\qquad M\mapsto [\varPhi ,\varPsi ](M) \;:= \sum _{\begin{array}{c} M_1,M_2 \subseteq M\\ M_1 \cup M_2= M \end{array}}[\varPhi (M_1),\varPsi (M_2)] \, . \end{aligned}$$

The map $\textrm{i}[\varPhi ,\varPsi ]$ satisfies the definition of an interaction except for the last requirement, which is not always satisfied. All the commutators of interactions appearing in the following will however be interactions (see Proposition 2.13).

Interactions define derivations on the algebra in the following way. For an interaction $\varPhi$ let

$$\begin{aligned} \mathcal {L}_{\varPhi }^\circ : \mathcal {A}_0 \rightarrow \mathcal {A},~ A\mapsto \sum _{M\in P_0(\mathbb {Z}^d)} [\varPhi (M),A] \, . \end{aligned}$$

It follows from [14, Propositions 3.1.15 and 3.2.22] that $\mathcal {L}_{\varPhi }^\circ$ is closable. We denote its closure by $\mathcal {L}_{\varPhi }$ and call it the Liouvillian of $\varPhi$. Some basic interactions that will appear frequently in the following are the number operator *N* and the position operators $X_j$ for $j\in \{1,\dots , d\}$, which are non-vanishing only on one-element sets. They are defined by $N(\{x\}) = n_x$ and $X_j(\{x\})= x_j n_x$ for $x\in \mathbb {Z}^d$.^4^

In the operator-algebraic framework, states are described as positive linear functionals $\omega :\mathcal {A}\rightarrow \mathbb {C}$ of norm 1. Given an interaction $\varPhi$, a state $\omega$ is called a $\mathcal {L}_{\varPhi }$-ground state, or simply ground state of $\varPhi$, if it holds that

$$\begin{aligned} \forall A \in D(\mathcal {L}_\varPhi ) : \quad \omega (A^* \mathcal {L}_\varPhi A) \ge 0 \, . \end{aligned}$$

Here, for a given linear operator *T* acting on $\mathcal {A}$, *D*(*T*) denotes its domain. For a state $\omega$ on $\mathcal {A}$ and an interaction $\varPhi$ we define

5 $$\begin{aligned} \overline{\omega }(\varPhi ) := \lim _{k\rightarrow \infty } \frac{1}{|\Lambda _k|}\sum _{M\subseteq \Lambda _k} \omega (\varPhi (M))\, , \end{aligned}$$

whenever the limit exists and call it the per-volume expectation value of $\varPhi$ with respect to $\omega$.

#### Definition 2.4

Let $\varPhi$ be an interaction and $\nu \in \mathbb {N}_{0}$. Let

$$\begin{aligned} \Vert \varPhi \Vert _{\nu } := \sup _{x\in \mathbb {Z}^d} \sum _{\begin{array}{c} M\in P_0(\mathbb {Z}^d)\\ x\in M \end{array}}(1+\textrm{diam}(M))^\nu \Vert \varPhi (M)\Vert ~. \end{aligned}$$

The set of all interactions with finite $\Vert {\cdot }\Vert _{\nu }$ is denoted by $B_{\nu }$. We also define $B_{\infty } := \bigcap _{\nu \in \mathbb {N}_0} B_{\nu }$.

For $$a>0$$ let

$$\begin{aligned} \Vert \varPhi \Vert _{\exp ,a} := \sup _{x\in \mathbb {Z}^d} \sum _{\begin{array}{c} M\in P_0(\mathbb {Z}^d)\\ x\in M \end{array}} \exp (a \, \textrm{diam}(M)) \Vert \varPhi (M)\Vert \end{aligned}$$

and denote the set of all interactions with finite $\Vert {\cdot }\Vert _{\exp ,a}$ for some $a>0$ by $B_{\exp }$. By $\textrm{diam}(M)$ we mean the maximal distance of two elements in *M* with respect to $\Vert {\cdot }\Vert _\infty$.

#### Lemma 2.5

For a $B_\infty$-interaction $\varPhi$, $j\in \{ 1,\dots , d \}$ and $p,q\in \mathbb {R}$ it holds that $D_\infty \subseteq D(\mathcal {L}_{p \varPhi + q X_j})$. Let $A\in D_\infty$, then the sums

$$\begin{aligned} \sum _{M\in P_0(\mathbb {Z}^d)} [\,\varPhi (M),A\,] \quad \text {and} \quad \sum _{x\in \mathbb {Z}^d} [\,x_j\,n_x,A\,]\end{aligned}$$

converge absolutely and

$$\begin{aligned} \mathcal {L}_{p \varPhi + q X_j}A = p \sum _{M\in P_0(\mathbb {Z}^d)} [\,\varPhi (M),A\,] + q \sum _{x\in \mathbb {Z}^d} [\,x_j\,n_x,A\,] \in D_\infty . \end{aligned}$$

For each $\nu \in \mathbb {N}_0$ there is a constant $c_\nu$, independent of $\varPhi , j, p, q, A$ such that

$$\begin{aligned} \Vert { \mathcal {L}_{p \varPhi + q X_j}A }\Vert _\nu \le c_\nu \, (p \,\Vert {\varPhi }\Vert _{d+1+2\nu } + q \,\Vert {n_0}\Vert ) \, \Vert {A}\Vert _{d+3+2\nu }.\end{aligned}$$

A cocycle of automorphisms is a family $(\alpha _{u,v})_{(u,v) \in \mathbb {R}^2}$ of automorphisms of $\mathcal {A}$ such that $\alpha _{u,u} = \textrm{id}$ for all $u\in \mathbb {R}$ and $\alpha _{u,v}\circ \alpha _{v,w} = \alpha _{u,w}$ for all $u,v,w\in \mathbb {R}$. We say a cocycle of automorphisms is generated by the family of interactions $(\varPhi ^{v})_{v\in \mathbb {R}}$ if for all $A \in \mathcal {A}_0$ it holds that $\partial _v \alpha _{u,v} A = \alpha _{u,v} \textrm{i}\mathcal {L}_{\varPhi ^{v}} A$. In the case where the generating family is a constant interaction $\varPhi$, the family $(\alpha _{0,v})_{v\in \mathbb {R}}$ is a one-parameter group of automorphisms with generator $\textrm{i}\,\mathcal {L}_{\varPhi }$. One can show that the family of all gauge automorphisms $(g_\varphi )_{ \varphi \in \mathbb {R}}$ is generated by the number operator *N* in this sense and that, for an observable $A\in D(\mathcal {L}_N)$, gauge-invariance ($A\in \mathcal {A}^N$) is equivalent to $\mathcal {L}_N A = 0$.

#### Lemma 2.6

If $(\varPhi ^{v})_{v\in \mathbb {R}}$ is a family of interactions such that $\sup _{v\in \mathbb {R}} \Vert {\varPhi ^{v}}\Vert _\nu < \infty$ for all $\nu \in \mathbb {N}_0$ and for each $M \in P_0(\mathbb {Z}^d)$ the map $v \mapsto \varPhi ^{v}(M)$ is continuous with respect to the norm topology in $\mathcal {A}$, then there is a unique cocycle of automorphisms $(\alpha _{u,v})_{(u,v) \in \mathbb {R}^2}$ generated by $(\varPhi ^{v})_{v\in \mathbb {R}}$. This cocycle has the property that for all $\nu \in \mathbb {N}_0$ there is a constant $c_\nu$ such that

$$\begin{aligned} \forall A\in D_\infty ,\, u,v\in \mathbb {R}:\quad \Vert {\alpha _{u,v} A }\Vert _\nu \le c_\nu \exp (c_\nu \, \sup _{t\in \mathbb {R}}\Vert {\varPhi ^t}\Vert _{2d+1+\nu } \, |u-v|) \Vert {A}\Vert _\nu \, . \end{aligned}$$

It also holds for all $u,v\in \mathbb {R}$ that $\alpha _{u,v} D_\infty = D_\infty$. If additionally $\sup _{v\in \mathbb {R}} \Vert {\varPhi ^{v}}\Vert _{\exp ,a} < \infty$ for some $a > 0$, then for all $\nu \in \mathbb {N}_0$ there exists a continuous function $b_\nu :\mathbb {R}\rightarrow \mathbb {R}$ that grows at most polynomially, such that

$$\begin{aligned} \Vert {\alpha _{u,v} A}\Vert _\nu \le b_\nu (|u-v|) \Vert {A}\Vert _\nu \quad \text { for all } A\in D_\infty ,\, u,v\in \mathbb {R}\,. \end{aligned}$$

### Periodic interactions

From now on we will specialise to periodic interactions and periodic states for which the existence of the per-volume expectation is easy to see. We also formulate additional properties of periodic interactions that are proved in Appendix B and will be used in the proof of our main theorems.

#### Definition 2.7

We denote by $\textrm{Aut}(\mathcal {A})$ the group of automorphisms on $\mathcal {A}$. A *translation* is a map $T:\mathbb {Z}^d \rightarrow \textrm{Aut}(\mathcal {A})$ that assigns to each possible shift vector $\gamma$ an automorphism $T_\gamma$ and satisfies the following properties:

(i) For all $\gamma \in \mathbb {Z}^d$ and $M \subseteq \mathbb {Z}^d$ it holds that $T_{\gamma }(\mathcal {A}_M) = \mathcal {A}_{M+\gamma }$.
(ii) For all $\gamma \in \mathbb {Z}^d$ and $x\in \mathbb {Z}^d$, it holds that $T_{\gamma }n_x = n_{x+\gamma }$.

Note that this definition of translations includes, in particular, so-called magnetic translations (see (9) below). The two properties of the definition guarantee that translations are compatible with the conditional expectation and gauge automorphisms respectively:

#### Lemma 2.8

Let *T* be a translation and $\gamma \in \mathbb {Z}^d$. It holds that

$$\begin{aligned} \forall M \subseteq \mathbb {Z}^d :&\quad T_\gamma \, \mathbb {E}_M = \mathbb {E}_{M + \gamma } \, T_\gamma \, ,\\ \forall \varphi \in \mathbb {R}:&\quad g_\varphi \, T_\gamma = T_\gamma \, g_\varphi \, . \end{aligned}$$

Let *T* be a translation. We say a state $\omega$ is *T*-periodic, if

$$\begin{aligned} \forall \, \gamma \in \mathbb {Z}^d, \, A\in \mathcal {A}:\quad \omega (T_\gamma A) = \omega (A)\, . \end{aligned}$$

An interaction $\varPhi$ is called *T*-periodic, if

$$\begin{aligned} \forall \, \gamma \in \mathbb {Z}^d, \, M\in P_0(\mathbb {Z}^d):\quad T_{\gamma } \varPhi (M) = \varPhi (M+\gamma )\,. \end{aligned}$$

#### Remark 2.9

Note that the definition of a translation does not require it be a group homomorphism. Translations do however satisfy a homomorphism property when acting on certain elements like the local number operators or terms of interactions that are periodic with respect to the translation.

In order to define a standard representation of a periodic interaction in terms of a quasi-local observable, we introduce the following notions. We say $M_1,M_2 \in P_0(\mathbb {Z}^d)$ have the same shape if there is a $\gamma \in \mathbb {Z}^d$ such that $M_1 = M_2 +\gamma$. For each $M\in P_0(\mathbb {Z}^d)$ we define $s(M) \in \mathbb {Z}^d$ as the vector that shifts the center of mass of *M* closest to 0. More precisely it is defined as the unique element of $\mathbb {Z}^d$ such that

6 $$\begin{aligned} \sum _{x\in \, M + s(M)} \frac{x}{|{M}|} \in {\big ( }-\tfrac{1}{2}\,,\,\tfrac{1}{2}\, {\big ]}^d \, . \end{aligned}$$

We call $M+s(M)$ the standard representative of *M* at 0 and denote the set of all standard representatives at 0 by $R_0(\mathbb {Z}^d)$. We call an interaction $\varPhi$ absolutely summable at 0 if

$$\begin{aligned} \sum \limits _{\begin{array}{c} M\in R_0(\mathbb {Z}^d) \end{array}} \varPhi (M) \end{aligned}$$

converges absolutely. In that case we denote the limit by $\varPhi _0$.

The following construction shows that, under an additional compatibility condition, adding up all the translates of a quasi-local observable yields a *T*-invariant interaction.

#### Definition 2.10

Let *T* be a translation. Then $A\in D_\infty$ is called *T-compatible*, iff $T_\gamma \, T_{\tilde{\gamma }} \,A = T_{\gamma +\tilde{\gamma }}\, A$ for all $\gamma , \tilde{\gamma } \in \mathbb {Z}^d$.

#### Proposition 2.11

Let *T* be a translation and $A\in D_\infty$ be self-adjoint and *T*-compatible. Then the map

$$\begin{aligned}&\varPhi _{A}^T:P_0(\mathbb {Z}^d) \rightarrow \mathcal {A}^N ,\; M\mapsto \varPhi _{A}^T(M)\\  &\quad :=\left\{ \begin{array}{cl} T_{\gamma }\mathbb {E}_{\Lambda _{0}}A &  \text{ if }\, M=\Lambda _0 +\gamma \, \text{ for } \text{ some }\, \gamma \in \mathbb {Z}^d\\ T_{\gamma }(\mathbb {E}_{\Lambda _{k}}A - \mathbb {E}_{\Lambda _{k-1}}A)&  \text{ if }\, M=\Lambda _k +\gamma \, \text{ for } \text{ some }\, \gamma \in \mathbb {Z}^d\, \text{ and }\, k\in \mathbb {N}\\ 0 &  \text{ otherwise } \end{array}\right. \end{aligned}$$

defines a *T*-periodic $B_\infty$-interaction with $(\varPhi _A^T)_0 = A$. Conversely, if $\varPhi$ is a *T*-periodic $B_\infty$-interaction, then it is absolutely summable at 0 and $\varPhi _0$ lies in $D_\infty$, is self-adjoint, and *T*-compatible.

We are now ready to state two technical propositions which will be used in the proof of the main theorem, but which are also relevant to its formulation.

#### Proposition 2.12

Let *T* be a translation, $\varPhi$ a *T*-periodic $B_{\infty }$-interaction, and $\omega$ a *T*-periodic state. It holds that:

(i) $$\mathcal {L}_{\varPhi }A = \sum _{\gamma \in \mathbb {Z}^d} [T_\gamma \varPhi _0, A]$$ for all $$A\in D_\infty $$.
(ii) $\overline{\omega }(\varPhi )$ exists and is equal to $\omega (\varPhi _0)$.

#### Proposition 2.13

Let $\varPhi$ and $\varPsi$ be $B_\infty$-interactions and $\omega$ a state such that $\varPhi$, $\varPsi$, and $\omega$ are all *T*-periodic with respect to a translation *T*. Moreover, let $j \in \{1,\dots ,d\}$, $p,q \in \mathbb {R}$.

(i) $\textrm{i}[p\varPhi + qX_j, \varPsi ]$ is a *T*-periodic $B_\infty$-interaction.
(ii) $$\textrm{i}[X_j,\varPsi ]_0 = \textrm{i}\mathcal {L}_{X_j}(\varPsi _0)$$.
(iii) $\mathcal {L}_{\textrm{i}[\varPsi ,p\varPhi +qX_j]}A = \sum _{\mu \in \mathbb {Z}^d} [\textrm{i}\, \mathcal {L}_\varPsi \, (p\, T_\mu \, \varPhi _0 + q \, \mu _j \, n_\mu ) ,\, A\, ]$ for all $A\in D_\infty$.
(iv) $$\omega (\textrm{i}[\varPhi ,\varPsi ]_0) =\omega (\textrm{i}\mathcal {L}_\varPhi (\varPsi _0)) =-\omega (\textrm{i}\mathcal {L}_\varPsi (\varPhi _0))$$.

## Model and Assumptions

We are now ready to define our model for a (topological) insulator perturbed by a constant electric field directly for the infinitely extended system using the formalism just described.

The unperturbed system is described by an interaction $H\in B_{\exp }$, which we call the Hamiltonian. We assume that *H* is periodic with respect to a translation *T* and that *H* has at least one gapped ground state $\omega _0$.

### Definition 3.1

(*cf.* [45, Definition 2.3]). Let $H\in B_{\exp }$. We say that a state $\omega _0$ is a *gapped ground state* for *H* with gap $g>0$, iff it holds for all $A\in \mathcal {A}_0$ that

7 $$\begin{aligned} \omega _0( A^* \mathcal {L}_H A ) \ge g \left( \omega _0(A^* A) - \left| \omega _0(A) \right| ^2 \right) \,. \end{aligned}$$

This condition implies that in the GNS representation induced by $\omega _0$ the GNS Hamiltonian representing $\mathcal {L}_H$ has a unique gapped ground state. For this reason states satisfying (7) are also called locally unique gapped ground states.

Systems with a gapped ground state are usually taken to characterize systems that are insulators at zero temperature. Our result (1) (compare (13) in Theorem 5.1) supports this view.

We are interested in how a system that is initially in a gapped ground state responds to the application of a small constant electric field and possibly also to a small bounded perturbation $V\in B_\infty$. Assuming that the system is initially in a ground state $\omega _0$, it is shown in [10, 28, 46] that when adiabatically switching on the perturbation $\varepsilon (V+X_1)$, where $V \in B_\infty$ is *T*-periodic, then the system evolves (up to errors of order $\mathcal {O}(\varepsilon ^{\infty })$) into a state $\omega _\varepsilon := \omega _0 \circ \beta _\varepsilon$ (called NEASS), where $\beta :\mathbb {R}\rightarrow \textrm{Aut}(\mathcal {A}),~ \varepsilon \mapsto \beta _{\varepsilon }$, is a continuous one-parameter family of quasi-locally generated automorphisms.

Our main results concern the currents present in such NEASS. Since our analysis does not depend on the way a NEASS is constructed or reached by adiabatic driving, we formulate the relevant properties of NEASS in a definition and refer exclusively to these properties in all main results. The existence of NEASS with these properties is dealt with in the Proposition 3.3.

### Definition 3.2

Let $H\in B_{\exp }$ be *T*-periodic, let $\omega _0$ be a *T*-periodic gapped ground state for *H*, and let $V\in B_\infty$ be *T*-periodic. We say that a family $[-1,1]\ni \varepsilon \mapsto \omega _\varepsilon = \omega _0 \circ \textrm{e}^{\textrm{i}\mathcal {L}_{S_{\varepsilon }}}$ is a *NEASS* for *H*, $\omega _0$, and $V+X_1$, iff $[-1,1]\ni \varepsilon \mapsto S_{\varepsilon }$ is a family of *T*-periodic interactions $S_{\varepsilon }\in B_\infty$ such that $\varepsilon \mapsto \frac{1}{\varepsilon }\Vert {S_\varepsilon }\Vert _\nu$ is bounded for all $\nu \in \mathbb {N}_0$ and

8 $$\begin{aligned} \sup _{A\in D_\infty \setminus \{0\} } \left( \frac{|\omega _{\varepsilon }(\mathcal {L}_{H_{\varepsilon }}A)|}{\Vert {A}\Vert _{d+3}}\right) = \mathcal {O}(\varepsilon ^{\infty }) \end{aligned}$$

for $$H_\varepsilon := H + \varepsilon (V+X_1)$$.

Equation (8) states that $\omega _\varepsilon \circ \mathcal {L}_{H_\varepsilon }$ is small in a sufficiently strong sense, and hence that $\omega _\varepsilon$ is almost invariant for the dynamics generated by $H_\varepsilon$ for long times.

Proposition 3.3 shows that for any gapped ground state $\omega _0$ there exists a corresponding NEASS $\omega _\varepsilon$. It is shown in [10] that the NEASS constructed in Proposition 3.3 agrees with that obtained by adiabatically switching on the perturbation. However, our results hold for any state $\omega _\varepsilon$ which is a NEASS for $\omega _0$ in the sense of Definition 3.2.

### Proposition 3.3

Let $H\in B_{\exp }$ and $V\in B_\infty$ be *T*-periodic. Then there exists a family $[-1,1]\ni \varepsilon \mapsto S_{\varepsilon }$ of *T*-periodic $B_\infty$-interactions $S_{\varepsilon }$ such that $\varepsilon \mapsto \frac{1}{\varepsilon }\Vert {S_\varepsilon }\Vert _\nu$ is bounded for all $\nu \in \mathbb {N}_0$ and such that for any *T*-periodic gapped ground state $\omega _0$ of *H* the state $\omega _\varepsilon := \omega _0 \circ \beta _\varepsilon$ with $\beta _{\varepsilon } := \textrm{e}^{\textrm{i}\mathcal {L}_{S_{\varepsilon }}}$ is a NEASS for *H*, $\omega _0$, and $V+X_1$.

Since the proof of Proposition 3.3 is merely a technical adaptation of the original one from [46], we postpone it to Appendix F. Note that for non-interacting continuum systems the NEASS with similar properties was constructed in [37] with predecessors going back to [42, 44].

Let us briefly discuss a relevant example of a system (in $d=2$ spatial dimensions and with $n=1$ degrees of freedom per unit cell) for which one can prove the existence of gapped ground states, namely the so-called Hofstadter model with small interactions. The one-body Hamiltonian of the non-interacting Hofstadter model is just the magnetic discrete Laplacian (discrete Landau operator) $\mathfrak {h}_0^{b}$, i.e. the operator on the lattice $\mathbb {Z}^2$ with integral kernel

$$\begin{aligned} \mathfrak {h}_0^{b}(x,y):= \left\{ \begin{array}{cl} 1 & \text{ if } x_1=y_1 \text{ and } |x_2-y_2|=1\\ \textrm{e}^{\textrm{i}bx_2(x_1-y_1)} & \text{ if } x_2=y_2 \text{ and } |x_1-y_1|=1\\ 0& \text{ otherwise. } \end{array}\right. \end{aligned}$$

Here $b\in \mathbb {R}$ is the strength of a constant external magnetic field. Note that $\mathfrak {h}_0^b\in \mathcal {B}( \ell ^2(\mathbb {Z}^2))$ is invariant with respect to magnetic translations $t^b:\mathbb {Z}^2\rightarrow \mathcal {B}( \ell ^2(\mathbb {Z}^2))$, $\gamma \mapsto t^b_\gamma$, with $(t^b_\gamma f)(x):= \textrm{e}^{ \textrm{i}b x_1 \gamma _2} f(x+\gamma )$, i.e. for all $\gamma \in \mathbb {Z}^2$ it holds that $t^b_\gamma \mathfrak {h}_0^b (t^b_\gamma )^{-1}= \mathfrak {h}_0^b$. The second quantization of $\mathfrak {h}^b_0$ reads

$$\begin{aligned} H_0^{b,\mu }:= \sum _{\begin{array}{c} x,y\in \mathbb {Z}^2,\\ \Vert x-y\Vert _{ 1}=1 \end{array}} \textrm{e}^{ \textrm{i}\frac{x_2+y_2}{2} b (x_1-y_1)} a_x^* a_y - \mu \sum _{x\in \mathbb {Z}^2} n_x, \end{aligned}$$

where $\mu \in \mathbb {R}$ is the chemical potential. The unitary magnetic translations $t^b$ lift to magnetic translations $T^b$ given by the family of automorphisms defined by uniquely extending for each $\gamma \in \mathbb {Z}^2$ the map

9 $$\begin{aligned} a_y\mapsto T^b_\gamma (a_y) := \textrm{e}^{-\textrm{i}b y_1 \gamma _2} a_{y+\gamma } \,,\quad y\in \mathbb {Z}^2\,, \end{aligned}$$

to a map $T^b_\gamma \in \textrm{Aut}(\mathcal {A})$. It is straightforward to check that $\gamma \mapsto T^b_\gamma$ defines a translation in the sense of Definition 2.7 and that $H_0^{b,\mu }$ is translation invariant with respect to $T^b$. The latter follows from the properties of second quantization, but can also be verified directly: let $h_0^\mu :=a_{(1,0)}^* a_{(0,0)} + a_{(0,1)}^* a_{(0,0)} +a_{(0,0)}^*a_{(1,0)} + a_{(0,0)}^*a_{(0,1)}- \mu n_{(0,0)}$ and observe that $H_0^{b,\mu } = \sum _{\gamma \in \mathbb {Z}^2} T_\gamma ^b \,h_0$. Since $T^b_{\tilde{\gamma }}(T^b_\gamma (a_y))= \textrm{e}^{-\textrm{i} b \gamma _1 \tilde{\gamma }_2} T^b_{\tilde{\gamma }+\gamma }(a_y)$ we have $T^b_{\tilde{\gamma }}(T^b_\gamma (h_0^\mu ))= T^b_{\tilde{\gamma }+\gamma }(h_0^\mu )$, thus making the $T^b$-invariance explicit. The spectrum of the one-body Hofstadter Hamiltonian $\mathfrak {h}_0^{b}$ is very well understood [30] and, in particular, it is known that the set of values $(b,\mu )\in \mathbb {R}^2$ for which $\mu$ lies in a gap of $\mathfrak {h}_0^{b}$, i.e. $\mu \notin \sigma (\mathfrak {h}_0^{b})$, is open, nonempty and actually of full Lebesgue measure (see [3] and, e.g., the discussion in [43]).

The following proposition states that for such values of $(b,\mu )$ the many-body Hofstadter interaction $H_0^{b,\mu }$ satisfies the gap condition (7) and that, moreover, this property persists when adding sufficiently weak local many-body interactions to $H_0^{b,\mu }$, e.g. a nearest neighbor two-body interaction

$$\begin{aligned} V:= \sum _{\begin{array}{c} x,y\in \mathbb {Z}^2,\\ \Vert x-y\Vert _{1}=1 \end{array}} n_xn_y. \end{aligned}$$

### Proposition 3.4

Let $b,\mu \in \mathbb {R}$ such that $\mu \notin \sigma (\mathfrak {h}_0^{b})$ and let $V\in B_{\exp }$ be $T^b$-periodic. Then there exists $\lambda _0>0$ such that for all $\lambda \in (-\lambda _0,\lambda _0)$ the *weakly interacting Hofstadter Hamiltonian*

$$\begin{aligned} H^{b,\mu }_{\lambda }:= H_0^{b,\mu } + \lambda V \end{aligned}$$

has a $T^b$-periodic gapped ground state in the sense of Definition 3.1.

The proof of Proposition 3.4, which is postponed to Appendix G, relies on known results [16, 23, 34] on the stability of the spectral gap for free Fermion systems.

## The Off-Diagonal Map

Before we can formulate our main results, we need to introduce a further technical ingredient, namely the map that selects the off-diagonal part of an operator or of an interaction with respect to a ground state $\omega _0$. This map was introduced in [9, 26] for finite gapped systems and extended in [41] to infinite systems with a gap in the bulk. Here we further generalize it by composing it with automorphisms of the algebra $\mathcal {A}$. Its definition involves the choice of a function $W_g: \mathbb {R}\rightarrow \mathbb {R}$ having the following properties for some $g>0$:

(i) $$W_g$$ is odd;
(ii) the Fourier transform ${\widehat{W}}_g$ of $W_g$ satisfies $\widehat{W_g}(k) = \frac{- \textrm{i}}{\sqrt{2 \pi }\, k}$ for $k \in \mathbb {R}\setminus [-g,g]$;
(iii) $\sup _{s\in \mathbb {R}} \bigl ( |s |^n |W_g(s) |\bigr ) < \infty$ for all $n\in \mathbb {N}_0$.

It is easy to see that such a function exists for any $g>0$, and an explicit example with additional properties is given in [9, Lemma 2.6] (see also [9, Lemma 2.3 and Equation 2.12]).

The following terminology and notation will be useful.

### Definition 4.1

We say that a tuple $({\omega _0,H,V,W_g,T,({S_\varepsilon })_{\varepsilon \in [-1,1]}})$ is a *gapped periodic system* with perturbation $\varepsilon (V+X_1)$ if *T* is a translation, $H\in B_{\exp }$ and $V\in B_\infty$ are *T*-periodic, $\omega _0$ is a gapped ground state of *H* with gap $g>0$, the map $W_g$ satisfies the above properties (i)–(iii), and $({S_\varepsilon })_{\varepsilon \in [-1,1]}$ is a family of *T*-periodic interactions having the properties of the one constructed in Proposition 3.3.

Whenever a gapped periodic system $({\omega _0,H,V,W_g,T,({S_\varepsilon })_{\varepsilon \in [-1,1]}})$ is given, then $\omega _\varepsilon := \omega _0\circ \beta _\varepsilon$ with $\beta _\varepsilon :=\textrm{e}^{\textrm{i}\mathcal {L}_{S_\varepsilon }}$ denotes the corresponding NEASS, and $h_0 {:}{=}\sum _{M\in R_0(\mathbb {Z}^d)} H(M)$ the part of *H* located in the origin. Finally, for a general automorphism $\alpha$ of $\mathcal {A}$ we write $\omega _\alpha := \omega _0 \circ \alpha$.

Proposition 3.3 shows that, given a *T*-periodic Hamiltonian with a gapped ground state $\omega _0$, then for every *T*-periodic $V\in B_\infty$ there exists a gapped periodic system $({\omega _0,H,V,W_g,T,({S_\varepsilon })_{\varepsilon \in [-1,1]}})$ for the given data.

The reason for introducing this terminology is that, in general, *H* does not uniquely determine either $\omega _0$, or $W_g$, or *T*, or $({S_\varepsilon })$. Since several of the following definitions and constructions depend not only on *H*, $\omega _0$, and *V*, but also on the choices of $W_g$, *T* and $({S_\varepsilon })$, we make this dependence explicit. However, the final results do not depend on these choices, which we will also make explicit. Actually, it turns out that the Hall conductivity does not even depend on *H* and *V*, but only on $\omega _0$.

We can now define two more maps that will be relevant for the formulation and the proofs of our results, namely the off-diagonal map and the quasi-local inverse of the Liouvillian.

### Lemma 4.2

Let $({\omega _0,H,V,W_g,T,({S_\varepsilon })_{\varepsilon \in [-1,1]}})$ be a gapped periodic system, let $\alpha \in \textrm{Aut}(\mathcal {A})$ be such that $\alpha D_\infty = D_\infty$ and $\alpha T_\gamma = T_\gamma \alpha$ for all $\gamma \in \mathbb {Z}^d$, and $\varPsi$ an interaction of the form $\varPsi = p\, Y + q\, X_j$, where $p,q \in \mathbb {R}$, $j\in \{1,\dots ,d\}$ and $Y \in B_\infty$ is *T*-periodic. Then the operators

$$\begin{aligned} (\varPsi ^{\textrm{OD}\alpha })_* := \alpha ^{-1} \int _{\mathbb {R}}\textrm{d}s \, W_g(s) \, \textrm{e}^{\textrm{i}s\mathcal {L}_{H}} \, \alpha \, \textrm{i}\,\mathcal {L}_\varPsi \, \alpha ^{-1} \, h_0 \end{aligned}$$

and

$$\begin{aligned} \mathcal {I}(\varPsi )_* {:}{=}- \int _{\mathbb {R}}\textrm{d}s \,W_g(s) \int _{0}^s \textrm{d}u \, \textrm{e}^{\textrm{i}u\mathcal {L}_{H}} \, \textrm{i}\, \mathcal {L}_\varPsi \, h_0 \, , \end{aligned}$$

defined via Bochner integrals in $\mathcal {A}$, are in $D_\infty$, self-adjoint, and *T*-compatible.

The proofs of the lemmas presented in this section are provided in Appendix C.

### Definition 4.3

Given the assumptions of Lemma 4.2, we define the *T*-periodic $B_\infty$-interactions

10 $$\begin{aligned} \varPsi ^{\textrm{OD}\alpha } {:}{=}\varPhi _{(\varPsi ^{\textrm{OD}\alpha })_*}^T\quad \text{ and }\quad \mathcal {I}(\varPsi ) {:}{=}\varPhi _{\mathcal {I}(\varPsi )_*}^T\, \end{aligned}$$

using Proposition 2.11. For $\alpha =\beta _{\varepsilon }=\textrm{e}^{\textrm{i}\mathcal {L}_{S_{\varepsilon }}}$, we denote $\varPsi ^{^{\textrm{OD}\alpha }}$ simply by $\varPsi ^{^{\textrm{OD}\varepsilon }}$, and for $\alpha =\textrm{id}$, by $\varPsi ^{^{\textrm{OD}}}$. Note that the interactions $\varPsi ^{\textrm{OD}\alpha }$ and $\mathcal {I}(\varPsi )$ are defined in such a way that $(\varPsi ^{\textrm{OD}\alpha })_0 = (\varPsi ^{\textrm{OD}\alpha })_*$ and $\mathcal {I}(\varPsi )_0 = \mathcal {I}(\varPsi )_*$. From now on, we will always refer to these quasi-local operators as $(\varPsi ^{\textrm{OD}\alpha })_0$ and $\mathcal {I}(\varPsi )_0$.

We call the map $\varPsi \mapsto \varPsi ^{\textrm{OD}\alpha }$ the off-diagonal map, and refer to $\varPsi ^{\textrm{OD}\alpha }$ as the off-diagonal part of $\varPsi$ (the dependence on $\alpha$ is left implicit if no ambiguity can arise).

### Lemma 4.4

Let $({\omega _0,H,V,W_g,T,({S_\varepsilon })_{\varepsilon \in [-1,1]}})$ be a gapped periodic system, let $\alpha \in \textrm{Aut}(\mathcal {A})$ be such that $\alpha D_\infty = D_\infty$ and $\alpha T_\gamma = T_\gamma \alpha$ for all $\gamma \in \mathbb {Z}^d$, and let $\varPsi$ be an interaction of the form $\varPsi = p\, Y+ q\, X_j$, where $p,q \in \mathbb {R}$, $j\in \{1,\dots ,d\}$ and $Y\in B_\infty$ is *T*-periodic. Then for all $A\in D_\infty$

11 $$\begin{aligned} \omega _{\alpha }(\mathcal {L}_{\varPsi } A) = \omega _\alpha (\mathcal {L}_{\varPsi ^{\textrm{OD}\alpha } } A) \end{aligned}$$

and

12 $$\begin{aligned} \mathcal {L}_{\textrm{i}[\mathcal {I}(\varPsi ), H]} A = \mathcal {L}_{\varPsi ^{\textrm{OD}}} A\,. \end{aligned}$$

### Lemma 4.5

Let $({\omega _0,H,V,W_g,T,({S_\varepsilon })_{\varepsilon \in [-1,1]}})$ be a gapped periodic system. For $j \in \{1,\dots ,d\}$ and $\nu \in \mathbb {N}_0$ the map $[-1,1] \rightarrow \mathbb {R}, ~ \varepsilon \mapsto \Vert {(X_j^{\textrm{OD}\varepsilon })_0}\Vert _\nu$ is bounded.

### Remark 4.6

The terminology “off-diagonal part” is motivated by the analogue of Equation (11) for pure states in Hilbert spaces: Assume that $P_\alpha$ is a finite-rank orthogonal projection and $A,\varPsi$ are bounded operators on a Hilbert space. Then with $\varPsi ^{\textrm{OD}\alpha }:=P_\alpha \varPsi P_\alpha ^\perp +P_\alpha ^\perp \varPsi P_\alpha$ and $\varPsi ^{\textrm{D}_\alpha }:=P_\alpha \varPsi P_\alpha +P_\alpha ^\perp \varPsi P_\alpha ^\perp$

$$\begin{aligned}  &   \omega _\alpha (\mathcal {L}_\varPsi A) := \textrm{tr}(P_\alpha [\varPsi ,A])\\  &   \quad = \textrm{tr}(P_\alpha [\varPsi ^{\textrm{OD}\alpha },A]) + \textrm{tr}(P_\alpha [\varPsi ^{\textrm{D}_\alpha },A]) = \textrm{tr}(P_\alpha [\varPsi ^{\textrm{OD}\alpha },A]) =: \omega _\alpha (\mathcal {L}_{\varPsi ^{\textrm{OD}\alpha }} A), \end{aligned}$$

since

$$\begin{aligned}  &   \textrm{tr}(P_\alpha [\varPsi ^{\textrm{D}_\alpha },A]) \\  &   \quad = \textrm{tr}(P_\alpha [P_\alpha \varPsi P_\alpha +P_\alpha ^\perp \varPsi P_\alpha ^\perp ,A])= \textrm{tr}(P_\alpha \varPsi P_\alpha A P_\alpha ) -\textrm{tr}(P_\alpha A P_\alpha \varPsi P_\alpha ) =0 \end{aligned}$$

by cyclicity of the trace. On the other hand, Equation (12) implies that $-\textrm{i}\mathcal {I}$ inverts the action of the Liouvillian $[H,\cdot ]$ on $\varPsi$ inside $\omega _0$ in the sense that for all $A\in D_\infty$

$$\begin{aligned} \omega _0(\mathcal {L}_{-\textrm{i}\mathcal {I}([H,\varPsi ])}A)=\omega _0(\mathcal {L}_\varPsi A) . \end{aligned}$$

This, together with the fact that $\mathcal {I}$ maps $B_\infty$-interactions to $B_\infty$-interactions, motivates the name “quasi-local inverse of the Liouvillian” for the map $\mathcal {I}$, see also [40, Appendix D] and references therein.

## Main Results

Our main result states that the longitudinal current density in the NEASS $\omega _\varepsilon$ is asymptotically smaller than any power of $\varepsilon$, where physically $\varepsilon$ corresponds to the strength of the applied constant electric field. The transversal or Hall current density is linear in the applied field up to errors that are again asymptotically smaller than any power of $\varepsilon$. The proportionality factor in this linear relation is, by definition, the Hall conductivity. We obtain an explicit formula for the Hall conductivity that depends only on $\omega _0$, but neither on *H* nor on the bounded part *V* of the perturbation.

The current density in the direction $j\in \{1,\ldots ,d\}$ is defined as the expectation per unit volume of the current operator

$$\begin{aligned} J_j:= \textrm{i} [H_\varepsilon ,X_j] = \textrm{i} [H+\varepsilon V,X_j] \in B_\infty \end{aligned}$$

associated with the perturbed Hamiltonian $H_\varepsilon = H+\varepsilon (V+X_1)$ evaluated in the NEASS $\omega _\varepsilon$.

### Theorem 5.1

Let $({\omega _0,H,V,W_g,T,({S_\varepsilon })_{\varepsilon \in [-1,1]}})$ be a gapped periodic system (see Definition 4.1). Then

13 $$\begin{aligned} \overline{\omega _{\varepsilon }}(J_1) = \mathcal {O}(\varepsilon ^{\infty }) \,, \end{aligned}$$

and, for $j\in \mathbb {N}$ with $1<j \le d$,

14 $$\begin{aligned} \overline{\omega _{\varepsilon }}(J_j) = -\varepsilon \,\overline{\omega _0}(\textrm{i}[X_1^{\textrm{OD}},X_j^{\textrm{OD}}]) + \mathcal {O}(\varepsilon ^{\infty }) ~. \end{aligned}$$

The Hall conductivity $\sigma ^{\textrm{H}}_{j1} := -\,\overline{\omega _0}(\textrm{i}[X_1^{\textrm{OD}},X_j^{\textrm{OD}}])$ depends only on $\omega _0$, i.e. it agrees for all gapped periodic systems having $\omega _0$ as ground state.

### Proof

Let $j\in \{ 1,\dots ,d \}$. We first note that $J_j=\textrm{i}[H+\varepsilon V,X_j]$ is a *T*-periodic $B_\infty$-interaction with $\textrm{i}[H+\varepsilon V,X_j]_0 = -\textrm{i}\mathcal {L}_{X_j}(h_0+\varepsilon (V)_0)$ (see Proposition 2.13). Thus, with Lemma 4.4 and Propositions 2.13 and 2.12 we get

$$\begin{aligned} \overline{\omega _{\varepsilon }}(\textrm{i}[H+\varepsilon V,X_j])&= -\textrm{i}\,\omega _\varepsilon (\mathcal {L}_{X_j}(h_0+\varepsilon (V)_0))\\  &= -\textrm{i}\,\omega _\varepsilon (\mathcal {L}_{X_j^{\textrm{OD}\varepsilon }}(h_0+\varepsilon (V)_0)) = \textrm{i}\,\omega _\varepsilon (\mathcal {L}_{H+\varepsilon V} (X_j^{\textrm{OD}\varepsilon })_0) ~. \end{aligned}$$

By substituting $H+\varepsilon V = H_\varepsilon - \varepsilon X_1$ we obtain

$$\begin{aligned} \overline{\omega _{\varepsilon }}(\textrm{i}[H+\varepsilon V,X_j])&= \textrm{i}\,\omega _\varepsilon (\mathcal {L}_{H_\varepsilon } (X_j^{\textrm{OD}\varepsilon })_0) - \textrm{i}\,\varepsilon \, \omega _\varepsilon (\mathcal {L}_{X_1} (X_j^{\textrm{OD}\varepsilon })_0) ~. \end{aligned}$$

The bound

$$\begin{aligned} |\omega _\varepsilon (\mathcal {L}_{H_\varepsilon } (X_j^{\textrm{OD}\varepsilon })_0)| \le \Vert {(X_j^{\textrm{OD}\varepsilon })_0}\Vert _{3d+1} \sup _{A\in D_\infty \setminus \{0\} } \left\{ \frac{|\omega _{\varepsilon }(\mathcal {L}_{H_{\varepsilon }}A)|}{\Vert {A}\Vert _{3d+1}}\right\} ~, \end{aligned}$$

together with the almost stationarity of the NEASS (Proposition 3.3) and Lemma 4.5 yields

$$\begin{aligned} \overline{\omega _{\varepsilon }}(J_j)&= - \textrm{i}\, \varepsilon \, \omega _\varepsilon (\mathcal {L}_{X_1} (X_j^{\textrm{OD}\varepsilon })_0) + \mathcal {O}(\varepsilon ^{\infty }) ~. \end{aligned}$$

Using Lemma 4.4 and Proposition 2.13 once more, we obtain

$$\begin{aligned} \overline{\omega _{\varepsilon }}(J_j) = - \textrm{i}\,\varepsilon \, \omega _\varepsilon (\mathcal {L}_{X_1^{\textrm{OD}\varepsilon }} (X_j^{\textrm{OD}\varepsilon })_0) + \mathcal {O}(\varepsilon ^{\infty }) = - \varepsilon \,\overline{\omega _\varepsilon }(\textrm{i}[X_1^{\textrm{OD}\varepsilon }, X_j^{\textrm{OD}\varepsilon }]) + \mathcal {O}(\varepsilon ^{\infty }) ~. \end{aligned}$$

For $j=1$ we thus obtain (13). To obtain (14) for $1<j \le d$, it remains to show that $\overline{\omega _\varepsilon }(\textrm{i}[X_1^{\textrm{OD}\varepsilon }, X_j^{\textrm{OD}\varepsilon }])$ is independent of $\varepsilon$, or, more generally, that expressions of this form do not change along continuous families of locally generated automorphisms of the algebra. In the non-interacting case this statement is known as the Chern–Simons lemma [32, 37] and we prove an analogous result in our setting in Lemma 5.2. With its help we obtain the final result

$$\begin{aligned} \overline{\omega _{\varepsilon }}(J_j)&= - \varepsilon \,\overline{\omega _0}( \textrm{i}[X_1^{\textrm{OD}}, X_j^{\textrm{OD}}]) + \mathcal {O}(\varepsilon ^{\infty }) ~. \end{aligned}$$

For the last claim let $({\omega _0,H_1,V_1,W_1,T_1,({S_{1,\varepsilon }})_{\varepsilon \in [-1,1]}})$ and $({\omega _0,H_2,V_2,W_2, T_2,({S_{2,\varepsilon }})_{\varepsilon \in [-1,1]}})$ both be gapped periodic systems with the same ground state $\omega _0$ and let $(X_j)^{\textrm{OD}1}$ and $(X_j)^{\textrm{OD}2}$ for $j\in \{ 2,\dots , d \}$ be the respective off-diagonal position operators. Since both off-diagonal maps satisfy the Property (11) from Lemma 4.4 we can calculate with Proposition 2.13

$$\begin{aligned} \begin{aligned} \omega _0(\mathcal {L}_{X_1^{\text {OD}1}}(X_j^{\text {OD}1})_0)&= \omega _0(\mathcal {L}_{X_1^{\text {OD}2}}(X_j^{\text {OD}1})_0) = -\omega _0(\mathcal {L}_{X_j^{\text {OD}1}}(X_1^{\text {OD}2})_0) = -\omega _0(\mathcal {L}_{X_j^{\text {OD}2}}(X_1^{\text {OD}2})_0) \\  &=\omega _0(\mathcal {L}_{X_1^{\text {OD}2}}(X_j^{\text {OD}2})_0) \end{aligned} \end{aligned}$$

and therefore, the Hall conductivities are the same. $\square$

The proof of the following lemma, the CAR-algebra version of the Chern–Simons lemma, stating the invariance of the Hall conductivity under locally generated automorphisms is given in Appendix D.

### Lemma 5.2

Let $({\omega _0,H,V,W_g,T,({S_\varepsilon })_{\varepsilon \in [-1,1]}})$ be a gapped periodic system and let $(\alpha _{u,v})_{(u,v) \in \mathbb {R}^2}$ be a cocycle of automorphisms generated by a family of *T*-periodic interactions $(\varPhi ^{v})_{v\in \mathbb {R}}$, such that $\sup _{v\in \mathbb {R}} \Vert {\varPhi ^{v}}\Vert _\nu < \infty$ for all $\nu \in \mathbb {N}_0$ and for each $M \in P_0(\mathbb {Z}^2)$ the map $v \mapsto \varPhi ^{v}(M)$ is continuous. For all $u,v\in \mathbb {R}$ and $j\in \{1,\dots ,d\}$ it holds that

$$\begin{aligned} \overline{\omega _{\alpha _{u,v}}}(\textrm{i}[ X_1^{\textrm{OD}\alpha _{u,v}},X_j^{\textrm{OD}\alpha _{u,v}}]) = \overline{\omega _{0}}(\textrm{i}[X_1^{\textrm{OD}},X_j^{\textrm{OD}}])~. \end{aligned}$$

Given a translation *T*, we say that a state $\omega$ is *T*-gapped if there exist *H*, *V*, $W_g$, and $({S_\varepsilon })_{\varepsilon \in [-1,1]}$, such that $({\omega ,H,V,W_g,T,({S_\varepsilon })_{\varepsilon \in [-1,1]}})$ is a gapped periodic system. Two *T*-periodic states $\omega _1$ and $\omega _2$ are said to lie in the same *T*-periodic phase if there is a cocycle of automorphisms $(\alpha _{u,v})_{(u,v) \in \mathbb {R}^2}$ which satisfies $\omega _2 = \omega _1\circ \alpha _{0,1}$ and is generated by a family of *T*-periodic interactions $(\varPhi ^{v})_{v\in \mathbb {R}}$ such that $\sup _{v\in \mathbb {R}} \Vert {\varPhi ^{v}}\Vert _\nu < \infty$ for all $\nu \in \mathbb {N}_0$ and for each $M \in P_0(\mathbb {Z}^d)$ the map $v \mapsto \varPhi ^{v}(M)$ is continuous.

### Corollary 5.3

*T*-gapped states that lie in the same *T*-periodic phase with respect to some translation *T* have the same Hall conductivities.

### Proof

Let $({\omega _1,H_1,V_1,W_1,T,({S_{1,\varepsilon }})_{\varepsilon \in [-1,1]}})$ and $({\omega _2,H_2,V_2,W_2,T,({S_{2,\varepsilon }})_{\varepsilon \in [-1,1]}})$ be two gapped periodic systems corresponding to two *T*-gapped states $\omega _1$ and $\omega _2$ in the same *T*-periodic phase. We denote the off-diagonal maps associated to each system by $(\cdot )^{\textrm{OD}1}$ and $(\cdot )^{\textrm{OD}2}$ and the automorphism connecting the states by $\alpha$. The *j*-th Hall conductivity of $\omega _2$ is given by $\omega _2(\mathcal {L}_{X_1^{\textrm{OD}2}} (X_j^{\textrm{OD}2})_0)$. The off-diagonal property with respect to $\omega _2 = \omega _1 \circ \alpha$ is satisfied by both $(\cdot )^{\textrm{OD}2}$ and $(\cdot )^{\textrm{OD}1\alpha }$ (see Lemma 4.4). We can therefore use Proposition 2.13 as in the last part of the proof of Theorem 5.1 to arrive at

$$\begin{aligned} \omega _2(\mathcal {L}_{X_1^{\textrm{OD}2}} (X_j^{\textrm{OD}2})_0) =\omega _2(\mathcal {L}_{X_1^{\textrm{OD}1\alpha }} (X_j^{\textrm{OD}1\alpha })_0)\,. \end{aligned}$$

Lemma 5.2 tells us that this expression is equal to $\omega _1(\mathcal {L}_{X_1^{\textrm{OD}1}} (X_j^{\textrm{OD}1})_0)$, which is the *j*-th Hall conductivity of $\omega _1$. $\square$

### Corollary 5.4

Let $d=2$ and let $({\omega _0,H,V,W_g,T,({S_\varepsilon })_{\varepsilon \in [-1,1]}})$ be a gapped periodic system. If $\omega _0$ is invertible (see Appendix E, Definition E.2), then the Hall conductivity of all states in the same *T*-periodic phase as $\omega _0$ lies in $\frac{1}{2\pi } \mathbb {Z}$.

### Proof

Proposition E.3 shows that the Hall conductivity of $\omega _0$ is equal to a certain ground-state expectation $\omega _0(\textrm{i}[K_{\varSigma _1,\varSigma _1^{\textsf{C}}}, K_{\varSigma _2,\varSigma _2^{\textsf{C}}}])$. In [31] it is shown that, if $\omega _0$ is invertible, this quantity lies in $\frac{1}{2\pi } \mathbb {Z}$ (the relevant statements can be found in [31, Theorem 1] and [31, Equation 55]). By Corollary 5.3 the Hall conductivity is constant within the *T*-periodic phase of $\omega _0$. $\square$

Our final corollary shows that the macroscopic Hall conductance has zero variance. Let $d=2$ and let

$$\begin{aligned} J_L:= \sum _{m=1}^L j_{m,2}:= \sum _{m=1}^L T_{(m,0)}\textrm{i}[H+\varepsilon V,X_2]_0 \end{aligned}$$

denote the Hall current operator through a line of length *L*. Then the Hall conductance observable

$$\begin{aligned} G^{\textrm{H}}_{21,L}:= \frac{J_L}{\varepsilon L} \end{aligned}$$

is the ratio of the current flowing through a line of length *L* to the voltage drop along that line, where the latter is equal to the electric field strength $\varepsilon$ times the length *L* of the line in our setup.

### Corollary 5.5

Let $d=2$ and let $({\omega _0,H,V,W_g,T,({S_\varepsilon })_{\varepsilon \in [-1,1]}})$ be a gapped periodic system. It holds for all $L>0$ that

15 $$\begin{aligned} \omega _\varepsilon (G^{\textrm{H}}_{21,L} ) = \sigma _{21}^\textrm{H} + \mathcal {O}\left( \varepsilon ^\infty \right) \end{aligned}$$

and

$$\begin{aligned} \textrm{var}\hspace{-2pt}\left( G^{\textrm{H}}_{21,L}\right) := \omega _\varepsilon \hspace{-2pt}\left( \left( G^{\textrm{H}}_{21,L}\right) ^2\right) -\left( \omega _\varepsilon \hspace{-2pt}\left( G^{\textrm{H}}_{21,L}\right) \right) ^2=\mathcal {O}\left( \frac{1}{\varepsilon ^2 L}\right) . \end{aligned}$$

### Proof

The first statement is an immediate consequence of translation invariance and Theorem 5.1,

$$\begin{aligned} \omega _\varepsilon (J_L) =\sum _{m=1}^L \omega _\varepsilon (j_{m}) = \sum _{m=1}^L \omega _\varepsilon \hspace{-2pt} \left( T_{(m,0)} j_{0}\right) = L \, \omega _\varepsilon ( j_{0} ) = L \,\overline{\omega }_\varepsilon (\textrm{i}[H+\varepsilon V,X_2]), \end{aligned}$$

where here and in the rest of the proof we write $j_m:=j_{m,2}$.

The second statement follows from exponential decay of correlations in the ground state $\omega _0$, which was established in [24]. To see this, first note that

$$\begin{aligned} \omega _\varepsilon (J_L^2) - \omega _\varepsilon (J_L)^2= &   \sum _{m,\ell =1}^L \big (\omega _\varepsilon \left( j_{m}\,j_{\ell } \right) - \omega _\varepsilon \left( j_{m} \right) \omega _\varepsilon \left( j_{\ell } \right) \big )\\= &   \sum _{m,\ell =1}^L \big (\omega _0\left( \beta _\varepsilon (j_{m})\beta _\varepsilon ( j_{\ell }) \right) - \omega _0\left( \beta _\varepsilon (j_{m} ) \right) \omega _0\left( \beta _\varepsilon (j_{\ell }) \right) \big ). \end{aligned}$$

Since $\beta _\varepsilon (j_{0})\in D_\infty$, it holds for every $\nu \ge 0$ and $k>0$ that $\Vert (1- \mathbb {E}_{\Lambda _k})\beta _\varepsilon (j_{0})\Vert \le \Vert \beta _\varepsilon (j_{0})\Vert _\nu \,(1+k)^{-\nu }$, and, by translation invariance, also $\Vert (1- \mathbb {E}_{\Lambda _k+(m,0)})\beta _\varepsilon (j_{m})\Vert \le \Vert \beta _\varepsilon (j_{0})\Vert _\nu \,(1+k)^{-\nu }$. Thus, defining $j_m^{k,\varepsilon }:= \mathbb {E}_{\Lambda _k+(m,0)}\beta _\varepsilon (j_{m})$ and choosing in each summand $k=\frac{|m-\ell |}{4}$, we find for $\nu >1$

$$\begin{aligned} \omega _\varepsilon (J_L^2) - \omega _\varepsilon (J_L)^2= &   \sum _{m,\ell =1}^L \left( \omega _0 ( j^{\frac{|m-\ell |}{4},\varepsilon }_{m}\, j^{\frac{|m-\ell |}{4},\varepsilon }_{\ell } )\right. \\    &   \left. - \omega _0 ( j^{\frac{|m-\ell |}{4},\varepsilon }_{m} )\omega _0 ( j^{\frac{|m-\ell |}{4},\varepsilon }_{\ell }) + \mathcal {O}((1+\tfrac{|m-\ell |}{4})^{-\nu } ) \right) \\\le &   \sum _{ m,\ell =1 }^L C\,\textrm{e}^{-c|m-\ell |}+ \mathcal {O}( L) \;=\; \mathcal {O}(L). \end{aligned}$$

In the last inequality we used the uniform exponential decay of correlations in gapped ground states of Hamiltonians with short-range interactions proved in [24, Theorem 2.8].

$$\square $$

### Remark 5.6

Notice how (15) yields in particular the well-known fact that, in the limit $L \rightarrow \infty$, Hall conductance and Hall conductivity agree in 2-dimensions (up to terms which are smaller than any arbitrarily large power of the strength of the inducing electric field). In particular, the Hall conductance in this thermodynamic limit is defined in terms of a “per-length” (rather than per-volume, or more precisely per-surface in $d=2$) expectation: see [38, Equation (5.16)] and references therein for a discussion on this point.

## Data Availability

Data sharing is not applicable to this article as no datasets were generated or analysed during the current study.

## Appendix A: Estimate for the Commutator of Translated Observables

The following result will be used as an essential ingredient in the proofs of several propositions and lemmas in the other Appendices.

### Lemma A.1

Let $A \in D_\infty$ and $B \in D_\infty$. Let *T* be a translation and $\gamma \in \mathbb {Z}^d$. Then for all $\nu ,m\in \mathbb {N}_0$

$$\begin{aligned} \Vert {[T_{\gamma }A,B]}\Vert _\nu \;\le \; 4^{\nu +m+3}\frac{\Vert {A}\Vert _{\nu +m}\Vert {B}\Vert _{\nu +m}}{(1 + \Vert {\gamma }\Vert _{\infty })^{m}}\,. \end{aligned}$$

### Proof

The proof is constituted by a somewhat lengthy computation. Our goal is to find the desired upper bound for

$$\begin{aligned} \Vert {[T_{\gamma }A,B]}\Vert _\nu&= \Vert {[T_{\gamma }A,B]}\Vert + \sup _{k\in \mathbb {N}_0} \Vert {(1-\mathbb {E}_{\Lambda _k})[T_{\gamma }A,B](1+k)^\nu }\Vert ~. \end{aligned}$$

We start by bounding $\Vert {[T_{\gamma }A,B]}\Vert$. For this we split the commutator into a sum of three terms, where we define $\tilde{\gamma } = \lfloor {\frac{\Vert {\gamma }\Vert _\infty }{2}}\rfloor$ for better readability:

$$\begin{aligned} {[}T_{\gamma }A,B] =&[T_\gamma \mathbb {E}_{\Lambda _{\tilde{\gamma }}}A,\mathbb {E}_{\Lambda _{\tilde{\gamma }}}B] +[T_\gamma (1-\mathbb {E}_{\Lambda _{\tilde{\gamma }}})A,\mathbb {E}_{\Lambda _{\tilde{\gamma }}}B] + [T_\gamma A,(1-\mathbb {E}_{\Lambda _{\tilde{\gamma }}})B]~. \end{aligned}$$

Now we bound each term separately. By definition of $\tilde{\gamma }$ it holds that $(\Lambda _{\tilde{\gamma }}+\gamma ) \cap \Lambda _{\tilde{\gamma }}$ is empty, except when $\gamma =0$. Since *B* is gauge-invariant and $\mathbb {E}_{\Lambda _k}$ preserves this property (Proposition 2.1), the first term is 0 except when $\gamma =0$:

$$\begin{aligned} \Vert {[T_\gamma \mathbb {E}_{\Lambda _{\tilde{\gamma }}}A,\mathbb {E}_{\Lambda _{\tilde{\gamma }}}B]}\Vert&= \delta _{\gamma ,0} \Vert {[ \mathbb {E}_{\Lambda _{0}}A,\mathbb {E}_{\Lambda _{0}}B]}\Vert \, . \end{aligned}$$

Using that $\Vert {\mathbb {E}_{\Lambda _0}}\Vert =1$ and $\delta _{\gamma ,0} \le \frac{1}{({1+ \Vert {\gamma }\Vert _\infty })^m}$ we get

$$\begin{aligned} \Vert {[T_\gamma \mathbb {E}_{\Lambda _{\tilde{\gamma }}}A,\mathbb {E}_{\Lambda _{\tilde{\gamma }}}B]}\Vert \le 2 \Vert {A}\Vert \Vert {B}\Vert \frac{1}{({1+ \Vert {\gamma }\Vert _\infty })^m} \, . \end{aligned}$$

For the second term we first estimate the commutator and use that $\Vert {T_{\gamma }}\Vert = \Vert {\mathbb {E}_{\Lambda _{\tilde{\gamma }}}}\Vert = 1$ to get

$$\begin{aligned} \Vert {[T_\gamma (1-\mathbb {E}_{\Lambda _{\tilde{\gamma }}})A,\mathbb {E}_{\Lambda _{\tilde{\gamma }}}B]}\Vert \le 2 \Vert {(1-\mathbb {E}_{\Lambda _{\tilde{\gamma }}})A}\Vert \Vert {B}\Vert ~. \end{aligned}$$

From the definition of $\Vert {\cdot }\Vert _m$ and the fact that $(1+\Vert {\gamma }\Vert _{\infty })^m \le 2^m (1+\tilde{\gamma })^m$ we see

$$\begin{aligned} \Vert {[T_\gamma (1-\mathbb {E}_{\Lambda _{\tilde{\gamma }}})A,\mathbb {E}_{\Lambda _{\tilde{\gamma }}}B]}\Vert \le 2\Vert {A}\Vert _m\Vert {B}\Vert \frac{1}{(1+\tilde{\gamma })^m} \le 2\Vert {A}\Vert _m\Vert {B}\Vert \frac{2^m}{(1+\Vert {\gamma }\Vert _{\infty })^m}. \end{aligned}$$

The third term can be handled by the same reasoning:

$$\begin{aligned} \Vert {[T_\gamma A,(1-\mathbb {E}_{\Lambda _{\tilde{\gamma }}})B]}\Vert&\le 2\Vert {A}\Vert \Vert {(1-\mathbb {E}_{\Lambda _{\tilde{\gamma }}})B}\Vert \le 2\Vert {A}\Vert \Vert {B}\Vert _m \frac{1}{(1+\tilde{\gamma })^m}\\  &\le 2\Vert {A}\Vert \Vert {B}\Vert _m \frac{2^m}{(1+\Vert {\gamma }\Vert _{\infty })^m}\, . \end{aligned}$$

With all three estimates and using that $\Vert {A}\Vert \le \Vert {A}\Vert _m$ we have shown that

$$\begin{aligned} \Vert {[T_{\gamma }A,B]}\Vert \le&6\Vert {A}\Vert _m \Vert {B}\Vert _m\frac{2^m}{(1+\Vert {\gamma }\Vert _{\infty })^m}~. \end{aligned}$$

Next we deal with the supremum part of $\Vert {[T_{\gamma }A,B]}\Vert _\nu$. For this we choose a $k\in \mathbb {N}_0$ and consider the expression

$$\begin{aligned} \Vert {(1-\mathbb {E}_{\Lambda _k})[T_\gamma A ,B]}\Vert (1+k)^\nu ~, \end{aligned}$$

for which we would like to find a suitable *k*-indepentent bound. To do this we again split the commutator as before but this time we further split the second and third therm so that we end up with five terms in total:

$$\begin{aligned} \begin{aligned} (1-\mathbb {E}_{\Lambda _k}) [T_\gamma A,B]~(1+k)^\nu =&\;(1-\mathbb {E}_{\Lambda _k}) [T_\gamma \mathbb {E}_{\Lambda _{\tilde{\gamma }}}A,\mathbb {E}_{\Lambda _{\tilde{\gamma }}}B]~(1+k)^\nu \\  &+ (1-\mathbb {E}_{\Lambda _k}) [\mathbb {E}_{\Lambda _k} T_\gamma (1-\mathbb {E}_{\Lambda _{\tilde{\gamma }}})A,\mathbb {E}_{\Lambda _{\tilde{\gamma }}}B]~(1+k)^\nu \\  &+ (1-\mathbb {E}_{\Lambda _k}) [(1-\mathbb {E}_{\Lambda _k}) T_\gamma (1-\mathbb {E}_{\Lambda _{\tilde{\gamma }}})A,\mathbb {E}_{\Lambda _{\tilde{\gamma }}}B]~(1+k)^\nu \\  &+ (1-\mathbb {E}_{\Lambda _k}) [\mathbb {E}_{\Lambda _k}T_\gamma A,(1-\mathbb {E}_{\Lambda _{\tilde{\gamma }}})B]~(1+k)^\nu \\  &+ (1-\mathbb {E}_{\Lambda _k}) [(1- \mathbb {E}_{\Lambda _k})T_\gamma A,(1-\mathbb {E}_{\Lambda _{\tilde{\gamma }}})B]~(1+k)^\nu \, . \end{aligned} \end{aligned}$$

To easily refer to the norms of these five terms we define

$$\begin{aligned} \mathrm {(I)}&:=\Vert {(1-\mathbb {E}_{\Lambda _k}) [T_\gamma \mathbb {E}_{\Lambda _{\tilde{\gamma }}}A,\mathbb {E}_{\Lambda _{\tilde{\gamma }}}B]}\Vert ~(1+k)^\nu \, ,\\ \mathrm {(II)}&:=\Vert {(1-\mathbb {E}_{\Lambda _k}) [\mathbb {E}_{\Lambda _k} T_\gamma (1-\mathbb {E}_{\Lambda _{\tilde{\gamma }}})A,\mathbb {E}_{\Lambda _{\tilde{\gamma }}}B]}\Vert ~(1+k)^\nu \, ,\\ \mathrm {(III)}&:=\Vert {(1-\mathbb {E}_{\Lambda _k}) [(1-\mathbb {E}_{\Lambda _k}) T_\gamma (1-\mathbb {E}_{\Lambda _{\tilde{\gamma }}})A,\mathbb {E}_{\Lambda _{\tilde{\gamma }}}B]}\Vert ~(1+k)^\nu \, ,\\ \mathrm {(IV)}&:=\Vert {(1-\mathbb {E}_{\Lambda _k}) [\mathbb {E}_{\Lambda _k}T_\gamma A,(1-\mathbb {E}_{\Lambda _{\tilde{\gamma }}})B]}\Vert ~(1+k)^\nu \, ,\\ \mathrm {(V)}&:=\Vert {(1-\mathbb {E}_{\Lambda _k}) [(1- \mathbb {E}_{\Lambda _k})T_\gamma A,(1-\mathbb {E}_{\Lambda _{\tilde{\gamma }}})B]}\Vert ~(1+k)^\nu ~. \end{aligned}$$

We see that the first term can be dealt with in the same way we did before:

$$\begin{aligned} \mathrm {(I)} =\Vert {(1-\mathbb {E}_{\Lambda _k}) \delta _{\gamma ,0}[\mathbb {E}_{\Lambda _0}A,\mathbb {E}_{\Lambda _0}B]}\Vert ~(1+k)^\nu \, . \end{aligned}$$

The expression is equal to zero, because $\Lambda _0$ is a subset of $\Lambda _k$ and $\mathbb {E}_{\Lambda _k}$ acts as the identity on $\mathcal {A}_{\Lambda _k}$.

For the second term we can use the first property from Proposition 2.1 to move $(1-\mathbb {E}_{\Lambda _k})$ to the second slot of the commutator and then estimate the commutator:

$$\begin{aligned} \mathrm {(II)}&=\Vert {(1-\mathbb {E}_{\Lambda _k}) [\mathbb {E}_{\Lambda _k} T_\gamma (1-\mathbb {E}_{\Lambda _{\tilde{\gamma }}})A,\mathbb {E}_{\Lambda _{\tilde{\gamma }}}B]}\Vert ~(1+k)^\nu \\&=\Vert {[\mathbb {E}_{\Lambda _k} T_\gamma (1-\mathbb {E}_{\Lambda _{\tilde{\gamma }}})A,(1-\mathbb {E}_{\Lambda _k}) \mathbb {E}_{\Lambda _{\tilde{\gamma }}}B]}\Vert ~(1+k)^\nu \\&\le 2\Vert {\mathbb {E}_{\Lambda _k} T_\gamma (1-\mathbb {E}_{\Lambda _{\tilde{\gamma }}})A}\Vert \Vert {(1-\mathbb {E}_{\Lambda _k}) \mathbb {E}_{\Lambda _{\tilde{\gamma }}}B}\Vert ~(1+k)^\nu \,. \end{aligned}$$

Next we use that $\Vert {T_\gamma }\Vert = \Vert {\mathbb {E}_{\Lambda _k}}\Vert = \Vert {\mathbb {E}_{\Lambda _{\tilde{\gamma }}}}\Vert = 1$ and that we can swap $(1-\mathbb {E}_{\Lambda _k})$ and $\mathbb {E}_{\Lambda _{\tilde{\gamma }}}$ by Proposition 2.1 to get

$$\begin{aligned} \mathrm {(II)}&\le 2\Vert {(1-\mathbb {E}_{\Lambda _{\tilde{\gamma }}})A}\Vert \Vert {(1-\mathbb {E}_{\Lambda _k}) B}\Vert ~(1+k)^\nu ~. \end{aligned}$$

We estimate by the decay norm and use $(1+\Vert {\gamma }\Vert _{\infty })^m \le 2^m (1+\tilde{\gamma })^m$:

$$\begin{aligned} \mathrm {(II)} \le 2\Vert {A}\Vert _{m} \Vert {B}\Vert _{\nu } \frac{(1+k)^\nu }{(1+\tilde{\gamma })^m (1+k)^\nu } \le 2\Vert {A}\Vert _m \Vert {B}\Vert _\nu \frac{2^m}{(1+\Vert {\gamma }\Vert _\infty )^{m}} \, . \end{aligned}$$

We deal with the third term by using $\Vert {1-\mathbb {E}_{\Lambda _k}}\Vert \le 2$, and then further estimating via the commutator and $\Vert {\mathbb {E}_{\Lambda _{\tilde{\gamma }}}}\Vert = 1$:

$$\begin{aligned} \mathrm {(III)}&=\Vert {(1-\mathbb {E}_{\Lambda _k})[(1-\mathbb {E}_{\Lambda _k}) T_\gamma (1-\mathbb {E}_{\Lambda _{\tilde{\gamma }}})A,\mathbb {E}_{\Lambda _{\tilde{\gamma }}}B]}\Vert (1+k)^\nu \\&\le 2\Vert {[(1-\mathbb {E}_{\Lambda _k}) T_\gamma (1-\mathbb {E}_{\Lambda _{\tilde{\gamma }}})A,\mathbb {E}_{\Lambda _{\tilde{\gamma }}}B]}\Vert (1+k)^\nu \\&\le 4\Vert {(1-\mathbb {E}_{\Lambda _k}) T_\gamma (1-\mathbb {E}_{\Lambda _{\tilde{\gamma }}})A}\Vert \Vert {B}\Vert (1+k)^\nu \, . \end{aligned}$$

By applying Lemma 2.8 we pull $T_{\gamma }$ past the conditional expectation and use $\Vert {T_{\gamma }}\Vert = 1$ to get

$$\begin{aligned} \mathrm {(III)}&\le 4\Vert {(1-\mathbb {E}_{(\Lambda _{k}-\gamma )}) (1-\mathbb {E}_{\Lambda _{\tilde{\gamma }}})A}\Vert \Vert {B}\Vert (1+k)^\nu ~. \end{aligned}$$

We observe that using $\Vert {1-\mathbb {E}_{\Lambda _k-\gamma }}\Vert \le 2$ one gets

$$\begin{aligned} \Vert {(1-\mathbb {E}_{(\Lambda _{k}-\gamma )}) (1-\mathbb {E}_{\Lambda _{\tilde{\gamma }}})A}\Vert \le 2\Vert {(1-\mathbb {E}_{\Lambda _{\tilde{\gamma }}})A}\Vert \le \frac{2\Vert {A}\Vert _{\nu +m}}{(1+\tilde{\gamma })^{\nu +m}}\, . \end{aligned}$$

Now if $k-\Vert {\gamma }\Vert _\infty \ge 0$ we have $\Lambda _{k-\Vert {\gamma }\Vert _\infty } \subseteq \Lambda _k -\gamma$, which together with Proposition 2.1 and estimating by the decay norm gives us the stronger bound

$$\begin{aligned} \begin{aligned} \Vert {(1-\mathbb {E}_{(\Lambda _{k}-\gamma )}) (1-\mathbb {E}_{\Lambda _{\tilde{\gamma }}})A}\Vert&\le \; 2 \Vert {(1-\mathbb {E}_{\Lambda _{k-\Vert {\gamma }\Vert _\infty }}) (1-\mathbb {E}_{\Lambda _{\tilde{\gamma }}})A}\Vert \\  &=\; 2\Vert {(1-\mathbb {E}_{\Lambda _{\max (\tilde{\gamma },k-\Vert {\gamma }\Vert _\infty )}}) A}\Vert \\  &\le \; \frac{2\Vert {A}\Vert _{\nu +m}}{(1 + \max (\tilde{\gamma },k-\Vert {\gamma }\Vert _\infty ))^{\nu + m}}\, . \end{aligned} \end{aligned}$$

Combining the two statements, one sees that the stronger bound in fact always holds, which results in

$$\begin{aligned} \mathrm {(III)}&\le 8\Vert {A}\Vert _{\nu +m} \Vert {B}\Vert \frac{ (1+k)^\nu }{(1 + \max (\tilde{\gamma },k-\Vert {\gamma }\Vert _\infty ))^{\nu + m}}\, . \end{aligned}$$

The function $x \mapsto \frac{ (1+x)^\nu }{(1 + \max (\tilde{\gamma },x-\Vert {\gamma }\Vert _\infty ))^{\nu + m}}$ is strictly increasing for $x \,<\, \tilde{\gamma }+ \Vert {\gamma }\Vert _\infty$ and strictly decreasing for $x \,>\, \tilde{\gamma }+ \Vert {\gamma }\Vert _\infty$, thus

$$\begin{aligned} \mathrm {(III)}&\le 8\Vert {A}\Vert _{\nu +m} \Vert {B}\Vert \frac{ (1+\tilde{\gamma }+ \Vert {\gamma }\Vert _\infty )^\nu }{(1 + \max (\tilde{\gamma },\tilde{\gamma }+ \Vert {\gamma }\Vert _\infty -\Vert {\gamma }\Vert _\infty ))^{\nu + m}} \\  &= 8\Vert {A}\Vert _{\nu +m} \Vert {B}\Vert \frac{ (1+\tilde{\gamma }+ \Vert {\gamma }\Vert _\infty )^\nu }{(1 + \tilde{\gamma })^{\nu + m}}\, . \end{aligned}$$

We use $(1+\Vert {\gamma }\Vert _{\infty })^{\nu +m} \le 2^{\nu +m} (1+\tilde{\gamma })^{\nu +m}$ and $(1 + \Vert {\gamma }\Vert _{\infty }+\tilde{\gamma })^\nu \le 2^\nu (1+\Vert {\gamma }\Vert _{\infty })^\nu$ to obtain

$$\begin{aligned} \mathrm {(III)} \le 8\Vert {A}\Vert _{\nu +m}\Vert {B}\Vert \frac{2^\nu 2^{\nu +m}(1+\Vert {\gamma }\Vert _{\infty })^\nu }{(1+\Vert {\gamma }\Vert _{\infty })^{\nu +m}} \le 8\Vert {A}\Vert _{\nu +m}\Vert {B}\Vert \frac{4^{\nu +m}}{(1+\Vert {\gamma }\Vert _{\infty })^{m}}~. \end{aligned}$$

For the fourth term we move $(1-\mathbb {E}_{\Lambda _k})$ inside the commutator, estimate the commutator and use $\Vert {\mathbb {E}_{\Lambda _k}}\Vert = \Vert {T_{\gamma }}\Vert = 1$, which results in

$$\begin{aligned} \mathrm {(IV)}&= \Vert {(1-\mathbb {E}_{\Lambda _k})[\mathbb {E}_{\Lambda _k}T_\gamma A,(1-\mathbb {E}_{\Lambda _{\tilde{\gamma }}})B]}\Vert (1+k)^\nu \\&=\;\Vert {[\mathbb {E}_{\Lambda _k}T_\gamma A,(1-\mathbb {E}_{\Lambda _k})(1-\mathbb {E}_{\Lambda _{\tilde{\gamma }}})B]}\Vert (1+k)^\nu \\&\le 2\Vert {\mathbb {E}_{\Lambda _k}T_\gamma A}\Vert \Vert {(1-\mathbb {E}_{\Lambda _k})(1-\mathbb {E}_{\Lambda _{\tilde{\gamma }}})B}\Vert (1+k)^\nu \\  &\le \; 2\Vert {A}\Vert \Vert {(1-\mathbb {E}_{\Lambda _k})(1-\mathbb {E}_{\Lambda _{\tilde{\gamma }}})B}\Vert (1+k)^\nu ~. \end{aligned}$$

Applying Proposition 2.1 in the form of $(1-\mathbb {E}_{\Lambda _k})(1-\mathbb {E}_{\Lambda _{\tilde{\gamma }}}) = 1-\mathbb {E}_{\Lambda _{\max (\tilde{\gamma },k)}}$ and estimating the result with the decay norm leaves us with

$$\begin{aligned} \mathrm {(IV)} \;\le \; 2\Vert {A}\Vert \Vert {(1-\mathbb {E}_{\Lambda _{\max (\tilde{\gamma },k)}})B}\Vert (1+k)^\nu \;\le \; 2\Vert {A}\Vert \Vert {B}\Vert _{\nu +m} \frac{(1+k)^\nu }{(1+\max (\tilde{\gamma },k))^{\nu +m}}~. \end{aligned}$$

Now we simply estimate the maximum and then use $(1+\Vert {\gamma }\Vert _{\infty })^{m} \le 2^{m} (1+\tilde{\gamma })^{m}$:

$$\begin{aligned} \mathrm {(IV)}&\le \; 2\Vert {A}\Vert \Vert {B}\Vert _{\nu +m} \frac{(1+k)^\nu }{(1+\tilde{\gamma })^{m}(1+k)^{\nu }} \\  &= \;2\Vert {A}\Vert \Vert {B}\Vert _{\nu +m} \frac{1}{(1+\tilde{\gamma })^{m}}\; \le \;2\Vert {A}\Vert \Vert {B}\Vert _{\nu +m} \frac{2^m}{(1+\Vert {\gamma }\Vert _\infty )^{m}} \, . \end{aligned}$$

We tackle the fifth term by estimating with $\Vert {1-\mathbb {E}_{\Lambda _k}}\Vert \le 2$ and the normal commutator bound. This results in

$$\begin{aligned} \begin{aligned} \mathrm {(V)}&= \;\Vert {(1-\mathbb {E}_{\Lambda _k}) [(1- \mathbb {E}_{\Lambda _k})T_\gamma A,(1-\mathbb {E}_{\Lambda _{\tilde{\gamma }}})B]}\Vert (1+k)^\nu \\  &\le \;2\Vert {[(1- \mathbb {E}_{\Lambda _k})T_\gamma A,(1-\mathbb {E}_{\Lambda _{\tilde{\gamma }}})B]}\Vert (1+k)^\nu \\  &\le \; 4\Vert {(1- \mathbb {E}_{\Lambda _k})T_\gamma A}\Vert \Vert {(1-\mathbb {E}_{\Lambda _{\tilde{\gamma }}})B}\Vert (1+k)^\nu \,. \end{aligned} \end{aligned}$$

Using Lemma 2.8 and $\Vert {T_{\gamma }}\Vert =1$ we obtain

$$\begin{aligned} \mathrm {(V)}&\le \; 4\Vert {T_\gamma (1- \mathbb {E}_{(\Lambda _k-\gamma )})A}\Vert \Vert {(1-\mathbb {E}_{\Lambda _{\tilde{\gamma }}})B}\Vert (1+k)^\nu \\  &\le \; 4\Vert {(1- \mathbb {E}_{(\Lambda _k-\gamma )})A}\Vert \Vert {(1-\mathbb {E}_{\Lambda _{\tilde{\gamma }}})B}\Vert (1+k)^\nu ~. \end{aligned}$$

Similarly to before we observe that

$$\begin{aligned} \Vert {(1- \mathbb {E}_{(\Lambda _k-\gamma )})A}\Vert \le 2 \Vert {A}\Vert \le 2 \frac{\Vert {A}\Vert _{\nu +m}}{(1 + 0)^{\nu +m}} \end{aligned}$$

and if $k-\Vert {\gamma }\Vert _{\infty } \ge 0$, we have $\Lambda _{(k-\Vert {\gamma }\Vert _{\infty })} \subseteq (\Lambda _{k}-\gamma )$, and therefore get the stronger bound

$$\begin{aligned} \Vert {(1- \mathbb {E}_{(\Lambda _k-\gamma )})A}\Vert \;\le \;2\Vert {(1- \mathbb {E}_{\Lambda _{(k-\Vert {\gamma }\Vert _{\infty })}})A}\Vert \;\le \;2 \frac{\Vert {A}\Vert _{\nu +m}}{(1 + k-\Vert {\gamma }\Vert _{\infty })^{\nu +m}}~. \end{aligned}$$

Together these two statements show that we always have

$$\begin{aligned} \Vert {(1- \mathbb {E}_{(\Lambda _k-\gamma )})A}\Vert \;\le \; 2 \frac{\Vert {A}\Vert _{\nu +m}}{(1 + \max (0,k-\Vert {\gamma }\Vert _{\infty }))^{\nu +m}}~. \end{aligned}$$

We use this observation and the decay norm to further estimate $\mathrm {(V)}$:

$$\begin{aligned} \mathrm {(V)}&\le \; 8 \frac{\Vert {A}\Vert _{\nu +m}}{(1 + \max (0,k-\Vert {\gamma }\Vert _{\infty }))^{\nu +m}}\Vert {(1-\mathbb {E}_{\Lambda _{\tilde{\gamma }}})B}\Vert (1+k)^\nu \\  &\le \;\frac{8\Vert {A}\Vert _{\nu +m}\Vert {B}\Vert _{\nu +m}(1+k)^\nu }{(1 + \max (0,k-\Vert {\gamma }\Vert _{\infty }))^{\nu +m}(1+\tilde{\gamma })^{\nu +m}} \, . \end{aligned}$$

Next we again use $(1+\Vert {\gamma }\Vert _{\infty })^{\nu +m} \le 2^{\nu +m} (1+\tilde{\gamma })^{\nu +m}$ and

$$\begin{aligned} 1 + \max (\Vert {\gamma }\Vert _{\infty },k) \le (1 + \max (0,k-\Vert {\gamma }\Vert _{\infty }))(1+\Vert {\gamma }\Vert _{\infty }) \end{aligned}$$

to get

$$\begin{aligned} \mathrm {(V)} \le \frac{8\Vert {A}\Vert _{\nu +m}\Vert {B}\Vert _{\nu +m}2^{\nu +m}(1+k)^\nu }{(1 + \max (0,k-\Vert {\gamma }\Vert _{\infty }))^{\nu +m}(1+\Vert {\gamma }\Vert _{\infty })^{\nu +m}}\le \frac{8\Vert {A}\Vert _{\nu +m}\Vert {B}\Vert _{\nu +m}2^{\nu +m}(1+k)^\nu }{(1 + \max (\Vert {\gamma }\Vert _{\infty },k))^{\nu +m}}~. \end{aligned}$$

By estimating the maximum we conclude

$$\begin{aligned} \mathrm {(V)} \;\le \;\frac{8\Vert {A}\Vert _{\nu +m}\Vert {B}\Vert _{\nu +m}2^{\nu +m}(1+k)^\nu }{(1 + \Vert {\gamma }\Vert _{\infty })^{m}(1 + k)^{\nu }}\;= \; \frac{8\Vert {A}\Vert _{\nu +m}\Vert {B}\Vert _{\nu +m}2^{\nu +m}}{(1 + \Vert {\gamma }\Vert _{\infty })^{m}}~. \end{aligned}$$

We combine the estimates for all four non-zero terms, this shows that

$$\begin{aligned} \sup _{k\in \mathbb {N}_0} \Vert {(1-\mathbb {E}_{\Lambda _k})[T_\gamma A ,B]}\Vert (1+k)^\nu&\le ~ \frac{20\Vert {A}\Vert _{\nu +m}\Vert {B}\Vert _{\nu +m}4^{\nu +m}}{(1 + \Vert {\gamma }\Vert _{\infty })^{m}}~. \end{aligned}$$

In total we have shown that

$$\begin{aligned} \Vert {[T_{\gamma }A,B]}\Vert _\nu&=\Vert {[T_{\gamma }A,B]}\Vert + \sup _{k\in \mathbb {N}_0} \Vert (1-\mathbb {E}_{\Lambda _k})[T_\gamma A ,B] \Vert (1+k)^\nu \\&\le 6\Vert {A}\Vert _m \Vert {B}\Vert _m\frac{2^m}{(1+\Vert {\gamma }\Vert _{\infty })^m} + \frac{20\Vert {A}\Vert _{\nu +m}\Vert {B}\Vert _{\nu +m}4^{\nu +m}}{(1 + \Vert {\gamma }\Vert _{\infty })^{m}} \\  &\le 4^{\nu +m+3}\frac{\Vert {A}\Vert _{\nu +m}\Vert {B}\Vert _{\nu +m}}{(1 + \Vert {\gamma }\Vert _{\infty })^{m}}~.\; \end{aligned}$$

$$\square $$

## Appendix B: Proofs of Basic Propositions in Sect. 2

### Proof of Lemma 2.3

Let $k,\nu \in \mathbb {N}_0$ and $A,B\in D_{\infty }$. Then

$$\begin{aligned} \Vert A\,B - \mathbb {E}_{\Lambda _k} (A\,B) \Vert&\le \Vert A\,B - \mathbb {E}_{\Lambda _k} (A\,\mathbb {E}_{\Lambda _k}B) \Vert + \Vert \mathbb {E}_{\Lambda _k} (A\, (B - \mathbb {E}_{\Lambda _k}B)) \Vert \\&= \Vert A\,B - (\mathbb {E}_{\Lambda _k} A) \, (\mathbb {E}_{\Lambda _k}B) \Vert + \Vert \mathbb {E}_{\Lambda _k} (A\, (B - \mathbb {E}_{\Lambda _k}B)) \Vert \\&\le \Vert A\, B - A\, \mathbb {E}_{\Lambda _k} B \Vert + \Vert A\, \mathbb {E}_{\Lambda _k} B - (\mathbb {E}_{\Lambda _k} A) \, (\mathbb {E}_{\Lambda _k}B) \Vert \\  &\quad + \Vert \mathbb {E}_{\Lambda _k} (A\, (B - \mathbb {E}_{\Lambda _k}B)) \Vert \\&\le \Vert A \Vert \, \Vert B - \mathbb {E}_{\Lambda _k}B \Vert + \Vert A -\mathbb {E}_{\Lambda _k}A \Vert \, \Vert B \Vert + \Vert A \Vert \, \Vert B - \mathbb {E}_{\Lambda _k}B \Vert \end{aligned}$$

and therefore

$$\begin{aligned}&\Vert A\, B \Vert + \sup _{k\in \mathbb {N}_0} \Vert A\, B - \mathbb {E}_{\Lambda _k}(A\,B) \Vert \, (1+k)^\nu \\&\quad \le 2 \, (\Vert {A}\Vert + \sup _{k\in \mathbb {N}_0}( \Vert {A-\mathbb {E}_{\Lambda _k}A}\Vert (1+k)^\nu )) \, (\Vert {B}\Vert + \sup _{k\in \mathbb {N}_0}( \Vert {B-\mathbb {E}_{\Lambda _k}B}\Vert (1+k)^\nu ) \, . \end{aligned}$$

For the second statement note that, by assumption,

$$\begin{aligned} \sup _{l\in \mathbb {N}_0} \Vert A - \mathbb {E}_{\Lambda _l}A \Vert \, (1+l)^{\nu +1} < \infty \, . \end{aligned}$$

Therefore, $\lim _{l\rightarrow \infty } \Vert A - \mathbb {E}_{\Lambda _l}A \Vert \, (1+l)^{\nu } = 0$. Hence in

$$\begin{aligned} \Vert A -\mathbb {E}_{\Lambda _k}A \Vert _{\nu }&= \Vert A -\mathbb {E}_{\Lambda _k}A \Vert + \sup _{l\in \mathbb {N}_0}\Vert (1-\mathbb {E}_{\Lambda _l})\, (1-\mathbb {E}_{\Lambda _k})\, A \Vert \, (1+l)^{\nu }\\&= \Vert A -\mathbb {E}_{\Lambda _k}A \Vert + \sup _{l\in \mathbb {N}_0}\Vert (1-\mathbb {E}_{\Lambda _{\max (l,k)}})\, A \Vert \, (1+l)^{\nu }\\&= \Vert A -\mathbb {E}_{\Lambda _k}A \Vert + \sup _{\begin{array}{c} l\in \mathbb {N}_0 \\ l\ge k \end{array}}\Vert (1-\mathbb {E}_{\Lambda _l})\, A \Vert \, (1+l)^{\nu }\, \end{aligned}$$

both terms go to 0 as $k\rightarrow \infty$. $\square$

### Proof of Lemma 2.5

For $M\in P_0(\mathbb {Z}^d)$ with $0 \notin M$ we define $\Lambda _M$ as the largest box around 0 that does not intersect *M* and use this with the fact that $\varPhi (M)$ commutes with all operators supported outside *M* to rewrite the sum

$$\begin{aligned}&\sum _{M\in P_0(\mathbb {Z}^d)} \Vert {[\varPhi (M),A]}\Vert _\nu = \sum _{\begin{array}{c} M\in P_0(\mathbb {Z}^d)\\ 0\in M \end{array}} \Vert {[\varPhi (M),A]}\Vert _\nu +\sum _{k=0}^\infty \sum _{\begin{array}{c} M\in P_0(\mathbb {Z}^d)\\ 0 \notin M \\ \Lambda _M = \Lambda _k \end{array}} \Vert {[\varPhi (M),A ]}\Vert _\nu \\&\quad = \sum _{\begin{array}{c} M\in P_0(\mathbb {Z}^d)\\ 0\in M \end{array}} \Vert {[\varPhi (M),A]}\Vert _\nu + \sum _{k=0}^\infty \sum _{\begin{array}{c} M\in P_0(\mathbb {Z}^d)\\ 0\notin M \\ \Lambda _M = \Lambda _k \end{array}} \Vert {[\varPhi (M),A - \mathbb {E}_{\Lambda _k}A ]}\Vert _\nu \\&\quad {=}{:}\; S_1 + S_2 \,. \end{aligned}$$

To estimate $S_1$, we use the first property in Lemma 2.3 and get

$$\begin{aligned} S_1&\le \sum _{\begin{array}{c} M\in P_0(\mathbb {Z}^d)\\ 0\in M \end{array}} 4\,\Vert {\varPhi (M)}\Vert _\nu \, \Vert {A}\Vert _\nu \, . \end{aligned}$$

Now we observe that each *M* in the sum is contained in a box around 0 with side length $2\, \textrm{diam}(M)$, which allows us to estimate $\Vert {\varPhi (M)}\Vert _\nu$ by $3 \,(1+ \textrm{diam}(M))^\nu \,\Vert {\varPhi (M)}\Vert$. We thus arrive at

16 $$\begin{aligned} S_1 \le \sum _{\begin{array}{c} M\in P_0(\mathbb {Z}^d)\\ 0\in M \end{array}} 12 \,(1+ \textrm{diam}(M))^\nu \,\Vert {\varPhi (M)}\Vert \, \Vert {A}\Vert _\nu \le 12 \, \Vert {\varPhi }\Vert _\nu \, \Vert {A}\Vert _\nu \, . \end{aligned}$$

We can estimate $S_2$ by splitting it into parts associated to each lattice site while summing over sets multiple times to get

$$\begin{aligned} S_2&\le \sum _{x\in \mathbb {Z}^d} \sum _{k=0}^\infty \sum _{\begin{array}{c} M\in P_0(\mathbb {Z}^d)\\ 0 \notin M, \, x\in M\\ \Lambda _M = \Lambda _k \end{array}} \Vert {[\varPhi (M),A - \mathbb {E}_{\Lambda _k}A ]}\Vert _\nu \, . \end{aligned}$$

The decay norm of the commutator can again be estimated as in Lemma 2.3, giving us

$$\begin{aligned} S_2&\le \sum _{x\in \mathbb {Z}^d} \sum _{k=0}^\infty \sum _{\begin{array}{c} M\in P_0(\mathbb {Z}^d)\\ 0 \notin M, \, x\in M\\ \Lambda _M = \Lambda _k \end{array}} 4 \, \Vert {\varPhi (M)}\Vert _\nu \, \Vert {A - \mathbb {E}_{\Lambda _k}A }\Vert _\nu \,. \end{aligned}$$

Similarly to before, we observe that *M* is always contained in a box around 0 with side length $2 \, \left( \Vert {x}\Vert _\infty + \textrm{diam}(M) \right)$, which we use to estimate the decay norm of $\varPhi (M)$

$$\begin{aligned} S_2&\le \sum _{x\in \mathbb {Z}^d} \sum _{k=0}^\infty \sum _{\begin{array}{c} M\in P_0(\mathbb {Z}^d)\\ 0 \notin M, \, x\in M\\ \Lambda _M = \Lambda _k \end{array}} 12 \, (1 + \Vert {x}\Vert _\infty + \textrm{diam}(M))^\nu \, \Vert {\varPhi (M)}\Vert \, \Vert {A - \mathbb {E}_{\Lambda _k}A }\Vert _\nu \\&\le \sum _{x\in \mathbb {Z}^d} \sum _{k=0}^\infty \sum _{\begin{array}{c} M\in P_0(\mathbb {Z}^d)\\ 0 \notin M, \, x\in M\\ \Lambda _M = \Lambda _k \end{array}} 12 \, (1 + \Vert {x}\Vert _\infty )^\nu (1 + \textrm{diam}(M))^\nu \, \Vert {\varPhi (M)}\Vert \, \Vert {A - \mathbb {E}_{\Lambda _k}A }\Vert _\nu \,. \end{aligned}$$

Each *M* in the sum contains *x* and some element that has a distance of $k+1$ from the origin, so $\textrm{diam}(M) \ge |{\Vert {x}\Vert _\infty - k -1}|$. This allows us to further estimate the expression as

$$\begin{aligned} S_2&\le \sum _{x\in \mathbb {Z}^d} \sum _{k=0}^\infty \sum _{\begin{array}{c} M\in P_0(\mathbb {Z}^d)\\ 0 \notin M, \, x\in M\\ \Lambda _M = \Lambda _k \end{array}} 12 \, \frac{(1 + \Vert {x}\Vert _\infty )^\nu (1+\textrm{diam}(M))^{d+1+2\nu }}{(1 + |{\Vert {x}\Vert _\infty - k - 1}| )^{d+1+\nu }}\Vert {\varPhi (M)}\Vert \, \Vert {A - \mathbb {E}_{\Lambda _k}A }\Vert _\nu \\&\le \sum _{x\in \mathbb {Z}^d} \sum _{k=0}^\infty 24 \, \frac{(1 + \Vert {x}\Vert _\infty )^\nu }{(1 + |{\Vert {x}\Vert _\infty - k - 1}| )^{d+1+\nu }} \, \Vert {\varPhi }\Vert _{d+1+2\nu } \, \frac{1}{(1+k)^{d+3+\nu }} \, \Vert {A}\Vert _{d+3+2\nu }\\&\le \sum _{x\in \mathbb {Z}^d} \sum _{k=0}^\infty 24 \, \frac{(1 + \Vert {x}\Vert _\infty )^\nu }{(1 + |{\Vert {x}\Vert _\infty - 1}| )^{d+1+\nu }} \, \Vert {\varPhi }\Vert _{d+1+2\nu } \, \frac{1}{(1+k)^{2}} \, \Vert {A}\Vert _{d+3+2\nu } \, , \end{aligned}$$

which is finite. In the first step of the above estimate we used that $(1+\textrm{diam}(M))^{d+1+2\nu } \Vert {\varPhi (M)}\Vert \le \Vert {\varPhi }\Vert _{d+1+2\nu }$ and $\Vert {A - \mathbb {E}_{\Lambda _k}A }\Vert _\nu \le \frac{2}{(1+k)^{d+3+\nu }} \, \Vert {A}\Vert _{d+3+2\nu }$. The second estimate is obtained as follows:

$$\begin{aligned}&\Vert {A - \mathbb {E}_{\Lambda _k}A }\Vert _\nu \, (1+k)^{d+3+\nu }\\&\quad = \Vert {A - \mathbb {E}_{\Lambda _k}A }\Vert \, (1+k)^{d+3+\nu } + \sup _{l\in \mathbb {N}_0} \Vert {(1-\mathbb {E}_{\Lambda _l})(1-\mathbb {E}_{\Lambda _k})A}\Vert (1+l)^\nu (1+k)^{d+3+\nu }\\&\quad = \Vert {A - \mathbb {E}_{\Lambda _k}A }\Vert \, (1+k)^{d+3+\nu } + \sup _{l\in \mathbb {N}_0} \Vert {(1-\mathbb {E}_{\Lambda _{\max (l,k)}})A}\Vert (1+l)^\nu (1+k)^{d+3+\nu }\\&\quad = \Vert {A - \mathbb {E}_{\Lambda _k}A }\Vert \, (1+k)^{d+3+\nu } + \sup _{\begin{array}{c} l\in \mathbb {N}_0\\ l \ge k \end{array}} \Vert {(1-\mathbb {E}_{\Lambda _{l}})A}\Vert (1+l)^\nu (1+k)^{d+3+\nu }\\&\quad \le \Vert {A - \mathbb {E}_{\Lambda _k}A }\Vert \, (1+k)^{d+3+\nu } + \sup _{\begin{array}{c} l\in \mathbb {N}_0\\ l \ge k \end{array}} \Vert {(1-\mathbb {E}_{\Lambda _{l}})A}\Vert (1+l)^{d+3+2\nu }\\&\quad \le 2 \Vert {A}\Vert _{d+3+2\nu }\,. \end{aligned}$$

We proceed similarly for the sum corresponding to the position operator

$$\begin{aligned} \sum _{x\in \mathbb {Z}^d} \Vert {[{x_j\,n_x, A}]}\Vert _\nu&= \sum _{x\in \mathbb {Z}^d} \Vert {[{x_j\,n_x, A -\mathbb {E}_{\Lambda _{(\Vert {x}\Vert _\infty -1)}}A }]}\Vert _\nu \\  &\le \sum _{x\in \mathbb {Z}^{d}} 4 |{x_j}| \,\Vert {n_x}\Vert _\nu \,\Vert {A -\mathbb {E}_{\Lambda _{(\Vert {x}\Vert _\infty -1)}}A }\Vert _\nu \,. \end{aligned}$$

Using that $\Vert {n_x}\Vert _\nu \le 3 \, \Vert {n_0}\Vert \left( 1+ \left( \Vert {x}\Vert _\infty - 1 \right) \right) ^\nu$ together with the same estimate as above we get

$$\begin{aligned} \sum _{x\in \mathbb {Z}^d} \Vert {[{x_j\,n_x, A}]}\Vert _\nu \le \sum _{x\in \mathbb {Z}^d} 24 \, |{x_j}| \,\Vert {n_0}\Vert \, \frac{(1+ |{\Vert {x}\Vert _\infty - 1}|)^{\nu }}{(1+ |{\Vert {x}\Vert _\infty - 1}|)^{d+2+\nu }} \Vert {A}\Vert _{d+2+2\nu }\,, \end{aligned}$$

which is again finite. Thus, we see that both sums converge absolutely and that the limits lie in $D_\infty$. Further we observe that for $k\in \mathbb {N}_0$

$$\begin{aligned}&\Vert {\sum _{M\in P_0(\mathbb {Z}^d)} [(p\varPhi + q X_j)(M),A] - \mathcal {L}_{p\varPhi + q X_j}^\circ ( \mathbb {E}_{\Lambda _k} A)}\Vert \\  &\quad \le \sum _{M\in P_0(\mathbb {Z}^d)} \Vert { [(p\varPhi + q X_j)(M),A - \mathbb {E}_{\Lambda _k} A]}\Vert _0 \, . \end{aligned}$$

By the above estimates and the second property from Lemma 2.3 the expression converges to 0 as $k\rightarrow \infty$. Since $\mathcal {L}_{p\varPhi + q X_j}$ is the closure of $\mathcal {L}_{p\varPhi + q X_j}^\circ$, we can conclude that $A\in D(\mathcal {L}_{p\varPhi + q X_j})$ and

$$\begin{aligned} \begin{aligned} \mathcal {L}_{p\varPhi + q X_j} A=&\sum _{M\in P_0(\mathbb {Z}^d)} [(p\varPhi + q X_j)(M),A] \\ =&\;p \sum _{M\in P_0(\mathbb {Z}^d)} [\varPhi (M),A] + q \sum _{x\in \mathbb {Z}^d} [x_j n_x, A] \,. \end{aligned} \end{aligned}$$

Combining the three estimates results in a constant $c_\nu$ such that

$$\begin{aligned} \Vert {\mathcal {L}_{p\varPhi + q X_j} A}\Vert _\nu \le c_\nu \, (p \,\Vert {\varPhi }\Vert _{d+1+2\nu } + q \,\Vert {n_0}\Vert ) \, \Vert {A}\Vert _{d+3+2\nu }. \end{aligned}$$

$$\square $$

### Proof of Lemma 2.6

The existence of a unique generated cocycle under the conditions in Lemma 2.6 is proven in [15, Corollary 5.2] together with the following Lieb–Robinson Bound. There exists a constant $\tilde{c}_{\nu }$ such that for all $\Lambda _1, \Lambda _2 \in P_0(\mathbb {Z}^d)$ and $A\in \mathcal {A}_{\Lambda _1}^N$, $B\in \mathcal {A}_{\Lambda _2}$ it holds that

$$\begin{aligned} \Vert {[\alpha _{u,v}A,B]}\Vert&\le 2 \tilde{c}_{\nu }^{-1} \left( \exp \!\Big (2\tilde{c}_{\nu } \, \sup _{t\in \mathbb {R}}\, \Vert {\varPhi ^t}\Vert _{2d+1+\nu } \, |u-v|\Big ) -1\right) \, \\  &\quad \Vert {A}\Vert \Vert {B}\Vert \sum _{x \in \Lambda _1} \sum _{y\in \Lambda _2} \frac{1}{(1+\Vert {x-y}\Vert _\infty )^{2d+1+\nu }}\, . \end{aligned}$$

For our purposes it is convenient to work with the weaker bound:

$$\begin{aligned} \Vert {[\alpha _{u,v}A,B]}\Vert&\le 2 \tilde{c}_{\nu }^{-1} \kappa _d \exp \!\Big (2\tilde{c}_{\nu } \, \sup _{t\in \mathbb {R}}\, \Vert {\varPhi ^t}\Vert _{2d+1+\nu } \, |u-v|\Big ) \, \\  &\quad \Vert {A}\Vert \Vert {B}\Vert |\Lambda _1| \, \frac{1}{(1+\textrm{dist}(\Lambda _1,\Lambda _2))^{d+\nu }}\, , \end{aligned}$$

where $\kappa _d = \sum _{y\in \mathbb {Z}^d} \frac{1}{(1+\Vert {y}\Vert _\infty )^{d+1}}$. To prove the desired bound on

$$\begin{aligned} \Vert {\alpha _{u,v} A }\Vert _\nu&= \Vert { A }\Vert + \sup _{k\in \mathbb {N}_0} \Vert {(1-\mathbb {E}_{\Lambda _k})\alpha _{u,v} A}\Vert (1+k)^\nu \, , \end{aligned}$$

we estimate the expression under the supremum as

$$\begin{aligned}&\Vert {(1-\mathbb {E}_{\Lambda _k})\alpha _{u,v} A}\Vert (1+k)^\nu \\  &\quad \le \Vert {(1-\mathbb {E}_{\Lambda _k})\alpha _{u,v} \mathbb {E}_{\Lambda _{\lfloor {\frac{k}{2}}\rfloor }}A}\Vert (1+k)^\nu + \Vert {(1-\mathbb {E}_{\Lambda _k})\alpha _{u,v} (1 - \mathbb {E}_{\Lambda _{\lfloor {\frac{k}{2}}\rfloor }})A}\Vert (1+k)^\nu \\&\quad \le \Vert {(1-\mathbb {E}_{\Lambda _k})\alpha _{u,v} \mathbb {E}_{\Lambda _{\lfloor {\frac{k}{2}}\rfloor }}A}\Vert (1+k)^\nu + 2\Vert {(1 - \mathbb {E}_{\Lambda _{\lfloor {\frac{k}{2}}\rfloor }}) A}\Vert (1+k)^\nu \\&\quad \le \Vert {(1-\mathbb {E}_{\Lambda _k})\alpha _{u,v} \mathbb {E}_{\Lambda _{\lfloor {\frac{k}{2}}\rfloor }}A}\Vert (1+k)^\nu + 2^{\nu +1} \Vert {A}\Vert _\nu \, . \end{aligned}$$

Lemma C.2 from [29] tells us that we can use our Lieb–Robinson Bound to get the following estimate:

$$\begin{aligned}&\Vert {(1-\mathbb {E}_{\Lambda _k})\alpha _{u,v} \mathbb {E}_{\Lambda _{\lfloor {\frac{k}{2}}\rfloor }}A}\Vert (1+k)^\nu \\  &\quad \le 2\tilde{c}_{\nu }^{-1} \kappa _d \exp \!\Big (2\tilde{c}_{\nu } \, \sup _{t\in \mathbb {R}}\, \Vert {\varPhi ^t}\Vert _{2d+1+\nu } \, |u-v|\Big ) \, \Vert {A}\Vert \,|\Lambda _{\lfloor {\frac{k}{2}}\rfloor }| \, \frac{(1+k)^\nu }{(1+\lfloor {\frac{k}{2}}\rfloor )^{\nu +d}}\\&\quad \le 2\tilde{c}_{\nu }^{-1} \kappa _d \exp \!\Big (2\tilde{c}_{\nu } \, \sup _{t\in \mathbb {R}}\, \Vert {\varPhi ^t}\Vert _{2d+1+\nu } \, |u-v|\Big ) \, \Vert {A}\Vert \, \frac{2^d(1+k)^\nu }{(1+\lfloor {\frac{k}{2}}\rfloor )^{\nu }} \\&\quad \le 2\tilde{c}_{\nu }^{-1} \kappa _d \exp \!\Big (2\tilde{c}_{\nu } \, \sup _{t\in \mathbb {R}}\, \Vert {\varPhi ^t}\Vert _{2d+1+\nu } \, |u-v|\Big ) \, \Vert {A}\Vert \, 2^{d+ \nu } \, . \end{aligned}$$

In total we have

$$\begin{aligned} \Vert {\alpha _{u,v} A }\Vert _\nu&\le \Vert { A }\Vert + 2^{d+\nu +1} \,\tilde{c}_{\nu }^{-1} \kappa _d \exp \!\Big (2\tilde{c}_{\nu } \, \sup _{t\in \mathbb {R}}\, \Vert {\varPhi ^t}\Vert _{2d+1+\nu } \, |u-v|\Big ) \, \Vert {A}\Vert + 2^{\nu +1} \Vert {A}\Vert _\nu \\&\le (1 + 2^{d+\nu + 1} \tilde{c}_{\nu }^{-1} \kappa _d + 2^{\nu +1} ) \exp \!\Big (2\tilde{c}_{\nu } \, \sup _{t\in \mathbb {R}}\, \Vert {\varPhi ^t}\Vert _{2d+1+\nu } \, |u-v|\Big ) \, \Vert {A}\Vert _\nu \end{aligned}$$

and defining $c_{\nu } = \max ((2^{d+\nu } \tilde{c}_{\nu }^{-1} \kappa _d + 2^{\nu +1} + 1), 2\tilde{c}_{\nu })$ gives us the desired bound. The bound for the case $\sup _{v\in \mathbb {R}} \Vert {\varPhi ^{v}}\Vert _{\exp ,a} < \infty$ can be found in Lemma B.3 of [28].

It remains to prove $\alpha _{u,v} D_\infty = D_\infty$. With the bound we have already shown and the fact that $\alpha _{u,v}^{-1} = \alpha _{v,u}$ this reduces to showing that $\alpha _{u,v} \mathcal {A}^N \subseteq \mathcal {A}^N$. To this end, let $\varphi \in \mathbb {R}$ and $A\in \mathcal {A}_0$. It holds that

$$\begin{aligned} -\textrm{i}\, \partial _v \, g_\varphi \alpha _{u,v}g_\varphi ^{-1} A&= g_\varphi \alpha _{u,v} \mathcal {L}_{\varPhi ^v} g_\varphi ^{-1} A \;=\; g_\varphi \alpha _{u,v} \sum _{M\in P_0(\mathbb {Z}^{d})} [\varPhi ^v(M),g_\varphi ^{-1} A]\\&= g_\varphi \alpha _{u,v} g_\varphi ^{-1} \sum _{M\in P_0(\mathbb {Z}^{d})} [\varPhi ^v(M), A] \;=\; g_\varphi \alpha _{u,v} g_\varphi ^{-1} \mathcal {L}_{\varPhi ^v} A\, . \end{aligned}$$

By the uniqueness of the cocycle generated by $(\varPhi ^{v})_{v\in \mathbb {R}}$ it follows that $g_\varphi \alpha _{u,v} g_\varphi ^{-1} = \alpha _{u,v}$ and therefore $\alpha _{u,v} \mathcal {A}^N \subseteq \mathcal {A}^N$. $\square$

### Proof of Lemma 2.8

We show that for all $M\subset \mathbb {Z}^d$ the map $T_\gamma ^{-1} \, \mathbb {E}_{M+\gamma } \, T_\gamma$ is equal to $\mathbb {E}_M$. Let $M\subseteq \mathbb {Z}^d$, $A \in \mathcal {A}$ and $B\in \mathcal {A}_M$ and consider

$$\begin{aligned} \omega ^\textrm{tr}\! \left( \left( T_\gamma ^{-1} \, \mathbb {E}_{M+\gamma } \, T_\gamma \, A\right) B\right) \, . \end{aligned}$$

The uniqueness of the tracial state implies that it must be invariant under all automorphisms, which we use to rewrite

Property $(\textrm{i})$ of the translation implies that $T_\gamma \, B \in \mathcal {A}_{M+\gamma }$, which allows us to use the defining property of the conditional expectation from Proposition 2.1 to get

$$\begin{aligned} \omega ^\textrm{tr}\! \left( \left( T_\gamma ^{-1} \, \mathbb {E}_{M+\gamma } \, T_\gamma \, A\right) B\right) = \omega ^\textrm{tr}\! \left( \left( T_\gamma \, A\right) \left( T_\gamma \, B\right) \right) = \omega ^\textrm{tr}\! \left( T_\gamma \left( A \, B \right) \right) = \omega ^\textrm{tr}\! \left( A \, B \right) \, . \end{aligned}$$

By the uniqueness from Proposition 2.1, we have shown .

For the second statement we show that the one-parameter group of automorphisms $\left( T_\gamma ^{-1} \, g_\varphi \, T_\gamma \right) _{\varphi \in \mathbb {R}}$ is generated by the number operator *N*. To this end let $A \in \mathcal {A}_M$ for some $M\in P_0(\mathbb {Z}^{d})$ and consider

$$\begin{aligned} -\textrm{i}\, \partial _\varphi \left( T_\gamma ^{-1} \, g_\varphi \, T_\gamma A \right) = T_\gamma ^{-1} g_\varphi \mathcal {L}_N T_\gamma A = \sum _{x\in M+\gamma } T_\gamma ^{-1} g_\varphi T_\gamma \, [ T_\gamma ^{-1} n_x, A]\, . \end{aligned}$$

With property (ii) of the translation, this gives us

$$\begin{aligned} -\textrm{i}\,\partial _\varphi \left( T_\gamma ^{-1} \, g_\varphi \, T_\gamma A \right) = \sum _{x\in M+\gamma } T_\gamma ^{-1} g_\varphi T_\gamma \,[ n_{x-\gamma }, A] = T_\gamma ^{-1} g_\varphi T_\gamma \,\mathcal {L}_N A \, . \end{aligned}$$

Therefore, $\left( T_\gamma ^{-1} \, g_\varphi \, T_\gamma \right) _{\varphi \in \mathbb {R}}$ is generated by *N* and by the uniqueness from Lemma 2.6, we have $T_\gamma ^{-1} \, g_\varphi \, T_\gamma = g_\varphi$ for all $\varphi \in \mathbb {R}$. $\square$

### Proof of Proposition 2.11

Since translations and the conditional expectation preserve gauge-invariance and self-adjointness (see Proposition 2.1 and Lemma 2.8), we see that $\varPhi _A^T(M)$ is self-adjoint and gauge-invariant for all $M\in P_0(\mathbb {Z}^d)$. Next we check the *T*-periodicity: Let $k\in \mathbb {N}_0$, $\gamma ,\mu \in \mathbb {Z}^d$. Due to the *T*-compatibility of *A* and Lemma 2.8, it holds that

$$\begin{aligned} T_\gamma \, T_\mu \, \mathbb {E}_{\Lambda _k} \, A&= \mathbb {E}_{\Lambda _k + \mu + \gamma }\, T_\gamma \, T_\mu \, A\\&= \mathbb {E}_{\Lambda _k + \mu + \gamma }\, T_{\gamma + \mu } \, A\\&= T_{\gamma + \mu } \, \mathbb {E}_{\Lambda _k }\, A\, , \end{aligned}$$

which implies the *T*-periodicity of $\varPhi _A^T$. We show that for each $\nu \in \mathbb {N}_0$ the interaction norm $\Vert \varPhi _A^T \Vert _\nu$ is finite: Let $\nu \in \mathbb {N}_0$. By the definition of the norm and $\varPhi _A$, we have that

$$\begin{aligned} \Vert \varPhi _A^T \Vert _\nu&= \sup _{x\in \mathbb {Z}^d} \sum _{\begin{array}{c} M\in P_0(\mathbb {Z}^d)\\ x\in M \end{array}} (1+\textrm{diam}(M))^\nu \, \Vert \varPhi _A^T(M) \Vert \\&= \sum _{k=1}^\infty \sum _{\gamma \in \Lambda _k} (1+2\, k)^\nu \, \Vert T_{-\gamma }\,(\mathbb {E}_{\Lambda _k}\, A - \mathbb {E}_{\Lambda _{k-1}}\, A )\Vert + \Vert \mathbb {E}_{\Lambda _0}\, A \Vert \, . \end{aligned}$$

Using properties of the conditional expectation from Proposition 2.1 and the definition of the decay norm, we arrive at

$$\begin{aligned} \Vert \varPhi _A^T \Vert _\nu&\le \sum _{k=1}^\infty \frac{(1 + 2\, k )^d\,(1+2\, k)^\nu }{(1 + (k-1))^{d+\nu +2}} \, \Vert A \Vert _{\nu +d+2} + \Vert \mathbb {E}_{\Lambda _0}\, A \Vert < \infty \, . \end{aligned}$$

We show that $(\varPhi _A^T)_0 = A$. Since $\varPhi _A^T$ is supported only on the boxes $\Lambda _k + \gamma$ for $k\in \mathbb {N}_0$ and $\gamma \in \mathbb {Z}^d$ and the representative at 0 of such a box is $\Lambda _k$, we get

$$\begin{aligned} (\varPhi _A^T)_0&= \sum _{M\in R_0(\mathbb {Z}^d)}\varPhi _A^T(M) \\&= \sum _{k=1}^\infty (\mathbb {E}_{\Lambda _k} \, A - \mathbb {E}_{\Lambda _{k-1}} \, A) + \mathbb {E}_{\Lambda _0}\, A\\&= \lim _{k\rightarrow \infty } \mathbb {E}_{\Lambda _k} \, A = A \,. \end{aligned}$$

The limit in the last line converges to *A* as shown in Lemma 2.3.

Now let $\varPhi$ be a *T*-periodic $B_\infty$-interaction. For $\nu \in \mathbb {N}_0$ it holds that

$$\begin{aligned} \sum _{M \in R_0(\mathbb {Z}^d)} \Vert {\varPhi (M)}\Vert _\nu&\le \sum _{M \in R_0(\mathbb {Z}^d)} 3 \, (1 + \textrm{diam}(M))^\nu \Vert {\varPhi (M)}\Vert \, , \end{aligned}$$

since each *M* is a standard representative and thus contained in a box around 0 of side length $2\, \textrm{diam}(M)$. By periodicity, we can shift each *M* such that it contains 0, giving us the estimate

17 $$\begin{aligned} \sum _{M \in R_0(\mathbb {Z}^d)} \Vert {\varPhi (M)}\Vert _\nu \le \sum _{\begin{array}{c} M \in P_0(\mathbb {Z}^d)\\ 0\in M \end{array}} 3\, \left( 1+ \textrm{diam}(M)\right) ^\nu \Vert {\varPhi (M)}\Vert \le 3\Vert {\varPhi }\Vert _\nu < \infty \, . \end{aligned}$$

Therefore, $\varPhi$ is absolutely summable at 0 and $\Vert {\varPhi _0}\Vert _\nu < \infty$. Since all local terms of $\varPhi$ are gauge-invariant and self-adjoint we get that $\varPhi _0$ lies in $D_\infty$ and is self-adjoint. It remains to show the *T*-compatibility of $\varPhi _0$. Let $\gamma , \mu \in \mathbb {Z}^d$ and consider

$$\begin{aligned} T_{\gamma + \mu } \, \varPhi _0&= \sum _{M\in R_0(\mathbb {Z}^{d})} T_{\gamma + \mu } \, \varPhi (M)\\  &= \sum _{M\in R_0(\mathbb {Z}^{d})} \varPhi (M + \gamma + \mu )= \sum _{M\in R_0(\mathbb {Z}^{d})} T_{\gamma } \, T_\mu \, \varPhi (M)= T_{\gamma } \, T_\mu \, \varPhi _0 \, . \end{aligned}$$

$$\square $$

### Proof of Proposition 2.12

Let $A \in D_\infty$. Lemma 2.5 tells us that

$$\begin{aligned} \mathcal {L}_\varPhi A = \sum _{M \in P_0(\mathbb {Z}^d)} [\varPhi (M),A]\,, \end{aligned}$$

where the sum converges absolutely. We can thus rearrange the sum and use the fact that $\varPhi$ is absolutely summable at 0 from Proposition 2.11, to get

$$\begin{aligned} \mathcal {L}_\varPhi A = \sum _{\gamma \in \mathbb {Z}^d} \sum _{M \in R_0(\mathbb {Z}^d)} [T_\gamma \varPhi (M),A]&= \sum _{\gamma \in \mathbb {Z}^d} [T_\gamma \sum _{M \in R_0(\mathbb {Z}^d)} \varPhi (M),A] = \sum _{\gamma \in \mathbb {Z}^d} [T_\gamma \varPhi _0,A] \, . \end{aligned}$$

For the proof of (ii), for each $M \in P_0(\mathbb {Z}^d)$ and $k\in \mathbb {N}_0$ we define

$$\begin{aligned} n(M,k) = |\{\gamma \in \mathbb {Z}^d~|~ M+\gamma \subseteq \Lambda _k\}| \, . \end{aligned}$$

Let *M*, $m\in \mathbb {N}$ be such that $M\subseteq \Lambda _m$. We find that for all $k\in \mathbb {N}$ with $k\ge m$

$$\begin{aligned} (2(k-m)+1)^d = n(\Lambda _m,k) \le n(M,k) \le |\Lambda _k| = (2k+1)^d~, \end{aligned}$$

thus

$$\begin{aligned} 0 \le \frac{n(M,k)}{|\Lambda _k|} \le 1 \qquad \text{ and }\qquad \lim _{k\rightarrow \infty } \frac{n(M,k)}{|\Lambda _k|} = 1 \, . \end{aligned}$$

With this we can calculate as follows

$$\begin{aligned}&\frac{1}{|\Lambda _k|}\sum _{M\subseteq \Lambda _k} \omega (\varPhi (M)) = \frac{1}{|\Lambda _k|}\sum _{M\in R_0(\mathbb {Z}^d)} \sum _{\gamma \in \mathbb {Z}^d} \chi _{(M+\gamma )\subseteq \Lambda _k} ~ \omega (\varPhi (M + \gamma ))\\&\quad = \frac{1}{|\Lambda _k|}\sum _{M\in R_0(\mathbb {Z}^d)} \sum _{\gamma \in \mathbb {Z}^d} \chi _{(M+\gamma )\subseteq \Lambda _k} ~ \omega (T_{\gamma }\varPhi (M))\\  &\quad = \frac{1}{|\Lambda _k|}\sum _{M\in R_0(\mathbb {Z}^d)} \sum _{\gamma \in \mathbb {Z}^d} \chi _{(M+\gamma )\subseteq \Lambda _k} ~ \omega (\varPhi (M))\\&\quad = \sum _{M\in R_0(\mathbb {Z}^d)} \frac{n(M,k)}{|\Lambda _k|} ~ \omega (\varPhi (M)) ~. \end{aligned}$$

Using dominated convergence and continuity of $\omega$ we arrive at our desired result. $\square$

### Proof of Proposition 2.13

To show that $\textrm{i}[\varPhi , \varPsi ]$ is *T*-periodic, let $\gamma \in \mathbb {Z}^d$ and $M\in P_0(\mathbb {Z}^d)$. We have

$$\begin{aligned} \begin{aligned} \text {i}[\varPhi , \varPsi ](M+\gamma )&= \sum _{\begin{array}{c} M_1,M_2 \subset (M + \gamma )\\ M_1 \cup M_2= (M+\gamma ) \end{array}} \text {i}[\varPhi (M_1),\varPsi (M_2)] \\  &= \sum _{\begin{array}{c} M_1,M_2 \subset M \\ M_1 \cup M_2 = M \end{array}} \text {i}[\varPhi (M_1+\gamma ),\varPsi (M_2+\gamma )]~. \end{aligned} \end{aligned}$$

By the *T*-periodicity of $\varPhi$ and $\varPsi$ we get

$$\begin{aligned} \textrm{i}[\varPhi , \varPsi ](M+\gamma )&= \sum _{\begin{array}{c} M_1,M_2 \subset M \\ M_1 \cup M_2 = M \end{array}} \textrm{i}[T_\gamma \varPhi (M_1),T_\gamma \varPsi (M_2)] \\  &\quad = T_\gamma \sum _{\begin{array}{c} M_1,M_2 \subset M \\ M_1 \cup M_2 = M \end{array}} \textrm{i}[\varPhi (M_1),\varPsi (M_2)] = T_\gamma \textrm{i}[\varPhi , \varPsi ](M)~, \end{aligned}$$

showing that $\textrm{i}[\varPhi , \varPsi ]$ is *T*-periodic. To show that $\textrm{i}[\varPhi , \varPsi ] \in B_\infty$ let $\nu \in \mathbb {N}_0$ and consider

18 $$\begin{aligned} \Vert {\textrm{i}[\varPhi , \varPsi ]}\Vert _\nu&= \sup _{x\in \mathbb {Z}^d} \sum _{\begin{array}{c} M\in P_0(\mathbb {Z}^d)\\ x \in M \end{array}} (1+\textrm{diam}(M))^\nu \Vert {\textrm{i}[\varPhi , \varPsi ](M)}\Vert \nonumber \\&\le \sup _{x\in \mathbb {Z}^d} \sum _{\begin{array}{c} M\in P_0(\mathbb {Z}^d)\\ x \in M \end{array}} \sum _{\begin{array}{c} M_1,M_2 \subset M \\ M_1 \cup M_2 = M \end{array}} (1+\textrm{diam}(M))^\nu \Vert {[\varPhi (M_1),\varPsi (M_2)]}\Vert \nonumber \\&\le \sup _{x\in \mathbb {Z}^2} \sum _{\begin{array}{c} M\in P_0(\mathbb {Z}^d)\\ x \in M \end{array}} \sum _{\begin{array}{c} M_1,M_2 \subset M \\ M_1 \cup M_2 = M \\ M_1 \cap M_2 \ne \emptyset \end{array}} (1+\textrm{diam}(M))^\nu 2\Vert {\varPhi (M_1)}\Vert \Vert {\varPsi (M_2)}\Vert \end{aligned}$$

From here one can change the summation index and then further estimate to get

$$\begin{aligned} \begin{aligned} \Vert {\text {i}[\varPhi , \varPsi ]}\Vert _{\nu }\le \;&\sup _{x\in \mathbb {Z}^d} \sum _{\begin{array}{c} M_1,M_2 \in P_0(\mathbb {Z}^d)\\ x \in M_1 \cup M_2 \\ M_1 \cap M_2 \ne \emptyset \end{array}} (1+\text {diam}(M_1 \cup M_2))^\nu 2\Vert {\varPhi (M_1)}\Vert \Vert {\varPsi (M_2)}\Vert \\\le \;&\sup _{x\in \mathbb {Z}^{d}} \sum _{\begin{array}{c} M_1 \in P_0(\mathbb {Z}^{d})\\ x \in M_1 \end{array}} \sum _{\begin{array}{c} M_2 \in P_0(\mathbb {Z}^d) \\ M_1 \cap M_2 \ne \emptyset \end{array}} (1+\text {diam}(M_1 \cup M_2))^\nu 2\Vert {\varPhi (M_1)}\Vert \Vert {\varPsi (M_2)}\Vert \\  &+ \sup _{x\in \mathbb {Z}^d} \sum _{\begin{array}{c} M_2 \in P_0(\mathbb {Z}^{d})\\ x \in M_2 \end{array}} \sum _{\begin{array}{c} M_1 \in P_0(\mathbb {Z}^{d})\\ M_1 \cap M_2 \ne \emptyset \end{array}} (1+\text {diam}(M_1 \cup M_2))^\nu 2\Vert {\varPhi (M_1)}\Vert \Vert {\varPsi (M_2)}\Vert \\ =:\;&S_1 + S_2 ~. \end{aligned} \end{aligned}$$

We proceed by treating each summand separately. For $S_1$ we find

$$\begin{aligned}S_1&\le \sup _{x\in \mathbb {Z}^d} \sum _{\begin{array}{c} M_1 \in P_0(\mathbb {Z}^d)\\ x \in M_1 \end{array}} \sum _{y \in M_1 } \sum _{\begin{array}{c} M_2 \in P_0(\mathbb {Z}^d) \\ y\in M_2 \end{array}} (1+\text {diam}(M_1 \cup M_2))^\nu 2\Vert {\varPhi (M_1)}\Vert \Vert {\varPsi (M_2)}\Vert \\  &\le \sup _{x\in \mathbb {Z}^d} \sum _{\begin{array}{c} M_1 \in P_0(\mathbb {Z}^d)\\ x \in M_1 \end{array}} \\  &\quad \sum _{y \in M_1 } \sum _{\begin{array}{c} M_2 \in P_0(\mathbb {Z}^d) \\ y\in M_2 \end{array}} (1+\text {diam}(M_1))^\nu (1+\text {diam}(M_2))^\nu 2\Vert {\varPhi (M_1)}\Vert \Vert {\varPsi (M_2)}\Vert \\  &\le 2\sup _{x\in \mathbb {Z}^d} \sum _{\begin{array}{c} M_1 \in P_0(\mathbb {Z}^d)\\ x \in M_1 \end{array}} \\  &\quad (1+\text {diam}(M_1))^\nu \Vert {\varPhi (M_1)}\Vert \sum _{y \in M_1 } \sum _{\begin{array}{c} M_2 \in P_0(\mathbb {Z}^d) \\ y\in M_2 \end{array}} (1+\text {diam}(M_2))^\nu \Vert {\varPsi (M_2)}\Vert \\  &\le 2\sup _{x\in \mathbb {Z}^d} \sum _{\begin{array}{c} M_1 \in P_0(\mathbb {Z}^d)\\ x \in M_1 \end{array}} (1+\text {diam}(M_1))^\nu \Vert {\varPhi (M_1)}\Vert |M_1| \\  &\quad \sup _{y \in \mathbb {Z}^d }\sum _{\begin{array}{c} M_2 \in P_0(\mathbb {Z}^d) \\ y\in M_2 \end{array}} (1+\text {diam}(M_2))^\nu \Vert {\varPsi (M_2)}\Vert ~. \end{aligned}$$

We use that on the *d*-dimensional square lattice $|M_1| \le (2 \, \textrm{diam}(M_1) + 1)^d$ and periodicity of $\varPhi$ and $\varPsi$ to see

$$\begin{aligned} S_1&\le 2^{d+1} \sup _{x\in \mathbb {Z}^d} \sum _{\begin{array}{c} M_1 \in P_0(\mathbb {Z}^d)\\ x \in M_1 \end{array}} (1+\textrm{diam}(M_1))^{\nu +d} \Vert {\varPhi (M_1)}\Vert \\  &\quad \sup _{y \in \mathbb {Z}^d } \sum _{\begin{array}{c} M_2 \in P_0(\mathbb {Z}^d) \\ y\in M_2 \end{array}} (1+\textrm{diam}(M_2))^\nu \Vert {\varPsi (M_2)}\Vert \\&= 2^{d+1} \sum _{\begin{array}{c} M_1 \in P_0(\mathbb {Z}^d)\\ 0 \in M_1 \end{array}} (1+\textrm{diam}(M_1))^{\nu +d} \Vert {\varPhi (M_1)}\Vert \\  &\quad \sum _{\begin{array}{c} M_2 \in P_0(\mathbb {Z}^d) \\ 0\in M_2 \end{array}} (1+\textrm{diam}(M_2))^\nu \Vert {\varPsi (M_2)}\Vert ~. \end{aligned}$$

From which we can see that

$$\begin{aligned} S_1&\le 2 ^{d+1} \,\Vert {\varPhi }\Vert _{\nu +d} \,\Vert {\varPsi }\Vert _{\nu } \, < \, \infty \, . \end{aligned}$$

An analogous calculation shows that $S_2 < \infty$, hence $\textrm{i}[\varPhi ,\varPsi ] \in B_\infty$.

We prove the same statements for $\textrm{i}[X_j,\varPsi ]$. Let $\gamma \in \mathbb {Z}^d$ and $M\in P_0(\mathbb {Z}^d)$. To prove *T*-periodicity we consider

$$\begin{aligned} \textrm{i}[X_j, \varPsi ](M + \gamma ) = \sum _{\begin{array}{c} M_1,M_2 \subset M + \gamma \\ M_1 \cup M_2= M+\gamma \end{array}} \textrm{i}[X_j(M_1),\varPsi (M_2)] \end{aligned}$$

and see that since $X_j$ is only supported on one-element sets, the expression reduces to

$$\begin{aligned} \textrm{i}[X_j, \varPsi ](M + \gamma ) = \sum _{\begin{array}{c} x \in M + \gamma \end{array}} \textrm{i}[x_j n_x, \varPsi (M + \gamma )] =\sum _{\begin{array}{c} x \in M \end{array}} \textrm{i}[(x+\gamma )_j n_{x+\gamma }, \varPsi (M + \gamma )]~. \end{aligned}$$

From there we calculate

$$\begin{aligned} \textrm{i}[X_j, \varPsi ](M + \gamma )&=\sum _{\begin{array}{c} x \in M \end{array}} \textrm{i}[x_j n_{x+\gamma }, \varPsi (M + \gamma )] + \sum _{\begin{array}{c} x \in M \end{array}} \textrm{i}[\gamma _j n_{x+\gamma }, \varPsi (M + \gamma )]\\&=T_\gamma \sum _{\begin{array}{c} x \in M \end{array}} \textrm{i}[x_j n_{x}, \varPsi (M)] + T_\gamma \sum _{\begin{array}{c} x \in M \end{array}} \textrm{i}[\gamma _j n_{x}, \varPsi (M)]\\&=T_\gamma \textrm{i}[X_j, \varPsi ](M) + T_\gamma \gamma _j \textrm{i}\mathcal {L}_N(\varPsi (M)) \end{aligned}$$

and see, that since the terms of an interaction are always gauge-invariant, $\textrm{i}[X_j, \varPsi ]$ is *T*-periodic. Now let $\nu \in \mathbb {N}_0$. We look at

$$\begin{aligned} \Vert {\textrm{i}[X_j, \varPsi ]}\Vert _\nu&= \sup _{x\in \mathbb {Z}^d} \sum _{\begin{array}{c} M\in P_0(\mathbb {Z}^d)\\ x \in M \end{array}} (1+\textrm{diam}(M))^\nu \Vert {\mathcal {L}_{X_j}\varPsi (M)}\Vert {EMPTY}\\&= \sum _{\begin{array}{c} M\in P_0(\mathbb {Z}^d)\\ 0 \in M \end{array}} (1+\textrm{diam}(M))^\nu \Vert {\mathcal {L}_{X_j}\varPsi (M)}\Vert \end{aligned}$$

and use Lemma 2.5 (with $\nu =0$) to get the existence of a constant *c*, such that

$$\begin{aligned} \begin{aligned}\Vert {\text {i}[X_j, \varPsi ]}\Vert _\nu&\le \sum _{\begin{array}{c} M\in P_0(\mathbb {Z}^d)\\ 0 \in M \end{array}} (1+\text {diam}(M))^\nu c \Vert {n_0}\Vert \Vert {\varPsi (M)}\Vert _{d+3}\\  &\le \sum _{\begin{array}{c} M\in P_0(\mathbb {Z}^d)\\ 0 \in M \end{array}} 3\,(1+\text {diam}(M))^{\nu +d+3} c \Vert {\varPsi (M)}\Vert ~, \end{aligned} \end{aligned}$$

where we used the same estimate as in (16) in the last step. This expression is finite since $\varPsi \in B_\infty$. We observe that the property of being a *T*-periodic $B_\infty$-interaction is preserved when taking real linear combinations, hence we have shown statement (i).

For statement (ii) we proceed by using Lemma A.1 (with $\nu =0$ and $m= d+2$) on

$$\begin{aligned}&\sum _{M\in R_0(\mathbb {Z}^d)} \sum _{x\in \mathbb {Z}^d} \Vert {[x_j n_x, \varPsi (M)]}\Vert \le \sum _{M\in R_0(\mathbb {Z}^d)} \sum _{x\in \mathbb {Z}^d} 4^{d+5}\frac{|x_j| \Vert {n_0}\Vert _{d+2}}{(1+\Vert {x}\Vert _\infty )^{d+2}} \Vert { \varPsi (M)}\Vert _{d+2}\\&\quad \le \sum _{M\in R_0(\mathbb {Z}^d)} \sum _{x\in \mathbb {Z}^d} 4^{d+5}\frac{|x_j| \Vert {n_0}\Vert _{d+2}}{(1+\Vert {x}\Vert _\infty )^{d+2}} \,3\,(1+\textrm{diam}(M))^{d+2} \Vert { \varPsi (M)}\Vert \end{aligned}$$

and see that the expression is finite due to $\varPsi$ being a $B_\infty$-interaction. Hence, we can reorder the sum to see

$$\begin{aligned} \textrm{i}[X_j,\varPsi ]_0 = \sum _{M\in R_0(\mathbb {Z}^d)} \sum _{x\in \mathbb {Z}^d} \textrm{i}[x_j n_x, \varPsi (M)] = \sum _{x\in \mathbb {Z}^d} \sum _{M\in R_0(\mathbb {Z}^d)} \textrm{i}[x_j n_x, \varPsi (M)] = \textrm{i}\mathcal {L}_{X_j}\varPsi _0~. \end{aligned}$$

To prove statement (iii) we use Lemma A.1 twice to show absolute convergence of the sum

$$\begin{aligned}&\sum _{M\in P_0(\mathbb {Z}^d)} \sum _{x\in \mathbb {Z}^{d}} \Vert {[[x_j n_x,\varPsi (M)],A]}\Vert \\&\quad = \sum _{M\in R_0(\mathbb {Z}^d)} \sum _{\gamma \in \mathbb {Z}^d} \sum _{x\in \mathbb {Z}^d} \Vert {[[x_j n_x,T_\gamma \varPsi (M)],A]}\Vert \\&\quad = \sum _{M\in R_0(\mathbb {Z}^d)} \sum _{\gamma \in \mathbb {Z}^d} \sum _{x\in \mathbb {Z}^d} \Vert {[T_\gamma [x_j n_{x-\gamma }, \varPsi (M)],A]}\Vert \\&\quad \le \sum _{M\in R_0(\mathbb {Z}^d)} \sum _{\gamma \in \mathbb {Z}^d} \sum _{x\in \mathbb {Z}^d} 4^{2d+6}\frac{\Vert {A}\Vert _{2d+3}}{(1+\Vert {\gamma }\Vert _\infty )^{2d+3}}\Vert {[x_j n_{x-\gamma }, \varPsi (M)]}\Vert _{2d+3}\\&\quad \le \sum _{M\in R_0(\mathbb {Z}^d)} \sum _{\gamma \in \mathbb {Z}^d} \sum _{x\in \mathbb {Z}^d} 4^{5d+14}\frac{\Vert {A}\Vert _{2d+3}}{(1+\Vert {\gamma }\Vert _\infty )^{2d+3}} \frac{|x_j| \Vert {n_0}\Vert _{3d+5}}{(1+\Vert {x-\gamma }\Vert _\infty )^{d+2}} \Vert {\varPsi (M)}\Vert _{3d+5} \\&\quad \le \sum _{M\in R_0(\mathbb {Z}^d)} \sum _{\gamma \in \mathbb {Z}^d} \sum _{x\in \mathbb {Z}^d} 4^{5d+14}\frac{\Vert {A}\Vert _{2d+3}}{(1+\Vert {\gamma }\Vert _\infty )^{d+1}} \frac{|x_j| \Vert {n_0}\Vert _{3d+5}}{(1+\Vert {x}\Vert _\infty )^{d+2}} \Vert {\varPsi (M)}\Vert _{3d+5} \;<\;\infty ~. \end{aligned}$$

Therefore, we can again reorder the sum and get

$$\begin{aligned} \mathcal {L}_{\textrm{i}[\varPsi ,X_j]}A&= \sum _{M\in P_0(\mathbb {Z}^d)} \sum _{x\in \mathbb {Z}^d} \Big [\textrm{i}[\varPsi (M),x_j n_x],A\Big ] \\&= \sum _{x\in \mathbb {Z}^d} \sum _{M\in P_0(\mathbb {Z}^d)} \Big [\textrm{i}[\varPsi (M),x_j n_x],A\Big ] = \sum _{x \in \mathbb {Z}^d} [\textrm{i}\mathcal {L}_\varPsi x_j n_x ,A]~. \end{aligned}$$

Similarly we look at

$$\begin{aligned} \mathcal {L}_{\textrm{i}[\varPsi ,\varPhi ]} A = \sum _{\gamma \in \mathbb {Z}^d} \sum _{\begin{array}{c} M\in R_0(\mathbb {Z}^d) \end{array}} \sum _{\begin{array}{c} M_1,M_2 \subset M \\ M_1 \cup M_2 = M \end{array}} [\,T_\gamma \, \textrm{i}\, [\, \varPsi (M_1),\, \varPhi (M_2)\, ], \, A ]\, , \end{aligned}$$

which we will now show absolute convergence of. We first use Lemma A.1 to get

$$\begin{aligned}&\sum _{\gamma \in \mathbb {Z}^d} \sum _{\begin{array}{c} M\in R_0(\mathbb {Z}^d) \end{array}} \sum _{\begin{array}{c} M_1,M_2 \subset M \\ M_1 \cup M_2 = M \end{array}} \Vert [\,T_\gamma \, \textrm{i}\,[\, \varPsi (M_1),\, \varPhi (M_2)\, ], \, A ] \Vert \\&\quad \le \sum _{\gamma \in \mathbb {Z}^d} \sum _{\begin{array}{c} M\in R_0(\mathbb {Z}^d) \end{array}} \sum _{\begin{array}{c} M_1,M_2 \subset M \\ M_1 \cup M_2 = M \end{array}} 4^{d+4}\, \frac{\Vert [\, \varPsi (M_1),\, \varPhi (M_2)\, ] \Vert _{d+1} \, \Vert A \Vert _{d+1} }{(1+\Vert \gamma \Vert _\infty )^{d+1}} \end{aligned}$$

Observe that, by the same reasoning as in (17) we have

$$\begin{aligned}&\sum _{\begin{array}{c} M\in R_0(\mathbb {Z}^d) \end{array}} \sum _{\begin{array}{c} M_1,M_2 \subset M \\ M_1 \cup M_2 = M \end{array}} \Vert {[\varPhi (M_1),\varPsi (M_2)]}\Vert _{d+1}\\&\quad \le \sum _{\begin{array}{c} M\in P_0(\mathbb {Z}^d)\\ 0 \in M \end{array}} \sum _{\begin{array}{c} M_1,M_2 \subset M \\ M_1 \cup M_2 = M \end{array}} 3\,(1+ \textrm{diam}(M))^{d+1} \, \Vert {[\varPhi (M_1),\varPsi (M_2)]}\Vert \end{aligned}$$

and that therefore we have already shown when we proved statement (i), that the sum converges absolutely (see (18)). Hence, we can reorder the sum arbitrarily. We exploit this by performing a change in summation index via the bijection

$$\begin{aligned}&\{(M_1,M_2) | M_1\in P_0(\mathbb {Z}^d), \, M_2\in R_0(\mathbb {Z}^d) \} \rightarrow \{(M,M_1,M_2)| M\in R_0(\mathbb {Z}^d),\, \\&\quad M_1,M_2 \subset M,\, M_1 \cup M_2 = M\}\\&(M_1,M_2) \mapsto (M_1 \cup M_2 + s(M_1 \cup M_2),M_1 + s(M_1 \cup M_2), M_2 + s(M_1 \cup M_2)) ~, \end{aligned}$$

where $s(M_1 \cup M_2)$ is the shift vector defined in Eq. (6) and moving the left sum to the right. This gives us

$$\begin{aligned} \mathcal {L}_{\textrm{i}[\varPsi ,\varPhi ]} A&= \sum _{\begin{array}{c} M\in R_0(\mathbb {Z}^d) \end{array}} \sum _{\begin{array}{c} M_1,M_2 \subset M \\ M_1 \cup M_2 = M \end{array}} \sum _{\gamma \in \mathbb {Z}^d} [\,T_\gamma \, \textrm{i}\,[\, \varPsi (M_1),\, \varPhi (M_2)\, ], \, A ]\\&= \sum _{\begin{array}{c} M_1\in P_0(\mathbb {Z}^d) \end{array}} \sum _{\begin{array}{c} M_2 \in R_0(\mathbb {Z}^d) \end{array}} \sum _{\gamma \in \mathbb {Z}^d}\\&\quad [\, T_\gamma \,\textrm{i}[\varPsi (M_1 + s(M_1 \cup M_2)),\varPhi (M_2 +s(M_1 \cup M_2))], \, A\,]\, . \end{aligned}$$

Now we can exploit the periodicity of $\varPhi$ and $\varPsi$ together with an index shift to get

$$\begin{aligned} \mathcal {L}_{\textrm{i}[\varPsi ,\varPhi ]} A&= \sum _{\begin{array}{c} M_1\in P_0(\mathbb {Z}^d) \end{array}} \sum _{\begin{array}{c} M_2 \in R_0(\mathbb {Z}^d) \end{array}} \sum _{\gamma \in \mathbb {Z}^d} [\, T_{\gamma +s(M_1 \cup M_2)}\,\textrm{i}[\varPsi (M_1),\varPhi (M_2)], \, A\,]\\&= \sum _{\begin{array}{c} M_1\in P_0(\mathbb {Z}^d) \end{array}} \sum _{\begin{array}{c} M_2 \in R_0(\mathbb {Z}^d) \end{array}} \sum _{\gamma \in \mathbb {Z}^d} [\, T_{\gamma }\,\textrm{i}[\varPsi (M_1),\varPhi (M_2)], \, A\,]\, . \end{aligned}$$

Rearranging the sum again, we finally find

$$\begin{aligned} \mathcal {L}_{\textrm{i}[\varPsi ,\varPhi ]} A \&= \sum _{\gamma \in \mathbb {Z}^d} \sum _{\begin{array}{c} M_1\in P_0(\mathbb {Z}^d) \end{array}} \sum _{\begin{array}{c} M_2 \in R_0(\mathbb {Z}^d) \end{array}} [\, T_{\gamma }\,\textrm{i}[\varPsi (M_1),\varPhi (M_2)], \, A\,]\\&= \sum _{\gamma \in \mathbb {Z}^d} [\, T_\gamma \, \textrm{i}\, \mathcal {L}_{\varPsi } \, \varPhi _0, \, A\, ]\,. \end{aligned}$$

To prove statement (iv), we employ the same bijection to change the summation index:

$$\begin{aligned}&\omega (\textrm{i}[\varPhi ,\varPsi ]_0) = \omega (\sum _{\begin{array}{c} M\in R_0(\mathbb {Z}^d) \end{array}} \sum _{\begin{array}{c} M_1,M_2 \subset M \\ M_1 \cup M_2 = M \end{array}} \textrm{i}[\varPhi (M_1),\varPsi (M_2)]\,)\\&\quad = \omega (\sum _{\begin{array}{c} M_1\in P_0(\mathbb {Z}^d) \end{array}} \sum _{\begin{array}{c} M_2 \in R_0(\mathbb {Z}^d) \end{array}} \textrm{i}[\varPhi (M_1 + s(M_1 \cup M_2)),\varPsi (M_2 +s(M_1 \cup M_2))]\,)~. \end{aligned}$$

At this point we use the *T*-periodicity of $\omega$, $\varPhi$ and $\varPsi$ to see

$$\begin{aligned} \omega (\textrm{i}[\varPhi ,\varPsi ]_0)&= \omega (\sum _{\begin{array}{c} M_1\in P_0(\mathbb {Z}^d) \end{array}} \sum _{\begin{array}{c} M_2 \in R_0(\mathbb {Z}^d) \end{array}} T_{s(M_1 \cup M_2)} \textrm{i}[\varPhi (M_1),\varPsi (M_2 )]\,)\\&= \omega (\sum _{\begin{array}{c} M_1\in P_0(\mathbb {Z}^d) \end{array}} \sum _{\begin{array}{c} M_2 \in R_0(\mathbb {Z}^d) \end{array} }\textrm{i}[\varPhi (M_1),\varPsi (M_2 )]\,)\\&= \omega (\sum _{\begin{array}{c} M_1\in P_0(\mathbb {Z}^d) \end{array}} \textrm{i}\Big [\varPhi (M_1),\sum _{\begin{array}{c} M_2 \in R_0(\mathbb {Z}^d) \end{array}}\varPsi (M_2)\,\Big ]\,)\\&= \omega (\textrm{i}\mathcal {L}_\varPhi (\varPsi _0)) ~. \end{aligned}$$

By instead using the bijection

$$\begin{aligned}&\{(M_1,M_2) | M_1\in R_0(\mathbb {Z}^d), \, M_2\in P_0(\mathbb {Z}^d) \} \rightarrow \{(M,M_1,M_2)| \\  &\quad M\in R_0(\mathbb {Z}^d),\, M_1,M_2 \subset M,\, M_1 \cup M_2 = M\} \\&\quad (M_1,M_2) \mapsto (M_1 \cup M_2 + s(M_1 \cup M_2),M_1 + s(M_1 \cup M_2), M_2 + s(M_1 \cup M_2)) ~, \end{aligned}$$

one gets

$$\begin{aligned} \omega (\textrm{i}[\varPhi ,\varPsi ]_0) = -\omega (\textrm{i}\mathcal {L}_\varPsi (\varPhi _0)) ~. \end{aligned}$$

$$\square $$

## Appendix C: Proofs related to the Off-diagonal map

### Proof of Lemma 4.2

We need to show that $(\varPsi ^{\textrm{OD}\alpha })_*$ and $\mathcal {I}(\varPsi )_*$ are gauge-invariant, self-adjoint and that all of their decay norms are finite. The gauge-invariance and self-adjointness can easily be seen since $h_0$ lies in $D_\infty$ and is self-adjoint (Proposition 2.11) and all the maps $\alpha$, $\alpha ^{-1}$, $\textrm{e}^{\textrm{i}s\mathcal {L}_{H}}$ and $\textrm{i}\, \mathcal {L}_{\varPsi }$ send $D_\infty$ to itself (Lemmas 2.5 and 2.6) and preserve self-adjointness. For the finiteness of the decay norms, let $\nu \in \mathbb {N}_0$ and consider $\Vert {\alpha \mathcal {L}_{\varPsi } \alpha ^{-1} h_0}\Vert _\nu$, which is finite because $\alpha$, $\alpha ^{-1}$, and $\textrm{i}\, \mathcal {L}_{\varPsi }$ map $D_\infty$ to itself. By Lemma 2.6 we find that

$$\begin{aligned} \left\| \int _{\mathbb {R}}\textrm{d}s \,W_{g}(s)\, \textrm{e}^{\textrm{i}s\mathcal {L}_{H}} \alpha \textrm{i}\mathcal {L}_\varPsi \alpha ^{-1} h_0\right\| _\nu&\le \int _{\mathbb {R}}\textrm{d}s \Vert { \,W_g(s)\, \textrm{e}^{\textrm{i}s\mathcal {L}_{H}} \alpha \textrm{i}\mathcal {L}_\varPsi \alpha ^{-1} h_0}\Vert _\nu \\  &\le \int _{\mathbb {R}}\textrm{d}s \,|W_g(s)| \, b_{\nu }(|s|) \Vert { \alpha \textrm{i}\mathcal {L}_\varPsi \alpha ^{-1} h_0}\Vert _\nu < \infty \end{aligned}$$

and, since $\alpha ^{-1}$ maps $D_\infty$ to itself, we arrive at $(\varPsi ^{\textrm{OD}\alpha })_* \in D_\infty$. Analogously one finds that $\mathcal {I}(\varPsi )_* \in D_{\infty }$. To show that $(\varPsi ^{\textrm{OD}\alpha })_*$ is *T*-compatible i.e., that for $\gamma , \mu \in \mathbb {Z}^d$ we have $T_{\gamma + \mu } \, (\varPsi ^{\textrm{OD}\alpha })_* = T_\gamma \, T_\mu \, (\varPsi ^{\textrm{OD}\alpha })_*$, we observe that, since $\textrm{e}^{\textrm{i}s \mathcal {L}_H}$, $\alpha$ and $\alpha ^{-1}$ commute with the translation, we can pull $T_{\gamma + \mu }$ all the way through the expression to get

$$\begin{aligned} T_{\gamma + \mu } \, (\varPsi ^{\textrm{OD}\alpha })_*&= \alpha ^{-1} \int _{\mathbb {R}}\textrm{d}s \, W_g(s) \, \textrm{e}^{\textrm{i}s\mathcal {L}_{H}} \, \alpha \, \textrm{i}\, T_{\gamma + \mu } \, \mathcal {L}_\varPsi \, \alpha ^{-1} \, h_0\, . \end{aligned}$$

The position operator is periodic up to a shift proportional to the number operator, so for the last part of the expression we have

$$\begin{aligned} T_{\gamma + \mu } \mathcal {L}_{\varPsi } \alpha ^{-1} h_0&= (\mathcal {L}_{p Y} + \mathcal {L}_{qX_j} - q \,(\gamma + \mu )_j \, \mathcal {L}_{N}) T_{\gamma + \mu } \alpha ^{-1} h_0 \\&= (\mathcal {L}_{p Y} + \mathcal {L}_{q X_j} - q \,(\gamma + \mu )_j \, \mathcal {L}_{N}) \alpha ^{-1} T_{\gamma + \mu } h_0 \, . \end{aligned}$$

The periodicity of *H* implies that $h_0$ is *T*-compatible, as shown in Proposition 2.11. Moving $T_{\gamma } \, T_\mu$ all the way to the left of the expression with the same steps in reversed order produces

$$\begin{aligned} T_{\gamma + \mu } \, (\varPsi ^{\textrm{OD}\alpha })_* = T_{\gamma } \, T_\mu \, (\varPsi ^{\textrm{OD}\alpha })_* \, . \end{aligned}$$

One can proceed in the same way to show $T_{\gamma + \mu } \, \mathcal {I}(\varPsi )_* = T_\gamma \, T_\mu \, \mathcal {I}(\varPsi )_*$. $\square$

### Proof of Lemma 4.4

To prove the first claim, let $A\in D_\infty$ and consider $\mathcal {L}_{\varPsi ^{\textrm{OD}\alpha }} A$. Proposition 2.12 tells us that

$$\begin{aligned} \begin{aligned} \omega _\alpha (\mathcal {L}_{\varPsi ^{\text {OD}\alpha }}&A)= \sum _{\gamma \in \mathbb {Z}^d} \omega _\alpha ([T_\gamma (\varPsi ^{\text {OD}\alpha })_0 , A])\\  &= \sum _{\gamma \in \mathbb {Z}^d} \omega _\alpha \left( \Big [\text {i}\alpha ^{-1} \int _{\mathbb {R}}\text {d}s \,W_g(s) \,e^{\text {i}s\mathcal {L}_{H}} \alpha \mathcal {L}_\varPsi \alpha ^{-1} T_\gamma h_0 , \,A\,\Big ]\right) \\  &= \sum _{\gamma \in \mathbb {Z}^d} \int _{\mathbb {R}}\text {d}s \sum _{\mu \in \mathbb {Z}^d} \omega _0 \left( \Big [\text {i}W_g(s) \,e^{\text {i}s\mathcal {L}_{H}} [ \alpha \, (p \, T_\mu \, Y_0 + q \, \mu _j \, n_\mu ), T_\gamma h_0] ,\, \alpha \, A\, \Big ]\right) \, . \end{aligned} \end{aligned}$$

Similar to previous arguments we can show that this expression converges absolutely by using Lemmas A.1 and 2.6. Because of this, we can change the order of summation/integration to get

$$\begin{aligned}&\omega _\alpha (\mathcal {L}_{\varPsi ^{\textrm{OD}\alpha }} A) \\&\quad = \sum _{\mu \in \mathbb {Z}^d} \int _{\mathbb {R}}\textrm{d}s \sum _{\gamma \in \mathbb {Z}^d} \omega _0\left( \Big [\textrm{i}W_g(s)\, \textrm{e}^{\textrm{i}s\mathcal {L}_{H}} [ \alpha \, (p \, T_\mu \, Y_0 + q \, \mu _j \, n_\mu ), T_\gamma h_0] , \,\alpha \, A\,\Big ]\right) \\&\quad =\sum _{\mu \in \mathbb {Z}^d} \int _{\mathbb {R}}\textrm{d}s \, \omega _0\left( \Big [- \textrm{i}W_g(s) \,\textrm{e}^{\textrm{i}s\mathcal {L}_{H}} \mathcal {L}_H\,\alpha \, (p \, T_\mu \, Y_0 + q \, \mu _j \, n_\mu ) , \, \alpha \, A\,\Big ]\right) \\&\quad =\sum _{\mu \in \mathbb {Z}^d} \omega _0([-\textrm{i}\, \mathcal {L}_H \, \mathcal {I}_H \, \alpha \, (p \, T_\mu \, Y_0 + q \, \mu _j \, n_\mu ) , \, \alpha \, A]) \, , \end{aligned}$$

where we define $\mathcal {I}_H$ as in [28] as

$$\begin{aligned} \mathcal {I}_H \, A {:}{=}\int _\mathbb {R}\textrm{d}s\, W_g(s) \, \textrm{e}^{\textrm{i}s \mathcal {L}_H}\, A \end{aligned}$$

for $A \in D_\infty$. Now it follows from Proposition 3.3 in [28] that

$$\begin{aligned} \omega _0([-\textrm{i}\, \mathcal {L}_H \, \mathcal {I}_H \, \alpha \, (p \, T_\mu \, Y_0 + q \, \mu _j \, n_\mu ) , \alpha \, A]) = \omega _0([\alpha (p \, T_\mu \, Y_0 + q \, \mu _j \, n_\mu ) ,\, \alpha \, A])\, . \end{aligned}$$

Therefore by Proposition 2.12 it holds that

$$\begin{aligned} \omega _\alpha (\mathcal {L}_{\varPsi ^{\textrm{OD}\alpha }} A) = \sum _{\mu \in \mathbb {Z}^d} \omega _0([\alpha \, (p \, T_\mu \, Y_0 + q \, \mu _j \, n_\mu ) , \, \alpha \, A]) = \omega _\alpha (\mathcal {L}_\varPsi \, A)\, . \end{aligned}$$

For the proof of the second claim, let $A\in D_\infty$ and consider

$$\begin{aligned} \mathcal {L}_{-\textrm{i}[H,\mathcal {I}(\varPsi )]}\, A = - \sum _{\gamma \in \mathbb {Z}^d} [\, T_\gamma \, \textrm{i}\,\mathcal {L}_H \, \mathcal {I}(\varPsi )_0\, ,\, A \, ]\, . \end{aligned}$$

This identity holds by Proposition 2.13. By Propositions 2.12 and 2.11 it holds that

$$\begin{aligned} \textrm{i}\, \mathcal {L}_H \, \mathcal {I}(\varPsi )_0 = - \sum _{\gamma \in \mathbb {Z}^d} \int _\mathbb {R}\textrm{d}s \int _0^s \textrm{d}u \, W_g(s) \, \textrm{i}\, [ \, T_\gamma \, h_0, \, \textrm{e}^{\textrm{i}u \mathcal {L}_H} \, \textrm{i}\, \mathcal {L}_\varPsi \, h_0 \, ]\, , \end{aligned}$$

which is absolutely summable/integrable by the bounds from Lemmas A.1 and 2.6. We can therefore change the order of summation and integration to get

$$\begin{aligned} \textrm{i}\, \mathcal {L}_H \, \mathcal {I}(\varPsi )_0&= - \int _\mathbb {R}\textrm{d}s \int _0^s \textrm{d}u \, W_g(s) \, \textrm{e}^{\textrm{i}u \mathcal {L}_H} \, \textrm{i}\,\mathcal {L}_H\, \textrm{i}\,\mathcal {L}_\varPsi \, h_0\\&= -\int _\mathbb {R}\textrm{d}s \, W_g(s) \, (\textrm{e}^{\textrm{i}s \mathcal {L}_H} -1 ) \, \textrm{i}\,\mathcal {L}_\varPsi \, h_0 \\&= - \int _\mathbb {R}\textrm{d}s \, W_g(s) \, \textrm{e}^{\textrm{i}s \mathcal {L}_H} \, \textrm{i}\,\mathcal {L}_\varPsi \, h_0 \\&= -(\varPsi ^{\textrm{OD}})_0\, , \end{aligned}$$

where we used the fact that $W_g$ is an odd function in the third equality. By Proposition 2.12, we have therefore shown that

$$\begin{aligned} \mathcal {L}_{-\textrm{i}[H,\mathcal {I}(\varPsi )]}\, A = \sum _{\gamma \in \mathbb {Z}^d} [\, T_\gamma \, (\varPsi ^{\textrm{OD}})_0\, ,\, A \, ] = \mathcal {L}_{\varPsi ^{\textrm{OD}}}\, A \, . \end{aligned}$$

$$\square $$

### Proof of Lemma 4.5

Let $\nu \in \mathbb {N}_0$. We estimate

$$\begin{aligned} \sup _{\varepsilon \in [-1,1]} \Vert {(X_j^{\textrm{OD}\varepsilon })_0}\Vert _\nu&\le \sup _{\varepsilon \in [-1,1]} \int _{\mathbb {R}}\textrm{d}s \, |W_g(s)|\, \Vert {\beta _\varepsilon ^{-1} \, \textrm{e}^{\textrm{i}s\mathcal {L}_{H}} \beta _\varepsilon \mathcal {L}_{X_j} \beta _\varepsilon ^{-1} h_0}\Vert _\nu \end{aligned}$$

and use the bounds from Lemma 2.6 repeatedly for each of the automorphisms together with the bound from Lemma 2.5 to get constants $c_1$, $c_2$, $c_3$ and an at most polynomially growing function $b_\nu$, such that

$$\begin{aligned}&\sup _{\varepsilon \in [-1,1]} \Vert {(X_j^{\textrm{OD}\varepsilon })_0}\Vert _\nu \\&\quad \le \sup _{\varepsilon \in [-1,1]} \int _{\mathbb {R}}\textrm{d}s \, |{W_g(s)}| \, c_1^2 \, \exp \!\Big (2 \, c_1 \, \Vert {S_\varepsilon }\Vert _{2d+1+\nu }\Big ) \, b_\nu ({|{s}|}) \, c_2\,\Vert {n_0}\Vert \, c_3\\  &\qquad \exp \!\Big ( c_3 \, \Vert { S_\varepsilon }\Vert _{3d+4+2\nu } \Big ) \Vert {h_0}\Vert _{d+3+2\nu } \,. \end{aligned}$$

Due to definition of a gapped periodic system $\Vert {S_\varepsilon }\Vert _{2d+1+2\nu }$ and $\Vert {S_\varepsilon }\Vert _{3d+4+2\nu }$ are bounded in $\varepsilon$ and with the fact that $|W_g(s)|$ decays faster than any polynomial power of |*s*| as $|s| \rightarrow \infty$, this expression is finite. $\square$

## Appendix D: Proof of Lemma 5.2

If *u* is equal to *v* the identity holds. The proof strategy is to show that the left-hand side is differentiable with respect to *v* and that the derivative is zero everywhere.

By Propositions 2.12, 2.13, and Lemma 4.4 we can rewrite the left-hand side as

$$\begin{aligned} \overline{\omega _{\alpha _{u,v}}}(\textrm{i}[ X_1^{\textrm{OD}\alpha _{u,v}},X_j^{\textrm{OD}\alpha _{u,v}}])&= \textrm{i}\omega _{\alpha _{u,v}}(\mathcal {L}_{X_1^{\textrm{OD}\alpha _{u,v}}}(X_j^{\textrm{OD}\alpha _{u,v}})_0)= \textrm{i}\omega _{\alpha _{u,v}}(\mathcal {L}_{X_1}(X_j^{\textrm{OD}\alpha _{u,v}})_0)\\&= \textrm{i}\omega _0(\alpha _{u,v} \mathcal {L}_{X_1} (X_j^{\textrm{OD}\alpha _{u,v}})_0) ~. \end{aligned}$$

Inserting the definitions of $\mathcal {L}_{X_1}$ and $(X_j^{\textrm{OD}\alpha _{u,v}})_0$ we get

$$\begin{aligned}&\overline{\omega _{\alpha _{u,v}}}(\textrm{i}[ X_1^{\textrm{OD}\alpha _{u,v}},X_j^{\textrm{OD}\alpha _{u,v}}]) \\  &\quad = \textrm{i}\omega _0(\alpha _{u,v} \sum _{x\in \mathbb {Z}^d} \Big [x_1 n_x , \textrm{i}\alpha _{u,v}^{-1} \int _{\mathbb {R}}\textrm{d}s \,W_g(s)\, \textrm{e}^{\textrm{i}s\mathcal {L}_{H}} \alpha _{u,v} \mathcal {L}_{X_j} \alpha _{u,v}^{-1} h_0\Big ])\\&\quad = -\sum _{x\in \mathbb {Z}^d} \int _{\mathbb {R}}\textrm{d}s\, \omega _0( \Big [\alpha _{u,v} x_1 n_x , W_g(s)\, \textrm{e}^{\textrm{i}s\mathcal {L}_{H}} \alpha _{u,v} \mathcal {L}_{X_j} \alpha _{u,v}^{-1} h_0\Big ]) \\&\quad = -\sum _{x\in \mathbb {Z}^d} \int _{\mathbb {R}}\textrm{d}s\, \omega _0( \Big [\alpha _{u,v} x_1 n_x , W_g(s)\, \textrm{e}^{\textrm{i}s\mathcal {L}_{H}} \alpha _{u,v} \sum _{y \in \mathbb {Z}^d} [y_j n_y, \alpha _{u,v}^{-1} h_0]\,\Big ]) \\&\quad = -\sum _{x\in \mathbb {Z}^d} \int _{\mathbb {R}}\textrm{d}s\, \sum _{y \in \mathbb {Z}^d} \omega _0( \Big [\alpha _{u,v} x_1 n_x , W_g(s)\, \textrm{e}^{\textrm{i}s\mathcal {L}_{H}} [\alpha _{u,v} y_j n_y, h_0]\,\Big ]) \, , \end{aligned}$$

where we used that the sum and the integral converge in norm and that therefore the continuous linear maps $\alpha _{u,v}:\mathcal {A}\rightarrow \mathcal {A}$ and $\omega _0:\mathcal {A}\rightarrow \mathbb {C}$ can be exchanged with the limits involved in the definition of the sum and the integral. Next we want to differentiate the expression with respect to *v*. To prove that we can commute the derivative past the sum and integral, we show that the derivative is uniformly summable/integrable on bounded intervals. Meaning that for bounded intervals $I \subseteq \mathbb {R}$, it holds that

$$\begin{aligned} \sum _{x\in \mathbb {Z}^d} \int _{\mathbb {R}}\textrm{d}s \sum _{y \in \mathbb {Z}^d} \sup _{u \in I} \Vert {\partial _v \, \omega _0( \big [\alpha _{u,v} x_1 n_x , W_g(s)\, \textrm{e}^{\textrm{i}s\mathcal {L}_{H}} [\alpha _{u,v} y_j n_y, h_0]\,\big ])}\Vert < \infty ~. \end{aligned}$$

Using the chain rule and that $(\alpha _{u,v})_{(u,v) \in \mathbb {R}^2}$ is generated by $(\varPhi ^{v})_{v\in \mathbb {R}}$ we see that

$$\begin{aligned}&\partial _v \, \omega _0( \left[ \alpha _{u,v} x_1 n_x , W_g(s)\, \textrm{e}^{\textrm{i}s\mathcal {L}_{H}} [\alpha _{u,v} y_j n_y, h_0]\,\right] ) \\&\quad =\omega _0( \left[ \alpha _{u,v} \textrm{i}\mathcal {L}_{\varPhi ^v} x_1 n_x , W_g(s)\, \textrm{e}^{\textrm{i}s\mathcal {L}_{H}} [\alpha _{u,v} y_j n_y, h_0]\,\right] ) \\&\qquad + \omega _0( \left[ \alpha _{u,v} x_1 n_x , W_g(s)\, \textrm{e}^{\textrm{i}s\mathcal {L}_{H}} [\alpha _{u,v} \textrm{i}\mathcal {L}_{\varPhi ^v} y_j n_y, h_0]\,\right] ) \\&\quad {=}{:}S_1(v,x,s,y) + S_2(v,x,s,y) ~. \end{aligned}$$

We can treat the two summands separately. The fact that states have unit norm and Lemma A.1 (with $\nu =0$ and $m=d+2$) leads us to estimate

$$\begin{aligned} \sup _{v \in I} |{S_1(v,x,s,y)}| =&\sup _{v \in I} |{\omega _0( \left[ \alpha _{u,v} \mathcal {L}_{\varPhi ^v} x_1 n_x , W_g(s)\, \textrm{e}^{\textrm{i}s\mathcal {L}_{H}} [\alpha _{u,v} y_j n_y, h_0]\,\right] )}| \\ \le&\sup _{v \in I} \Vert { \left[ \alpha _{u,v} \mathcal {L}_{\varPhi ^v} x_1 n_x , W_g(s)\, \textrm{e}^{\textrm{i}s\mathcal {L}_{H}} [\alpha _{u,v} y_j n_y, h_0]\,\right] }\Vert \\ \le&\sup _{v \in I} 4^{d+5} \frac{\Vert { \alpha _{u,v} \mathcal {L}_{\varPhi ^v} x_1 n_0}\Vert _{d+2}}{(1+ \Vert {x}\Vert _{\infty })^{d+2}} |W_g(s)| \, \Vert { \textrm{e}^{\textrm{i}s\mathcal {L}_{H}} \left[ \alpha _{u,v} y_j n_y, h_0\right] }\Vert _{d+2} ~. \end{aligned}$$

Applying Lemma 2.6 to both $\alpha _{u,v}$ and $\textrm{e}^{\textrm{i}s\mathcal {L}_{H}}$ tells us that there is a constant *c* and an at most polynomially growing function *b*, such that

$$\begin{aligned} \sup _{v \in I} |{S_1(v,x,s,y)}| \le&\;\sup _{v \in I} c \, \exp (c \, |u-v|) \, \\  &\quad \frac{\Vert { \mathcal {L}_{\varPhi ^v} x_1 n_0}\Vert _{d+2}}{(1+ \Vert {x}\Vert _{\infty })^{d+2}} |W_g(s)| \, b(|{s}|) \Vert { [\alpha _{u,v} y_j n_y, h_0]}\Vert _{d+2} ~, \end{aligned}$$

from where we use Lemma A.1 (with $\nu =d+2$ and $m=d+2$) again to arrive at

$$\begin{aligned} \sup _{v \in I} |{S_1(v,x,s,y)}| \le&\;\sup _{v \in I} c \, \exp (c \, |u-v|) \, \frac{\Vert { \mathcal {L}_{\varPhi ^v} x_1 n_0}\Vert _{d+2}}{(1+ \Vert {x}\Vert _{\infty })^{d+2}} |W_g(s)| \, b(|{s}|)\, 4^{ 2d+7}\\  &\quad \frac{\Vert {\alpha _{u,v} y_j n_0}\Vert _{2d+4}}{(1+\Vert {y}\Vert _\infty )^{d+2}} \Vert {h_0}\Vert _{2d+4}\, . \end{aligned}$$

Another use of Lemma 2.6 gives us the existence of a constant $\tilde{c}$ such that

$$\begin{aligned} \sup _{v \in I} |{S_1(v,x,s,y)}|&\le \sup _{v \in I} \tilde{c} \, \exp (\tilde{c} \, |u-v|) \,\\  &\quad \frac{\Vert { \mathcal {L}_{\varPhi ^v} x_1 n_0}\Vert _{d+2}}{(1+ \Vert {x}\Vert _{\infty })^{d+2}} |W_g(s)| \, b(|{s}|)\frac{\Vert { y_j n_0}\Vert _{2d+4}}{(1+\Vert {y}\Vert _\infty )^{d+2}} \Vert {h_0}\Vert _{2d+4}\,. \end{aligned}$$

Finally, we exploit the assumption that $\sup _{v\in \mathbb {R}} \Vert {\varPhi ^v}\Vert _{3d+9} <\infty$ with the bound from Lemma 2.5 to get that $\Vert { \mathcal {L}_{\varPhi ^v} n_0}\Vert _{d+4}$ is bounded in *v*. Since the exponential in the expression is bounded on *I* we get the existence of a constant *C*, such that

$$\begin{aligned} \sup _{v \in I} |{S_1(v,x,s,y)}| \le C \frac{|x_1|}{(1+ \Vert {x}\Vert _{\infty })^{d+2}} |W_g(s)| \, b(|{s}|) \frac{\Vert { y_j n_0}\Vert _{2d+4}}{(1+\Vert {y}\Vert _\infty )^{d+2}} \Vert {h_0}\Vert _{2d+4} ~, \end{aligned}$$

which is summable/integrable in *x*, *s* and *y*. An analogous calculation shows that this also holds for $S_2(v,x,s,y)$. Thus, the map

$$\begin{aligned}\mathbb {R}\ni v \mapsto \textrm{i}\omega _0(\alpha _{u,v} \mathcal {L}_{X_1} (X_j^{\textrm{OD}\alpha _{u,v}})_0)\in \mathbb {R}\end{aligned}$$

is differentiable and

$$\begin{aligned} \partial _v \textrm{i}\omega _0(\alpha _{u,v} \mathcal {L}_{X_1} (X_j^{\textrm{OD}\alpha _{u,v}})_0) = - \sum _{x\in \mathbb {Z}^d} \int _{\mathbb {R}}\textrm{d}s \sum _{y \in \mathbb {Z}^d} \left( S_1(v,x,s,y) + S_2(v,x,s,y)\right) ~. \end{aligned}$$

We find that by definition of the off-diagonal map (Definition 4.3) and Proposition 2.11

$$\begin{aligned}&- \sum _{x\in \mathbb {Z}^d} \int _{\mathbb {R}}\textrm{d}s \sum _{y \in \mathbb {Z}^d} S_1(v,x,s,y) \\  &\quad = -\omega _0( \alpha _{u,v} \sum _{x\in \mathbb {Z}^d} \Big [ \textrm{i}\mathcal {L}_{\varPhi ^v} x_1 n_x ,\alpha _{u,v}^{-1} \int _{\mathbb {R}}\textrm{d}s W_g(s)\, \textrm{e}^{\textrm{i}s\mathcal {L}_{H}} \alpha _{u,v} \sum _{y \in \mathbb {Z}^d} [ y_j n_y, \alpha _{u,v}^{-1} h_0]\Big ])\\&\quad =\textrm{i}\omega _0( \alpha _{u,v} \sum _{x\in \mathbb {Z}^d}\left[ \textrm{i}\mathcal {L}_{\varPhi ^v} x_1 n_x ,(X_j^{\textrm{OD}\alpha _{u,v}})_0\right] )~, \end{aligned}$$

which by Proposition 2.13 and Lemma 4.4 is equal to

$$\begin{aligned} \textrm{i}\, \omega _{\alpha _{u,v}}( \mathcal {L}_{\textrm{i}[\varPhi ^v,X_1]} \, (X_j^{\textrm{OD}\alpha _{u,v}})_0)&= -\textrm{i}\, \omega _{\alpha _{u,v}}( \mathcal {L}_{X_j} \, (\textrm{i}\, [\, \varPhi ^v,\, X_1\, ] )_0) \\&= -\omega _{\alpha _{u,v}}( \mathcal {L}_{X_j} \, \mathcal {L}_{X_{ 1}}\,\varPhi ^v_0) \end{aligned}$$

We also have

$$\begin{aligned}&- \sum _{x\in \mathbb {Z}^d} \int _{\mathbb {R}} \textrm{d}s \sum _{y \in \mathbb {Z}^d} S_2 (v,x,s,y)\\  &\quad = - \omega _0( \alpha _{u,v} \sum _{x\in \mathbb {Z}^d} \Big [ x_1 n_x ,\alpha _{u,v}^{-1} \int _{\mathbb {R}}\textrm{d}s W_g(s)\, \textrm{e}^{\textrm{i}s\mathcal {L}_{H}} \alpha _{u,v} \sum _{y \in \mathbb {Z}^d} [ \textrm{i}\mathcal {L}_{\varPhi ^v} y_j n_y, \alpha _{u,v} ^{-1} h_0]\,\Big ])~. \end{aligned}$$

Using Propositions 2.13 and 2.12 we can see that this is equal to

$$\begin{aligned}&\textrm{i}\, \omega _0( \alpha _{u,v} \sum _{x\in \mathbb {Z}^d} \Big [ x_1 n_x ,\alpha _{u,v}^{-1} \int _{\mathbb {R}}\textrm{d}s W_g(s)\, \textrm{e}^{\textrm{i}s\mathcal {L}_{H}} \alpha _{u,v} \textrm{i}\mathcal {L}_{\textrm{i}[\varPhi ^v,X_j]}\,\alpha _{u,v} ^{-1} h_0\,\Big ])\\&\quad = \textrm{i}\, \omega _{\alpha _{u,v}}(\mathcal {L}_{X_1} \, (\textrm{i}\,[\, \varPhi ^v,\,X_j\,]^{\textrm{OD}\alpha _{u,v}})_0)\, . \end{aligned}$$

Further using Proposition 2.13 and Lemma 4.4 lets us rewrite the expression as follows:

$$\begin{aligned} \textrm{i}\, \omega _{\alpha _{u,v}}(\mathcal {L}_{X_1} \, (\textrm{i}\,[\, \varPhi ^u,\,X_j\,]^{\textrm{OD}\alpha _{u,v}})_0)&= -\textrm{i}\, \omega _{\alpha _{u,v}}(\mathcal {L}_{\textrm{i}[ \varPhi ^u,X_j]^{\textrm{OD}\alpha _{u,v}}} \, (X_1^{\textrm{OD}\alpha _{u,v}})_0)\\&= -\textrm{i}\, \omega _{\alpha _{u,v}}(\mathcal {L}_{\textrm{i}[ \varPhi ^u,X_j]} \, (X_1^{\textrm{OD}\alpha _{u,v}})_0)\\&= \textrm{i}\, \omega _{\alpha _{u,v}}(\mathcal {L}_{X_1} \, (\textrm{i}\,[\, \varPhi ^u,\,X_j\,])_0)\\&= \omega _{\alpha _{u,v}}(\mathcal {L}_{X_1} \, \mathcal {L}_{X_j}\, \varPhi ^u_0)\, . \end{aligned}$$

It therefore holds that

$$\begin{aligned} \partial _v \textrm{i}\omega _0(\alpha _{u,v} \mathcal {L}_{X_1} (X_j^{\textrm{OD}\alpha _{u,v}})_0) = -\omega _{\alpha _{u,v}}( \mathcal {L}_{X_j} \, \mathcal {L}_{X_{ 1}}\,\varPhi ^v_0) + \omega _{\alpha _{u,v}}(\mathcal {L}_{X_1} \, \mathcal {L}_{X_j}\, \varPhi ^v_0) = 0\,. \end{aligned}$$

## Appendix E: Proof of Corollary 5.4

For $m \in \mathbb {N}_0$ we denote the CAR-algebra associated to the Hilbert space $\ell ^2(\mathbb {Z}^d,\mathbb {C}^m)$ by $\textrm{CAR}(\ell ^2(\mathbb {Z}^d,\mathbb {C}^m))$. For $m,k \in \mathbb {N}_0$ we can identify $\textrm{CAR}(\ell ^2(\mathbb {Z}^d,\mathbb {C}^{m+k}))$ with the $\mathbb {Z}_2$-graded tensor product

$$\begin{aligned} \textrm{CAR}(\ell ^2(\mathbb {Z}^d,\mathbb {C}^m)) \otimes _{\mathbb {Z}_2} \textrm{CAR}(\ell ^2(\mathbb {Z}^d,\mathbb {C}^k)). \end{aligned}$$

### Remark E.1

Since the CAR-algebra of a separable Hilbert space is nuclear, there is a unique tensor product C$^*$-algebra $\textrm{CAR}(\ell ^2(\mathbb {Z}^d,\mathbb {C}^m)) \otimes \textrm{CAR}(\ell ^2(\mathbb {Z}^d,\mathbb {C}^k))$. The $\mathbb {Z}_2$-graded tensor product is obtained from the tensor product by modifying multiplication and star operation as explained in the following. An element $A \in \textrm{CAR}(\ell ^2(\mathbb {Z}^d, \mathbb {C}^m))$ is said to be even (resp. odd) if it satisfies $g_{ \pi }(A) = A$ (resp. $g_{ \pi }(A) = -A$). Here, the automorphism $g_{\pi }$ of $\textrm{CAR}(\ell ^2(\mathbb {Z}^d, \mathbb {C}^m))$ is given by $g_{ \pi }(a_{x, i}^*) =\textrm{e}^{\textrm{i}\pi }a_{x, i}^*= -a_{x, i}^*$. For an odd or even element *A* of one of the CAR algebras we define

$$\begin{aligned} |{A}| = {\left\{ \begin{array}{ll} 0 &  \text {if}\, A\, \text {is even}\\ 1 &  \text {if}\, A\, \text {is odd.} \end{array}\right. } \end{aligned}$$

Now let $A_1,B_1 \in \textrm{CAR}(\ell ^2(\mathbb {Z}^d,\mathbb {C}^m))$ and $A_2,B_2 \in \textrm{CAR}(\ell ^2(\mathbb {Z}^d,\mathbb {C}^k))$ each be odd or even. We define the modified star operation and multiplication by

$$\begin{aligned} (A_1 \otimes A_2)^*&= (-1)^{|{A_1}| \, |{A_2}|} \,A_1^* \otimes A_2^*, \\ (A_1\otimes A_2)(B_1 \otimes B_2)&= (-1)^{|{A_2}| \, |{B_1}|} \,A_1\, B_1 \otimes A_2 \, B_2 \end{aligned}$$

and linear extension. The resulting C$^*$-algebra $\textrm{CAR}(\ell ^2(\mathbb {Z}^d,\mathbb {C}^m)) \otimes _{\mathbb {Z}_2} \textrm{CAR}(\ell ^2(\mathbb {Z}^d,\mathbb {C}^k))$ is isomorphic to $\textrm{CAR}(\ell ^2(\mathbb {Z}^d,\mathbb {C}^{m+k}))$.

### Definition E.2

Let $\omega$ on $\mathcal {A}= \textrm{CAR}(\ell ^2(\mathbb {Z}^d,\mathbb {C}^n))$ be the unique gapped ground state of a Hamiltonian *H* in $B_{\exp }$. We call $\omega$ invertible if there are an $m\in \mathbb {N}_0$ and a state $\tilde{\omega }$ on $\textrm{CAR}(\ell ^2(\mathbb {Z}^d,\mathbb {C}^m))$ that is the unique gapped ground state of a Hamiltonian $\tilde{H}$ in $B_{\exp }$, such that there exists a family of interactions $(\varPhi ^u)_{u\in \mathbb {R}}$ on $\textrm{CAR}(\ell ^2(\mathbb {Z}^d,\mathbb {C}^{n+m}))$ in $B_{\exp }$ with unique gapped ground states $(\omega _u)_{u\in \mathbb {R}}$ that satisfies:

(i) $$\varPhi ^0 = H \otimes \mathbb {1} + \mathbb {1} \otimes \tilde{H}$$, i.e. $$\varPhi ^0(M) = H(M) \otimes \mathbb {1} + \mathbb {1} \otimes \tilde{H}(M)$$ for all $$M\in P_0(\mathbb {Z}^d)$$.
(ii) $\forall M \in P_0(\mathbb {Z}^d)$ the map $\mathbb {R}\rightarrow \textrm{CAR}(\ell ^2(\mathbb {Z}^d,\mathbb {C}^{n+m})),\, u\mapsto \varPhi ^u(M)$ is differentiable.
(iii) $\varPhi ^1$ is supported only on singletons.

### Proposition E.3

Let $({H,W,T,({S_{\varepsilon }})_{\varepsilon \in \mathbb {R}}})$ be a gapped periodic system in dimension $d=2$. For $j\in \{1,2\}$ we denote the right/upper half plane respectively by

$$\begin{aligned} \varSigma _j {:}{=}\{ (x_1,x_2)\in \mathbb {Z}^2 \, | \, x_j \ge 0\}\,. \end{aligned}$$

We further define

$$\begin{aligned} K_{x,x'} = \int _{\mathbb {R}} \textrm{d}s \, W_g(s)\, \textrm{e}^{\textrm{i}s\mathcal {L}_H} \left( \textrm{i}[T_x h_0,n_{x'}] - \textrm{i}[T_{x'} h_0, n_x]\right) \end{aligned}$$

for $$x,x' \in \mathbb {Z}^2$$ and

$$\begin{aligned} {[}K_{\varSigma _1,\varSigma _1^{\textsf{C}}}, K_{\varSigma _2,\varSigma _2^{\textsf{C}}}] = \sum _{x\in \varSigma _1} \sum _{x'\in \varSigma _1^{\textsf{C}}}\sum _{y\in \varSigma _2} \sum _{y'\in \varSigma _2^{\textsf{C}}} [K_{x,x'},K_{y,y'}]\,. \end{aligned}$$

It holds that

19 $$\begin{aligned} \overline{\omega _0}(\textrm{i}[X_1^{\textrm{OD}},X_2^{\textrm{OD}}]) = \omega _0(\textrm{i}[K_{\varSigma _1,\varSigma _1^{\textsf{C}}}, K_{\varSigma _2,\varSigma _2^{\textsf{C}}}])\, . \end{aligned}$$

In [31] it is shown that, if $\omega _0$ is invertible in the sense of Definition E.2, the right-hand side of (19) lies in $\frac{1}{2\pi } \mathbb {Z}$ (the relevant statements can be found in [31, Theorem 1] and [31, Equation 55]). The previous result then allows to conclude the proof of Corollary 5.4 on quantization of the Hall conductivity in phases connected to an invertible one.

### Proof of Proposition E.3

We observe that

$$\begin{aligned}&\sum _{y\in \varSigma _2}\sum _{y'\in \varSigma _2^{\textsf{C}}} [K_{x,x'},K_{y,y'}]\\&\quad =\sum _{y\in \varSigma _2} \sum _{y'\in \varSigma _2^{\textsf{C}}} \Big [K_{x,x'},\int _{\mathbb {R}} \textrm{d}s \, W_g(s)\, \textrm{e}^{\textrm{i}s\mathcal {L}_H} \textrm{i}[T_y h_0,n_{y'}]\Big ] \\&\qquad - \sum _{y\in \varSigma _2} \sum _{y'\in \varSigma _2^{\textsf{C}}} \Big [K_{x,x'},\int _{\mathbb {R}} \textrm{d}s \, W_g(s)\, \textrm{e}^{\textrm{i}s\mathcal {L}_H} \textrm{i}[T_{y'} h_0, n_{y}]\Big ]\, . \end{aligned}$$

Changing the order of summation/integration by using Lemmas A.1 and 2.6 to show absolute convergence results in

$$\begin{aligned}&\sum _{y\in \varSigma _2} \sum _{y'\in \varSigma _2^{\textsf{C}}} [K_{x,x'},K_{y,y'}]\\&\quad =-\textrm{i}\sum _{y\in \varSigma _2} \Big [K_{x,x'},\int _{\mathbb {R}} \textrm{d}s \, W_g(s)\, \textrm{e}^{\textrm{i}s\mathcal {L}_H} \mathcal {L}_{N_{\varSigma _2^{\textsf{C}}}} T_y h_0\Big ]\\&\qquad + \textrm{i}\sum _{y'\in \varSigma _2^{\textsf{C}}} \Big [K_{x,x'},\int _{\mathbb {R}} \textrm{d}s \, W_g(s)\, \textrm{e}^{\textrm{i}s\mathcal {L}_H} \mathcal {L}_{N_{\varSigma _2}} T_{y'} h_0 \Big ]\,, \end{aligned}$$

where $N_{\varSigma }$ is the number operator restricted to $\varSigma$ for any $\varSigma \subseteq \mathbb {Z}^2$. Due to gauge-invariance we have

$$\begin{aligned} \mathcal {L}_{N_{\varSigma _2^{\textsf{C}}}} T_y h_0 = \mathcal {L}_N T_y h_0 - \mathcal {L}_{N_{\varSigma _2}} T_y h_0 = - \mathcal {L}_{N_{\varSigma _2}} T_y h_0\, , \end{aligned}$$

which yields

$$\begin{aligned} \begin{aligned}&\sum _{y\in \varSigma _2} \sum _{y'\in \varSigma _2^{\textsf {C}}} [K_{x,x'},K_{y,y'}]\\  &\quad = \text {i}\sum _{y\in \varSigma _2} \Big [K_{x,x'},\int _{\mathbb {R}} \text {d}s \, W_g(s)\, \text {e}^{\text {i}s\mathcal {L}_H} \mathcal {L}_{N_{\varSigma _2}} T_y h_0\Big ] \\  &\qquad + \text {i}\sum _{y'\in \varSigma _2^{\textsf {C}}} \Big [K_{x,x'},\int _{\mathbb {R}} \text {d}s \, W_g(s)\, \text {e}^{\text {i}s\mathcal {L}_H} \mathcal {L}_{N_{\varSigma _2}} T_{y'} h_0\Big ]\\  &\quad = \text {i}\sum _{y \in \mathbb {Z}^2} \Big [K_{x,x'},\int _{\mathbb {R}} \text {d}s \, W_g(s)\, \text {e}^{\text {i}s\mathcal {L}_H} \mathcal {L}_{N_{\varSigma _2}} T_y h_0\Big ]\,. \end{aligned} \end{aligned}$$

Next we reintroduce the remaining summations and consider the expression

$$\begin{aligned} \textrm{i}\sum _{x\in \varSigma _1} \sum _{x'\in \varSigma _1} \sum _{y \in \mathbb {Z}^2} \Big [K_{x,x'},\int _{\mathbb {R}} \textrm{d}s \, W_g(s)\, \textrm{e}^{\textrm{i}s\mathcal {L}_H} \mathcal {L}_{N_{\varSigma _2}} T_y h_0\Big ]\, , \end{aligned}$$

where one can again use Lemmas 2.6 and A.1 to show absolute convergence and repeat the same argument from above to arrive at

$$\begin{aligned}&\sum _{x\in \varSigma _1} \sum _{x'\in \varSigma _1} \sum _{y\in \varSigma _2} \sum _{y'\in \varSigma _2^{\textsf{C}}} [K_{x,x'},K_{y,y'}] \\  &\quad =-\sum _{x \in \mathbb {Z}^2} \sum _{y \in \mathbb {Z}^2} \Big [\int _{\mathbb {R}} \textrm{d}s \, W_g(s)\, \textrm{e}^{\textrm{i}s\mathcal {L}_H} \mathcal {L}_{N_{\varSigma _1}} T_x h_0,\int _{\mathbb {R}} \textrm{d}s \, W_g(s)\, \textrm{e}^{\textrm{i}s\mathcal {L}_H} \mathcal {L}_{N_{\varSigma _2}} T_y h_0\Big ]\,. \end{aligned}$$

We evaluate this expression under the periodic state $\omega _0$:

$$\begin{aligned} \begin{aligned} S\;&{:}{=}\sum _{x\in \varSigma _1} \sum _{x'\in \varSigma _1} \sum _{y\in \varSigma _2} \sum _{y'\in \varSigma _2^{\textsf {C}}} \text {i}\omega _0([K_{x,x'},K_{y,y'}]) \\  &=\sum _{x \in \mathbb {Z}^2} \sum _{y \in \mathbb {Z}^2} -\text {i}\omega _0(\Big [\int _{\mathbb {R}} \text {d}s \, W_g(s)\, \text {e}^{\text {i}s\mathcal {L}_H} \mathcal {L}_{N_{\varSigma _1}} T_x h_0,\\  &\quad \int _{\mathbb {R}} \text {d}s \, W_g(s)\, \text {e}^{\text {i}s\mathcal {L}_H} \mathcal {L}_{N_{\varSigma _2}} T_y h_0\Big ])\\  &=\sum _{x \in \mathbb {Z}^2} \sum _{y \in \mathbb {Z}^2} -\text {i}\omega _0(\Big [\int _{\mathbb {R}} \text {d}s \, W_g(s)\, \text {e}^{\text {i}s\mathcal {L}_H} T_{(x_1,0)}^{-1}\mathcal {L}_{N_{\varSigma _1}} T_x h_0,\\  &\quad \int _{\mathbb {R}} \text {d}s \, W_g(s)\, \text {e}^{\text {i}s\mathcal {L}_H} \mathcal {L}_{N_{\varSigma _2}} T_{(x_1,0)}^{-1} T_y h_0\Big ]) \, . \end{aligned} \end{aligned}$$

Since *H* is a *T*-periodic $B_\infty$-interaction it holds that $T_{\gamma +\mu } h_0 = T_\gamma T_\mu h_0$ for all $\gamma ,\mu \in \mathbb {Z}^2$ (as shown in Proposition 2.11), giving us

$$\begin{aligned} S&= \sum _{x \in \mathbb {Z}^2} \sum _{y \in \mathbb {Z}^2} -\textrm{i}\omega _0(\Big [\int _{\mathbb {R}} \textrm{d}s \, W_g(s)\, \textrm{e}^{\textrm{i}s\mathcal {L}_H} T_{(x_1,0)}^{-1}\mathcal {L}_{N_{\varSigma _1}} T_{(x_1,0)} T_{(0,x_2)} h_0,\\  &\quad \int _{\mathbb {R}} \textrm{d}s \, W_g(s)\, \textrm{e}^{\textrm{i}s\mathcal {L}_H} \mathcal {L}_{N_{\varSigma _2}} T_{(y_1-x_1,y_2)} h_0\Big ])\, . \end{aligned}$$

Now an index shift lets us eliminate $x_1$ in the second argument of the commutator, which allows us to sum over $x_1$ in the first argument by using Lemma E.4 below. This results in

$$\begin{aligned} S&= \sum _{x \in \mathbb {Z}^2} \sum _{y \in \mathbb {Z}^2} -\textrm{i}\omega _0(\Big [\int _{\mathbb {R}} \textrm{d}s \, W_g(s)\, \textrm{e}^{\textrm{i}s\mathcal {L}_H} T_{(x_1,0)}^{-1}\mathcal {L}_{N_{\varSigma _1}} T_{(x_1,0)} T_{(0,x_2)} h_0,\\  &\quad \int _{\mathbb {R}} \textrm{d}s \, W_g(s)\, \textrm{e}^{\textrm{i}s\mathcal {L}_H} \mathcal {L}_{N_{\varSigma _2}} T_{(y_1,y_2)} h_0\Big ])\\&=\sum _{x_2 \in \mathbb {Z}} \sum _{y \in \mathbb {Z}^2} -\textrm{i}\omega _0(\Big [\int _{\mathbb {R}} \textrm{d}s \, W_g(s)\, \textrm{e}^{\textrm{i}s\mathcal {L}_H} \mathcal {L}_{X_1} T_{(0,x_2)} h_0,\\  &\quad \int _{\mathbb {R}} \textrm{d}s \, W_g(s)\, \textrm{e}^{\textrm{i}s\mathcal {L}_H} \mathcal {L}_{N_{\varSigma _2}} T_{(y_1,y_2)} h_0\Big ])\\&=\sum _{x_2 \in \mathbb {Z}} \sum _{y \in \mathbb {Z}^2} -\textrm{i}\omega _0(\Big [\int _{\mathbb {R}} \textrm{d}s \, W_g(s)\, \textrm{e}^{\textrm{i}s\mathcal {L}_H} \mathcal {L}_{X_1} T_{(-y_1,x_2-y_2)} h_0,\\  &\quad \int _{\mathbb {R}} \textrm{d}s \, W_g(s)\, \textrm{e}^{\textrm{i}s\mathcal {L}_H} T_{(0,y_2)}^{-1}\mathcal {L}_{N_{\varSigma _2}} T_{(0,y_2)} h_0\Big ])\, , \end{aligned}$$

where we used gauge-invariance of $h_0$ and periodicity to move the Translation operators in the last step. Another index shift lets us eliminate $y_2$ in the first argument of the commutator and apply Lemma E.4 to get

$$\begin{aligned} S&= \sum _{x_2 \in \mathbb {Z}} \sum _{y \in \mathbb {Z}^2} -\textrm{i}\omega _0(\Big [\int _{\mathbb {R}} \textrm{d}s \, W_g(s)\, \textrm{e}^{\textrm{i}s\mathcal {L}_H} \mathcal {L}_{X_1} T_{(-y_1,x_2)} h_0,\\  &\quad \int _{\mathbb {R}} \textrm{d}s \, W_g(s)\, \textrm{e}^{\textrm{i}s\mathcal {L}_H} T_{(0,y_2)}^{-1}\mathcal {L}_{N_{\varSigma _2}} T_{(0,y_2)} h_0\Big ])\\&=\sum _{x_2 \in \mathbb {Z}} \sum _{y_1 \in \mathbb {Z}} -\textrm{i}\omega _0(\Big [\int _{\mathbb {R}} \textrm{d}s \, W_g(s)\, \textrm{e}^{\textrm{i}s\mathcal {L}_H} \mathcal {L}_{X_1} T_{(-y_1,x_2)} h_0,\\  &\quad \int _{\mathbb {R}} \textrm{d}s \, W_g(s)\, \textrm{e}^{\textrm{i}s\mathcal {L}_H} \mathcal {L}_{X_2} h_0\Big ])\, . \end{aligned}$$

By gauge-invariance of $h_0$ and periodicity of *H* we can move the translation in front of the integral

$$\begin{aligned} S&= \sum _{x_2 \in \mathbb {Z}} \sum _{y_1 \in \mathbb {Z}} -\textrm{i}\omega _0\left( \Big [T_{(-y_1,x_2)} \int _{\mathbb {R}} \textrm{d}s \, W_g(s)\, \textrm{e}^{\textrm{i}s\mathcal {L}_H} \mathcal {L}_{X_1} h_0,\int _{\mathbb {R}} \textrm{d}s \, W_g(s)\, \textrm{e}^{\textrm{i}s\mathcal {L}_H} \mathcal {L}_{X_2} h_0\Big ]\right) \\&= \sum _{\gamma \in \mathbb {Z}^2} -\textrm{i}\omega _0\left( \Big [T_\gamma \int _{\mathbb {R}} \textrm{d}s \, W_g(s)\, \textrm{e}^{\textrm{i}s\mathcal {L}_H} \mathcal {L}_{X_1} h_0,\int _{\mathbb {R}} \textrm{d}s \, W_g(s)\, \textrm{e}^{\textrm{i}s\mathcal {L}_H} \mathcal {L}_{X_2} h_0\Big ]\right) \,. \end{aligned}$$

With the definition of the off-diagonal map (Lemma 4.2), along with Propositions 2.12 and 2.13, we now find:

$$\begin{aligned}&S = \sum _{\gamma \in \mathbb {Z}^2} \textrm{i}\omega _0([T_\gamma (X_1^{\textrm{OD}})_0, (X_2^{\textrm{OD}})_0])\;= \;\overline{\omega _0}(\textrm{i}[ X_1^{\textrm{OD}}, X_2^{\textrm{OD}}])\,. \end{aligned}$$

$$\square $$

### Lemma E.4

Let $d=2$ and let *T* be a translation. For $j\in \{1,2\}$ let $N_{\varSigma _j}$ be the number operator restricted to the half plane $\varSigma _j$ and let $A\in D_\infty$. It holds that

$$\begin{aligned} \sum _{y_1 \in \mathbb {Z}} T_{(y_1,0)}^{-1} \mathcal {L}_{N_{\varSigma _1}}T_{(y_1,0)} A = \mathcal {L}_{X_1} A \, \qquad \text{ and }\qquad \sum _{y_2 \in \mathbb {Z}} T_{(0,y_2)}^{-1} \mathcal {L}_{N_{\varSigma _2}}T_{(0,y_2)} A = \mathcal {L}_{X_2} A \,. \end{aligned}$$

### Proof

We start by considering a local gauge-invariant operator $A\in \mathcal {A}_0^N$. Let $k\in \mathbb {N}_0$ be such that $A \in \mathcal {A}_{\Lambda _k}$. We consider

$$\begin{aligned} \mathcal {L}_{X_1} A&= \sum _{x_2 = -k}^k \sum _{x_1 = -k}^k [x_1n_{(x_1,x_2)},A] \end{aligned}$$

and use the gauge-invariance of *A* to get

$$\begin{aligned} \mathcal {L}_{X_1} A&= \sum _{x_2 = -k}^k \sum _{x_1 = -k}^k [x_1n_{(x_1,x_2)},A] + (k+1) \mathcal {L}_N A \\  &= \sum _{x_2 = -k}^k \sum _{x_1 = -k}^k [(x_1+k+1)n_{(x_1,x_2)},A] \\&= \sum _{x_2 = -k}^k \sum _{x_1 = -k}^k \sum _{z=0}^{x_1+k}[n_{(x_1,x_2)},A] \,. \end{aligned}$$

Next we employ the indicator function $\chi$ to change the order of summation

$$\begin{aligned} \mathcal {L}_{X_1} A&= \sum _{x_2 = -k}^k \sum _{x_1 = -k}^k \sum _{z=-k}^{x_1} [n_{(x_1,x_2)},A] \;= \sum _{x_2 = -k}^k \sum _{x_1 = -k}^k \sum _{z=-k}^{k} \chi _{z \le x_1} [n_{(x_1,x_2)},A]\\&= \sum _{x_2 = -k}^k \sum _{z=-k}^{k} \sum _{x_1 = z}^{k} [n_{(x_1,x_2)},A] \,. \end{aligned}$$

Finally, using an index shift and the shape of the support of *A* we arrive at

$$\begin{aligned}&\mathcal {L}_{X_1} A = \sum _{x_2 = -k}^k \sum _{z=-k}^{k} \sum _{x_1 = 0}^{k} [n_{(x_1+z,x_2)},A] \\  &= \sum _{z=-k}^{k} T_{(-z,0)}^{-1}\mathcal {L}_{N_{\varSigma _1}} T_{(-z,0)} A \;= \;\sum _{z\in \mathbb {Z}} T_{(z,0)}^{-1}\mathcal {L}_{N_{\varSigma _1}} T_{(z,0)} A \, . \end{aligned}$$

Now let $A\in D_\infty$. The sum

$$\begin{aligned} S(A) {:}{=}\sum _{y_1 \in \mathbb {Z}} T_{(y_1,0)}^{-1} \mathcal {L}_{N_{\varSigma _1}}T_{(y_1,0)} A \end{aligned}$$

converges absolutely. This can be seen by splitting it in two parts and using gauge-invariance to get

$$\begin{aligned} S(A) = - \sum _{y_1 = 0 }^\infty \sum _{x\in \varSigma _1^{\textsf{C}}} [n_{x-(y_1,0)},A] + \sum _{y_1 = 1 }^\infty \sum _{x\in \varSigma _1} [n_{x+(y_1,0)},A]\, . \end{aligned}$$

The estimates

$$\begin{aligned}&\Vert {[n_{x\pm (y_1,0)},A]}\Vert \le 4^{8} \frac{\Vert {n_0}\Vert _5 \, \Vert {A}\Vert _5}{(1 + \Vert {x\pm (y_1,0)}\Vert _\infty )^5} \end{aligned}$$

from Lemma A.1 give us absolute convergence and further tell us that *S*(*A*) goes to 0 if $\Vert {A}\Vert _5$ goes to 0. For $k\in \mathbb {N}_0$ we have

$$\begin{aligned} \sum _{y_1 \in \mathbb {Z}} T_{(y_1,0)}^{-1} \mathcal {L}_{N_{\varSigma _1}}T_{(y_1,0)} A&= \sum _{y_1 \in \mathbb {Z}} T_{(y_1,0)}^{-1} \mathcal {L}_{N_{\varSigma _1}}T_{(y_1,0)} \mathbb {E}_{\Lambda _k}A \\  &\quad + \sum _{y_1 \in \mathbb {Z}} T_{(y_1,0)}^{-1} \mathcal {L}_{N_{\varSigma _1}}T_{(y_1,0)} (A -\mathbb {E}_{\Lambda _k}A)\\&= \mathcal {L}_{X_1} A -\mathcal {L}_{X_1}(A -\mathbb {E}_{\Lambda _k}A) + S(A -\mathbb {E}_{\Lambda _k}A)\,. \end{aligned}$$

Since $\Vert {A -\mathbb {E}_{\Lambda _k}A}\Vert _\nu$ goes to 0 as $k\rightarrow \infty$ for all $\nu$ (Lemma 2.3), our previous considerations and Lemma 2.5 imply that the last two terms vanish as $k \rightarrow \infty$. This shows the claim for $N_{\varSigma _1}$. The claim for $N_{\varSigma _2}$ can be proved analogously. $\square$

## Appendix F: Proof of Proposition 3.3

The construction of $S_\varepsilon$ was first done in [46] for finite systems with bounds uniform in the system size and then in [28, 29] for infinitely extended systems in the thermodynamic limit. However, in these works the focus was on the adiabatic theorem and also on the thermodynamic limit itself. Therefore the proofs are not only very technical, but in [28, 29] it was also assumed that the ground state $\omega _0$ is unique. Since uniqueness is very difficult to establish in concrete problems and not needed for our results, we provide here a construction of the NEASS in infinite volume without assuming that $\omega _0$ is the unique ground state.

The interaction $S_\varepsilon$ is constructed as a suitable resummation of a formal power series $\sum _{\mu =1}^\infty \varepsilon ^\mu K_\mu$ with coefficients $K_\mu \in B_{\infty ,T}$ for $\mu \in \mathbb {N}$. Within this proof we denote by $B_{\infty ,T}$ the space of *T*-periodic elements of $B_\infty$. Roughly speaking, the coefficients $K_\mu$ are determined by the requirement that

$$\begin{aligned} \omega _\varepsilon (\mathcal {L}_{H_{\varepsilon }}A) \;= \;\omega _0(\beta _\varepsilon \mathcal {L}_{ H_\varepsilon } A) \;= \; \omega _0(\mathcal {L}_{\beta _\varepsilon H_\varepsilon } \beta _\varepsilon A) \;= \; \omega _0(\mathcal {L}_{(\beta _\varepsilon H_\varepsilon )^{\textrm{OD}}} \beta _\varepsilon A) \;{\mathop {=}\limits ^{!}} \;\mathcal {O}(\varepsilon ^\infty ), \end{aligned}$$

i.e. by constructing $\beta _\varepsilon =\textrm{e}^{\textrm{i}\mathcal {L}_{S_{\varepsilon }}}$ such that it block-diagonalizes $H_\varepsilon$ with respect to $\omega _0$ by achieving $(\beta _\varepsilon H_\varepsilon )^{\textrm{OD}}=\mathcal {O}(\varepsilon ^\infty )$.

More precisely, assume that $K_\mu \in B_{\infty ,T}$ and let $\beta _\varepsilon ^{(m)}(t):= \textrm{e}^{\textrm{i}t \mathcal {L}_{S_{\varepsilon }^{(m)}}}$ with $S_\varepsilon ^{(m)}:= \sum _{\mu =1}^m \varepsilon ^\mu K_\mu$ with $m\in \mathbb {N}$. Then

$$\begin{aligned} \omega _0(\beta _\varepsilon ^{(m)}(1)\mathcal {L}_{H_{\varepsilon }}A) = \omega _0(\beta _\varepsilon ^{(m)}(t)\mathcal {L}_{H_{\varepsilon }} \beta _\varepsilon ^{(m)}(-t)\beta _\varepsilon ^{(m)}(1)A)|_{t=1} . \end{aligned}$$

For any $B\in D_\infty$ Taylor expansion at $t=0$, with the mean-value form of the remainder, yields

$$\begin{aligned}  &   \beta _\varepsilon ^{(m)}(t)\mathcal {L}_{H_{\varepsilon }} \beta _\varepsilon ^{(m)}(-t) B|_{t=1}\\  &   \quad = \sum _{k=0}^m \frac{1}{k!} \frac{\textrm{d}^k}{\textrm{d}t^k}\left( \beta _\varepsilon ^{(m)}(t)\mathcal {L}_{H_{\varepsilon }} \beta _\varepsilon ^{(m)}(-t) B\right) |_{t=0}\, \\    &   \qquad +\,\frac{1}{(m+1)!} \frac{\textrm{d}^{m+1}}{\textrm{d}t^{m+1}}\left( \beta _\varepsilon ^{(m)}(t)\mathcal {L}_{H_{\varepsilon }} \beta _\varepsilon ^{(m)}(-t) B\right) |_{t=\tau }\\  &   \quad = \sum _{k=0}^m \frac{1}{k!} \mathcal {L}_{\textrm{ad}({\textrm{i}S_\varepsilon ^{(m)}})^k H_\varepsilon } B\,+\, \frac{1}{(m+1)!} \beta _\varepsilon ^{(m)}(\tau ) \mathcal {L}_{\textrm{ad}({\textrm{i}S_\varepsilon ^{(m)}})^{m+1} H_\varepsilon } \beta _\varepsilon ^{(m)}(-\tau ) B \end{aligned}$$

for some $\tau \in [0,1]$, where $\textrm{ad}({\textrm{i}S_\varepsilon ^{(m)}})^k H_\varepsilon$ denotes the *k* times iterated commutator of $\textrm{i}S_\varepsilon ^{(m)}$ with $H_\varepsilon$. Now

$$\begin{aligned} \sum _{k=0}^m\frac{1}{k!}\,\textrm{ad}({\textrm{i}S_\varepsilon ^{(m)}})^k H_\varepsilon= &   \sum _{k=0}^m\frac{1}{k!}\, \textrm{ad}({\textrm{i}S_\varepsilon ^{(m)}})^k (H + \varepsilon (X_1+V)) \\=: &   \sum _{\mu =0}^m \varepsilon ^\mu (H_{\mu } + V_{\mu }) + \varepsilon ^{m+1}R^{(m)}_\varepsilon \end{aligned}$$

with

$$\begin{aligned} H_{\mu } = \textrm{i}[K_\mu , H] + L_\mu \end{aligned}$$

and $L_\mu$ being a sum of iterated commutators of $K_j$’s with *H* for $j<\mu$ and $V_{\mu }$ a sum of iterated commutators of $K_j$’s with $X_1 + V$ for $j<\mu$. In $\varepsilon ^{m+1}R^{(m)}_\varepsilon$ we collect all terms that contain at least $m+1$ factors of $\varepsilon$, so that $R^{(m)}_\varepsilon$ is a finite sum of iterated commutators of elements of $B_{\infty ,T}$ and thus itself an element of $B_{\infty ,T}$.

The condition $\omega _0(\mathcal {L}_{H_\mu +V_\mu } \, B)=0$ now becomes

20 $$\begin{aligned} \omega _0(\mathcal {L}_{\textrm{i}[K_\mu , H]}\, B) \,=\, - \omega _0(\mathcal {L}_{L_\mu + V_{\mu }} \, B) \, , \end{aligned}$$

where the right hand side of (20) depends only on $K_1,\ldots ,K_{\mu -1}$ and not on *m*. Recalling the definition of $\mathcal {I}$ (Definition 4.3) and applying Lemma 4.4, we can solve (20) inductively starting with

$$\begin{aligned} K_1:= -\mathcal {I}((X_1+V)) \end{aligned}$$

and then, given $K_1,\ldots , K_{\mu -1}$ and thus also $L_\mu$ and $V_\mu$,

21 $$\begin{aligned} K_\mu := -\mathcal {I}( L_\mu + V_{\mu }) \end{aligned}$$

for $\mu =2,\ldots ,m$. Note here, that $K_\mu$ is independent of $m\ge \mu$. Hence (21) indeed determines a sequence $(K_\mu )_{\mu \in \mathbb {N}}$ in $B_{\infty ,T}$.

We conclude that this choice for $(K_\mu )_{\mu \in \mathbb {N}}$ leads for every $m\in \mathbb {N}$ to

22 $$\begin{aligned} \sum _{k=0}^m \frac{1}{k!}\omega _0(\mathcal {L}_{\textrm{ad}({\textrm{i}S_\varepsilon ^{(m)}})^k H_\varepsilon } \, B) = \varepsilon ^{m+1} \omega _0(\mathcal {L}_{R^{(m)}_\varepsilon } \, B)\, , \end{aligned}$$

where $R^{(m)}_\varepsilon$ is bounded uniformly in $\varepsilon \in [-1,1]$ in any of the interaction norms $\Vert \cdot \Vert _\nu$. Thus, with Lemma 2.5, there exists $c_{m}$ such that for any $A\in D_\infty$

23 $$\begin{aligned} \begin{aligned} \left| \omega _0\left( \sum _{k=0}^m \frac{1}{k!} \mathcal {L}_{\text {ad}({\text {i}S_\varepsilon ^{(m)}})^k H_\varepsilon } A\right) \right|&{\mathop {\le }\limits ^{\textrm{Lemma}~2.5}} |\varepsilon |^{m+1} \left| \omega _0\left( \mathcal {L}_{R^{(m)}_\varepsilon } \, A\right) \right| \le |\varepsilon |^{m+1} \left\| \mathcal {L}_{R^{(m)}_\varepsilon }\,A \right\| \\  &{\mathop {\le }\limits ^{\text {Lemma}~2.5}}|\varepsilon |^{m+1} c \Vert R^{(m)}_\varepsilon \Vert _{d+1} \,\Vert A\Vert _{d+3 } \\  &{\mathop {\le }\limits ^{\textrm{Lemma}~2.5}} |\varepsilon |^{m+1} c_{m} \Vert {A}\Vert _{d+3}. \end{aligned} \end{aligned}$$

and also (using Lemmas 2.5 and  2.6)

24 $$\begin{aligned} \left| \omega _0\left( \beta _\varepsilon ^{(m)}(\tau ) \mathcal {L}_{\textrm{ad}({\textrm{i}S_\varepsilon ^{(m)}})^{m+1} H_\varepsilon } \beta _\varepsilon ^{(m)}(-\tau ) \beta _\varepsilon ^{(m)}(1)A\right) \right| \le |\varepsilon |^{m+1} c_{m}\Vert A\Vert _{d+3}\,. \end{aligned}$$

In summary, we find that for all $A\in D_\infty$

25 $$\begin{aligned} | \omega _0(\beta _\varepsilon ^{(m)}(1)\mathcal {L}_{H_{\varepsilon }}A) |\le 2 \,c_m|\varepsilon |^{m+1} \Vert A\Vert _{d+3}\,. \end{aligned}$$

Finally, we define

26 $$\begin{aligned} S_\varepsilon := \sum _{\mu =1}^\infty \chi (|\varepsilon |/\delta _\mu ) \,\varepsilon ^\mu K_\mu \end{aligned}$$

with $\chi (x):=\textbf{1}_{[0,1]}(x)$ the characteristic function of the interval [0, 1] and $(\delta _{\mu })_{\mu \in \mathbb {N}}$ the monotonically decreasing sequence defined by setting $\delta _1:=1$ and

$$\begin{aligned} \delta _\mu:= &   \min \left\{ \frac{1}{2^\mu }, \frac{1}{2\,\max \{1, \Vert K_1\Vert _\mu ,\ldots ,\Vert K_\mu \Vert _\mu \}}, \right. \nonumber \\    &   \left. \frac{1}{\max \{1, c_{1},\ldots ,c_{\mu }\}},\delta _1,\ldots ,\delta _{\mu -1} \right\} , \quad \mu > 1\,.\end{aligned}$$

In (26), the $\mu$-th term in the series is included as soon as $|\varepsilon |\le \delta _\mu$.

Let $\nu \in \mathbb {N}_{0}$. For $k > \nu$ and $\delta _{k+1} < |\varepsilon |\le \delta _k$

$$\begin{aligned} \Vert S_\varepsilon \Vert _\nu\le &   \sum _{\mu =1}^k |\varepsilon |^\mu \Vert K_\mu \Vert _\nu \\  \le &   |\varepsilon |\sum _{\mu =1}^k \delta _\nu ^{\mu -1} \Vert K_\mu \Vert _\nu \le |\varepsilon |\, \left( \Vert K_1 \Vert _\nu + \sum _{\mu =2}^k 2^{1-\mu }\right) \le |\varepsilon |\, (\Vert K_1 \Vert _\nu + 1) \,. \end{aligned}$$

For $$1 \le k \le \nu $$ and $$\delta _{k+1} < |\varepsilon |\le \delta _k$$

$$\begin{aligned} \Vert S_\varepsilon \Vert _\nu \le \sum _{\mu =1}^k |\varepsilon |^\mu \Vert K_\mu \Vert _\nu \le |\varepsilon |\sum _{\mu =1}^\nu \Vert K_\mu \Vert _\nu \,. \end{aligned}$$

Therefore, $\sup _{\varepsilon \in [-1,1]{\setminus } \{0\}} \frac{1}{|\varepsilon |}\Vert S_\varepsilon \Vert _\nu \le 1 + \sum _{\mu =1}^\nu \Vert K_\mu \Vert _\nu$ for all $\nu \in \mathbb {N}_{0}$.

With

$$\begin{aligned} C_n:= 2\,\max \left\{ \max _{k<n}\left\{ \frac{c_{k}\delta _k^{k+1}}{\delta _{k+1}^n}\right\} , 1 \right\} , \quad n \in \mathbb {N}, \end{aligned}$$

we find that for $\delta _{k+1} < |\varepsilon |\le \delta _k$ and $n\le k$

$$\begin{aligned} c_{k} |\varepsilon |^{k+1} \le c_{k} |\varepsilon |^{k+1-n} |\varepsilon |^n\le c_{k} |\varepsilon ||\varepsilon |^n\le c_{k} \delta _k |\varepsilon |^n \le |\varepsilon |^n\le \tfrac{1}{2}C_n |\varepsilon |^n, \end{aligned}$$

and that for $n>k$

$$\begin{aligned} c_{k} |\varepsilon |^{k+1} \le \frac{c_{k} \delta _k^{k+1}}{|\varepsilon |^n}|\varepsilon |^n\le \frac{c_{k} \delta _k^{k+1}}{\delta _{k+1}^n}|\varepsilon |^n\le \tfrac{1}{2}C_n|\varepsilon |^n. \end{aligned}$$

Hence, for $\delta _{k+1} < |\varepsilon |\le \delta _k$ and all $n\in \mathbb {N}$ it follows that

$$\begin{aligned} | \omega _0(\beta _\varepsilon \mathcal {L}_{H_{\varepsilon }}A) | = | \omega _0(\beta _\varepsilon ^{(k)}(1)\mathcal {L}_{H_{\varepsilon }}A) | \le 2 c_{k} |\varepsilon |^{k+1} \Vert A\Vert _{d+3} \le C_n|\varepsilon |^n\,\Vert A\Vert _{d+3}. \end{aligned}$$

$$\square $$

## Appendix G: Proof of Proposition 3.4

The proof is based on the results of [16] showing the stability of the gap for perturbations of free fermion systems in finite volume. Since suitable periodic finite volume approximations are only available for certain values of *b* which are rational multiples of $2\pi$, we use the following simple lemma on the persistence of the gap along approximating sequences twice: once to go from finite to infinite systems while keeping *b* fixed and rational (step 1 of the proof) and once to go from rational to irrational magnetic fields in infinite volume (step 2 of the proof).

### Lemma G.1

Let either $\tilde{\mathcal {A}}_n=\mathcal {A}_{\Lambda _{k_n}}$ for some strictly increasing sequence of boxes $\Lambda _{k_n}$, i.e. $k:\mathbb {N}\rightarrow \mathbb {N}$, $n\mapsto k_n$ is strictly increasing, or $\tilde{\mathcal {A}_n}=\mathcal {A}$ for all $n\in \mathbb {N}$. Let $(\omega _n)_{n\in \mathbb {N}}$ be a sequence of states on $\mathcal {A}$ and $(\mathcal {L}_{H_n})_{n\in \mathbb {N}}$ a sequence of derivations, each $\mathcal {L}_{H_n}$ acting on $\tilde{\mathcal {A}}_n$, such that for some $g>0$ and for all $n\in \mathbb {N}$ and all $A\in \tilde{\mathcal {A}}_n\cap \mathcal {A}_0$,

27 $$\begin{aligned} \omega _n( A^* \mathcal {L}_{H_n} A ) \ge g \left( \omega _n(A^* A) - \left| \omega _n(A) \right| ^2 \right) \,. \end{aligned}$$

If there exists a state $\omega$ and a derivation $\mathcal {L}_H$ on $\mathcal {A}$ such that for all $A\in \mathcal {A}_0$ it holds that $\lim _{n\rightarrow \infty }\omega _n(A)= \omega (A)$ and $\lim _{n\rightarrow \infty }\Vert (\mathcal {L}_{H_n}-\mathcal {L}_H)A\Vert =0$, then $\omega$ is a gapped ground state of $\mathcal {L}_H$ with gap *g*.

### Proof

Let $A\in \mathcal {A}_0$, thus $A\in \tilde{\mathcal {A}}_n$ for all *n* large enough. Letting $n \rightarrow \infty$ on the left hand side of (27) we find

$$\begin{aligned} \begin{aligned} |\omega _n(A^*\mathcal {L}_{H_n}A) - \omega (A^*\mathcal {L}_{H}A)|&\le |\omega _n(A^*(\mathcal {L}_{H_n}-\mathcal {L}_H)A)| + |(\omega _n- \omega )(A^*\mathcal {L}_{H}A)|\\  &\le \Vert A^*\Vert \,\Vert (\mathcal {L}_{H_n}-\mathcal {L}_H)A\Vert + |(\omega _n- \omega )(A^*\mathcal {L}_{H}A)|\\  &{\mathop {\rightarrow }\limits ^{n\rightarrow \infty }}0, \end{aligned} \end{aligned}$$

while the right hand side of (27) clearly converges to the corresponding expression with $\omega _n$ replaced by $\omega$. $\square$

### Proof of Proposition 3.4

As mentioned previously, we divide the proof into two steps.

**Step 1:** To apply the results of [16] showing the stability of the spectral gap of free Fermion systems under small perturbations, we restrict our system to a growing sequence of finite boxes $\Lambda _{k_n}$ in such a way that the gap remains open and the $T^b$-invariance is preserved for all $n\in \mathbb {N}$. To this end we first consider magnetic fields of the form $b=\frac{p}{q}2\pi$ for some $p\in \mathbb {Z}$ and some odd $q\in \mathbb {N}$. Then magnetic translations with respect to vectors $\gamma$ in the sub-lattice $\mathbb {Z}^2_q:= \mathbb {Z}\times (q \mathbb {Z})\subset \mathbb {Z}^2$ are ordinary translations, since the phase $b y_1 \gamma _2= by_1 m q= 2\pi p y_1 m\in 2\pi \mathbb {Z}$ in the definition of $t^b$ resp. $T^b$ (see (9)) is an integer multiple of $2\pi$ for all $y\in \mathbb {Z}^2$ and $m\in \mathbb {Z}$. This will allow us to apply Bloch–Floquet theory to $\mathfrak {h}_0^b$ with respect to the sub-lattice $\mathbb {Z}^2_q$ in order to relate the spectrum of $\mathfrak {h}_0^b$ to the spectra of its restrictions $\mathfrak {h}_{0,n}^b$ to suitable finite boxes $\Lambda _{k_n}$ defined below.

For $n\in \mathbb {N}$ let $k_n:= \frac{1}{2}((2n+1)q-1)\in \mathbb {N}$, so that the box size $2k_n+1= (2n+1)q$ is an integer multiple of *q*, and let $t^b|_{\Lambda _{k_n}}$ resp. $T^b|_{\Lambda _{k_n}}$ denote the magnetic translation acting on $\ell ^2(\Lambda _{k_n})$ resp. on $\mathcal {A}_{\Lambda _{k_n}}$ with periodic boundary conditions. More precisely, for $\gamma \in \mathbb {Z}^2$ we define $t^b|_{{\Lambda _{k_n}},\gamma }$ by

$$\begin{aligned} t^b|_{{\Lambda _{k_n}},\gamma }: \ell ^2(\Lambda _{k_n}) \rightarrow \ell ^2(\Lambda _{k_n}),\quad f\mapsto (t^b|_{{\Lambda _{k_n}},\gamma } f)(y):= \textrm{e}^{\textrm{i}b y_1 \gamma _2} f([y+\gamma ]_{\Lambda _{k_n}}) \end{aligned}$$

and $T^b|_{{\Lambda _{k_n}},\gamma }$ as the unique automorphism of $\mathcal {A}_{\Lambda _{k_n}}$ such that

$$\begin{aligned} T^b|_{{\Lambda _{k_n}},\gamma } (a_y) = \textrm{e}^{-\textrm{i}b y_1 \gamma _2} a_{[y+\gamma ]_{\Lambda _{k_n}}} \quad \forall \, y\in \Lambda _{k_n}, \end{aligned}$$

where $[y]_{\Lambda _{k_n}}$ is the unique element of $\Lambda _{k_n}$ such that $y-[y]_{\Lambda _{k_n}} \in (2k_n+1)\mathbb {Z}^2$. We define the finite volume Hamiltonians $\mathfrak {h}_{0,n}^b :\ell ^2(\Lambda _{k_n}) \rightarrow \ell ^2(\Lambda _{k_n})$ with integral kernel

$$\begin{aligned} \begin{aligned} \mathfrak {h}_{0,n}^b(x,y) \;{:}{=} {\left\{ \begin{array}{ll} \mathfrak {h}_{0}^b(x,y) &  \text{ if } \Vert x-y \Vert _1=1,\\ \mathfrak {h}_{0}^b(x,y+(2k_n+1,0)) &  \text{ if } x_1=k_n , \,y_1=-k_n,\\ \mathfrak {h}_{0}^b(x,y-(2k_n+1,0)) &  \text{ if } x_1=-k_n , \,y_1=k_n,\\ \mathfrak {h}_{0}^b(x,y+(0,2k_n+1)) &  \text{ if } x_2=k_n , \,y_2=-k_n,\\ \mathfrak {h}_{0}^b(x,y-(0,2k_n+1)) &  \text{ if } x_2=-k_n , \,y_2=k_n.\\ \end{array}\right. } \end{aligned} \end{aligned}$$

and for all $H^{b,\mu }_\lambda$ the restrictions

$$\begin{aligned} \begin{aligned} H_{\lambda ,n}^{b,\mu } \;{:}{=}\sum _{\gamma \in \Lambda _{k_n}} \sum _{M\in R_0(\mathbb {Z}^2) \cap \Lambda _{k_n}} T^b|_{\Lambda _{k_n},\gamma } \, H_{\lambda }^{b,\mu }(M) \, , \end{aligned} \end{aligned}$$

which are, by construction, $t^b|_{\Lambda _{k_n}}$ resp. $T^b|_{\Lambda _{k_n}}$-invariant. Note, moreover, that for all $n\in \mathbb {N}$ the non-interacting finite-volume Hamiltonian $H_{0,n}^{b,\mu }$ is the second quantization with chemical potential $\mu$ of $\mathfrak {h}_{0,n}^b$. Thus, according to [13, Example 5.3.20] the unique ground state $\omega _{0,n}$ of $H_{0,n}^{b,\mu }$ is the quasi-free state on $\mathcal {A}_{\Lambda _{k_n}}$ whose two-point function is the integral kernel of $P_{(-\infty ,\mu )}^{\mathfrak {h}^b_{0,n}}$, the Fermi projection corresponding to $\mathfrak {h}_{0,n}^b$. By uniqueness of the ground state and $T^b|_{\Lambda _{k_n}}$-invariance of $H_{0,n}^{b,\mu }$ it holds that $\omega _{0,n}$ is $T^b|_{\Lambda _{k_n}}$-invariant as well. This ground state is gapped with gap $\textrm{dist}(\mu , \sigma (\mathfrak {h}_{0,n}^{b}))$ in the sense of Definition 3.1^5^ and it holds that $\textrm{dist}(\mu , \sigma (\mathfrak {h}_{0,n}^{b}) )\ge \textrm{dist}(\mu , \sigma (\mathfrak {h}_{0}^{b}) )$ for all $n\in \mathbb {N}$. The last claim follows from the fact that all $\mathfrak {h}_{0,n}^{b}$ are $\mathbb {Z}^2_q$-periodic restrictions of the $\mathbb {Z}^2_q$-periodic operator $\mathfrak {h}_{0}^{b}$ and one finds in Bloch-Floquet representation that $\sigma (\mathfrak {h}_{0,n}^{b})\subset \sigma (\mathfrak {h}_{0}^{b})$ for all $n\in \mathbb {N}$.^6^

According to [16] there exists $\lambda _0>0$, independent of *n*, such that for $|\lambda |<\lambda _0$ and all *n* the operator $H_{\lambda ,n}^{b,\mu }$ has a unique ground state $\omega _{\lambda ,n}$ with gap $\frac{1}{2}\, \textrm{dist}(\mu , \sigma (\mathfrak {h}_{0,n}^{b}) )\ge \frac{1}{2}\, \textrm{dist}(\mu , \sigma (\mathfrak {h}_{0}^{b}) )$. By uniqueness, also $\omega _{\lambda ,n}$ is $T^b|_{\Lambda _{k_n}}$-invariant. By compactness of the set of states on $\mathcal {A}$ the sequence $n\mapsto \tilde{\omega }_{\lambda ,n} := \omega _{\lambda ,n}\circ \mathbb {E}_{\Lambda _{k_n}}$ has weak$^{*}$-limit points. Let $\omega$ be such a limit point. Then for any $A\in \mathcal {A}_0$ and $\gamma \in \mathbb {Z}^2$ there exists an $n_0 \in \mathbb {N}$ such that for all $n \ge n_0$ both *A* and $T_\gamma ^b\, A$ lie in $\mathcal {A}_{\Lambda _{k_n}}$. We thus have

$$\begin{aligned} \omega (T_\gamma ^bA)= &   \lim _{n\rightarrow \infty }\omega _{\lambda ,n}(\mathbb {E}_{\Lambda _{k_n}}T_\gamma ^bA) \nonumber \\  = &   \lim _{n\rightarrow \infty }\omega _{\lambda ,n}(T_\gamma ^b|_{\Lambda _{k_n}}A) = \lim _{n\rightarrow \infty }\omega _{\lambda ,n}(\mathbb {E}_{\Lambda _{k_n}} A )=\omega ( A), \end{aligned}$$

where, in order not to overload the notation, we have not made the choice of a convergent subsequence explicit.^7^ Thus the limit points are $T^b$-invariant. Moreover, since the strong convergence of $\mathcal {L}_{H_{\lambda ,n}^{b,\mu }}$ to $\mathcal {L}_{H_{\lambda }^{b,\mu }}$ on $\mathcal {A}_0$ follows from the definition of $H_{\lambda ,n}^{b,\mu }$, Lemma G.1 implies that each limit point of the sequence $(\tilde{\omega }_{\lambda ,n})_{n\in \mathbb {N}}$ is a gapped ground state for $H_\lambda ^{b,\mu }$ with gap $\frac{1}{2}\, \textrm{dist}(\mu , \sigma (\mathfrak {h}_{0}^{b}) )$.

Hence, for each value of *b* such that $\mu \notin \sigma (\mathfrak {h}_0^b)$ and $b/2\pi = \frac{p}{q}$ with $p\in \mathbb {Z}$ and $q \in \mathbb {N}$ odd, there exists $\lambda _0>0$ such that for each $|\lambda |<\lambda _0$ the interaction $H_\lambda ^{b,\mu }$ possesses at least one gapped $T^b$-invariant ground state with gap $\frac{1}{2}\,\textrm{dist}(\mu , \sigma (\mathfrak {h}_0^b))$.

**Step 2:** Let us now consider an arbitrary $b \in \mathbb {R}$ and $\mu \notin \sigma (\mathfrak {h}_0^b)$ and a $T^b$-invariant $V\in B_{\exp }$. We choose a sequence $(b_n)_{n\in \mathbb {N}}$ approximating *b* such that $\mu \notin \sigma (\mathfrak {h}_0^{b_n})$ and $b_n/2\pi = \frac{p_n}{q_n}$ with $p_n\in \mathbb {Z}$ and $q_n \in \mathbb {N}$ odd. This is possible, since $\{ (\mu ',b')\in \mathbb {R}^2\, |\, \mu ' \notin \sigma (\mathfrak {h}_0^{b'}) \}$ is an open set, see e.g. [3, 43]. Next we define the sequence of interactions $(V_n)_{n \in \mathbb {N}}$ as

$$\begin{aligned} \begin{aligned} V_n(M+\gamma ) \;{:}{=}\;T_\gamma ^{b_n} \,V(M) \quad \text{ for } \text{ all } \quad M\in R_0(\mathbb {Z}^2),\; \gamma \in \mathbb {Z}^2. \end{aligned} \end{aligned}$$

For all $n\in \mathbb {N}$, $\nu \in \mathbb {N}_0$ it holds that $\Vert V_n \Vert _\nu =\Vert V \Vert _\nu$ and $V_n$ is $T^{b_n}$-invariant, since *V*(*M*) is gauge invariant and thus $T^{b_n}$-compatible for all $M\in R_0(\mathbb {Z}^2)$. With this we define a sequence of interacting Hofstadter Hamiltonians for $\lambda \in \mathbb {R}$ that are $T^{b_n}$-invariant:

$$\begin{aligned} \begin{aligned} \tilde{H}^{b_n,\mu }_{\lambda } \;{:}{=}\;H^{b_n,\mu }_0 + \lambda \, V_n \, . \end{aligned} \end{aligned}$$

By **Step 1** we have that for $n\in \mathbb {N}$ there exists a $\lambda _{0,n}>0$, such that for $|\lambda |<\lambda _{0,n}$ the Hamiltonian $\tilde{H}^{b_n,\mu }_{\lambda }$ has at least one $T^{b_n}$-invariant gapped ground state with gap $\frac{1}{2} \textrm{dist}(\mu , \sigma (\mathfrak {h}_0^{b_n}))$. Note that $\lambda _{0,n}$ depends only on certain norms of $\mathfrak {h}_0^{b_n}-\mu$ and $V_n$ that are insensitive to complex phases and thus independent of *n*, and in a monotone way on $\textrm{dist}(\mu ,\sigma (\mathfrak {h}_0^{b_n}))$ (see [16, Theorems 3 and 11]).

Thus, we can find a uniform bound for $\lambda _{0,n}$ away from zero, if *n* is large enough: Since $\{ (\mu ',b')\in \mathbb {R}^2\, |\, \mu ' \notin \sigma (\mathfrak {h}_0^{b'}) \}$ is open, there is a $\delta >0$ such that the open ball $B_\delta (\mu ,b)$ with respect to $\Vert \cdot \Vert _\infty$ is still contained in the set. Hence, there exists an $n_0\in \mathbb {N}$ such that for all $n>n_0$ it holds that $\textrm{dist}(\mu , \sigma (\mathfrak {h}_0^{b_n})) \ge \delta$ and $\lambda _* {:}{=}\inf _{n>n_0} \lambda _{0,n} >0$. Therefore, if $|\lambda |< \lambda _*$ and $n>n_0$, we have that $\tilde{H}^{b_n,\mu }_{\lambda }$ has at least one $T^{b_n}$-invariant gapped ground state with gap $\frac{1}{2} \,\textrm{dist}(\mu , \sigma (\mathfrak {h}_0^{b_n})) \ge \frac{1}{2} \, \delta$.

Let $|\lambda |< \lambda _*$. By compactness, every sequence $(\omega _n)_{n\in \mathbb {N}}$, such that for $n>n_0$ it holds that $\omega _n$ is a $T^{b_n}$-invariant gapped ground state of $\tilde{H}^{b_n,\mu }_{\lambda }$ with gap $\frac{1}{2} \, \delta$, has a weak$^{*}$ limit point. Using that $\mathcal {L}_{\tilde{H}^{b_n,\mu }_{\lambda }}$ converges strongly to $\mathcal {L}_{H^{b,\mu }_{\lambda }}$, we conclude with Lemma G.1 that every limit point of such a sequence is a gapped ground state of $H^{b,\mu }_{\lambda }$ with gap $\frac{1}{2} \, \delta$. Since $T^{b_n}$ converges strongly to $T^b$, every such limit point $\omega$ is also $T^b$-invariant: for each $A\in \mathcal {A}_0$ and $\gamma \in \mathbb {Z}^2$

$$\begin{aligned} \begin{aligned} |\omega (T^b_\gamma A) - \omega (A)|&\le |(\omega - \omega _n)(T^b_\gamma A)| + |\omega _n(T^b_\gamma A - T^{b_n}_\gamma A)| + |\omega _n(T^{b_n}_\gamma A)- \omega (A)|\\  &\le |(\omega - \omega _n)(T^b_\gamma A)| + \Vert T^b_\gamma A - T^{b_n}_\gamma A\Vert + |\omega _n(A)- \omega (A)|\\  &{\mathop {\longrightarrow }\limits ^{n\rightarrow \infty }}0, \end{aligned} \end{aligned}$$

where again we did not make the choice of a convergent subsequence explicit. $\square$

## Footnotes

1 Note that the proof in [46] applies only to finite systems, but with bounds uniform in the system size, while the result in [28] assumes, in addition to our assumptions below, that $\omega _0$ is the unique ground state of $\mathcal {L}_H$. However, this assumption was not used for the statement about adiabatic switching that is relevant here. A version of the adiabatic theorem for NEASS, which in particular explicitly covers the Hamiltonians and perturbations considered in the present paper, but which has a much simplified proof compared to [28], is given in [10].

2 Strictly speaking $\textrm{i}[H,X_k]$ is a bounded SLT-interaction, see Sect. 2.1 for details.

3 In the following the term *automorphism* is used in the sense of a $*$-automorphism as defined in the textbook [14].

4 Here, the interaction *N* is referred to as the number operator for the following reason: The derivation generated by the second quantization operator $\textrm{d}\Gamma (\textbf{1})$ (commonly known as the number operator) coincides with the Liouvillian induced by the interaction *N*: $\mathcal {L}_N A = [\textrm{d}\Gamma (\textbf{1}), A]$ ($A \in D(\mathcal {L}_N)$). Similarly, the interaction $X_j$ is referred to as the position operator for analogous reasons.
